# The Impact of Oxidative Stress and AKT Pathway on Cancer Cell Functions and Its Application to Natural Products

**DOI:** 10.3390/antiox11091845

**Published:** 2022-09-19

**Authors:** Jun-Ping Shiau, Ya-Ting Chuang, Jen-Yang Tang, Kun-Han Yang, Fang-Rong Chang, Ming-Feng Hou, Ching-Yu Yen, Hsueh-Wei Chang

**Affiliations:** 1 Division of Breast Oncology and Surgery, Department of Surgery, Kaohsiung Medical University Hospital, Kaohsiung Medical University, Kaohsiung 80708, Taiwan or; 2 Graduate Institute of Medicine, College of Medicine, Kaohsiung Medical University, Kaohsiung 80708, Taiwan; 3 School of Post-Baccalaureate Medicine, Kaohsiung Medical University, Kaohsiung 80708, Taiwan; 4 Department of Radiation Oncology, Kaohsiung Medical University Hospital, Kaoshiung Medical University, Kaohsiung 80708, Taiwan; 5 Graduate Institute of Natural Products, Kaohsiung Medical University, Kaohsiung 80708, Taiwan; 6 Department of Biomedical Science and Environmental Biology, College of Life Science, Kaohsiung Medical University, Kaohsiung 80708, Taiwan; 7 Department of Oral and Maxillofacial Surgery, Chi-Mei Medical Center, Tainan 71004, Taiwan; 8 School of Dentistry, Taipei Medical University, Taipei 11031, Taiwan; 9 Center for Cancer Research, Kaohsiung Medical University, Kaohsiung 80708, Taiwan

**Keywords:** AKT signaling, oxidative stress, natural product, cell function

## Abstract

Oxidative stress and AKT serine-threonine kinase (AKT) are responsible for regulating several cell functions of cancer cells. Several natural products modulate both oxidative stress and AKT for anticancer effects. However, the impact of natural product-modulating oxidative stress and AKT on cell functions lacks systemic understanding. Notably, the contribution of regulating cell functions by AKT downstream effectors is not yet well integrated. This review explores the role of oxidative stress and AKT pathway (AKT/AKT effectors) on ten cell functions, including apoptosis, autophagy, endoplasmic reticulum stress, mitochondrial morphogenesis, ferroptosis, necroptosis, DNA damage response, senescence, migration, and cell-cycle progression. The impact of oxidative stress and AKT are connected to these cell functions through cell function mediators. Moreover, the AKT effectors related to cell functions are integrated. Based on this rationale, natural products with the modulating abilities for oxidative stress and AKT pathway exhibit the potential to regulate these cell functions, but some were rarely reported, particularly for AKT effectors. This review sheds light on understanding the roles of oxidative stress and AKT pathway in regulating cell functions, providing future directions for natural products in cancer treatment.

## 1. Introduction

The AKT (AKT serine-threonine kinase; protein kinase B; PKB) pathway, which consists of AKT and AKT downstream effectors, is involved in regulating many cell functions such as cell survival, proliferation, metabolism [1], angiogenesis, and migration [2], by activating AKT [3,4]. AKT is commonly overexpressed in several kinds of cancer [5].

AKT activity is modulated by phosphorylation and dephosphorylation for activation and inactivation [6]. AKT is activated through several routes, mainly by ligand–receptor tyrosine kinase phosphorylation that activates phosphoinositide 3-kinase (PI3K) and consequently AKT [7]. Growth factors and cytokines are common ligands for AKT activation [8]. Furthermore, AKT is also activated by cellular stressors, such as heat shock [9], ultraviolet irradiation [10], and hypoxia [11].

AKT is also known to control the expression of several AKT effectors, including forkhead box transcription factors (FOXO), c-Myc, hypoxia-inducible factor (HIF), the mechanistic target of the rapamycin complex 1/2 (mTORC1/2), mTOR substrate S6 kinase 1/2 (S6K1/2), sterol regulatory element-binding protein 1 (SREBP1) [12], and glycogen synthase kinase 3 (GSK3) [1,13]. Hence, the impact of AKT effectors on regulating cell function warrants a more detailed assessment.

Oxidative stress is the status where cells exhibit higher reactive oxygen species (ROS) levels than antioxidants, causing an imbalance of redox homeostasis [14,15]. ROS include non-radical and radical chemical species. Examples of non-radical ROS include organic hydroperoxides (ROOH), singlet molecular oxygen (O_2_), electronically excited carbonyl, ozone (O_3_), and hypochlorous and hypobromous acid (HOCl and HOBr). Examples of free-radical ROS include superoxide anion radical (O_2_·−), hydroxyl radical (·OH), peroxyl radical (ROO·), and alkoxyl radical (RO·). ROS are generated as by-products of several cell functions, such as energy production. Moreover, ROS is also generated by exposure to drugs, toxins, and radiation [16].

Oxidative stress may modulate drug-activated phosphoinositide 3-kinase (PI3K)/AKT. PI3K/AKT/mTOR shows crosstalk with oxidative stress and DNA damage response in cancer cells [17]. PI3K/AKT/mechanistic target of the rapamycin kinase (mTOR) is responsive to maintain a redox metabolism in cancer [18]. 

**Figure 1:**
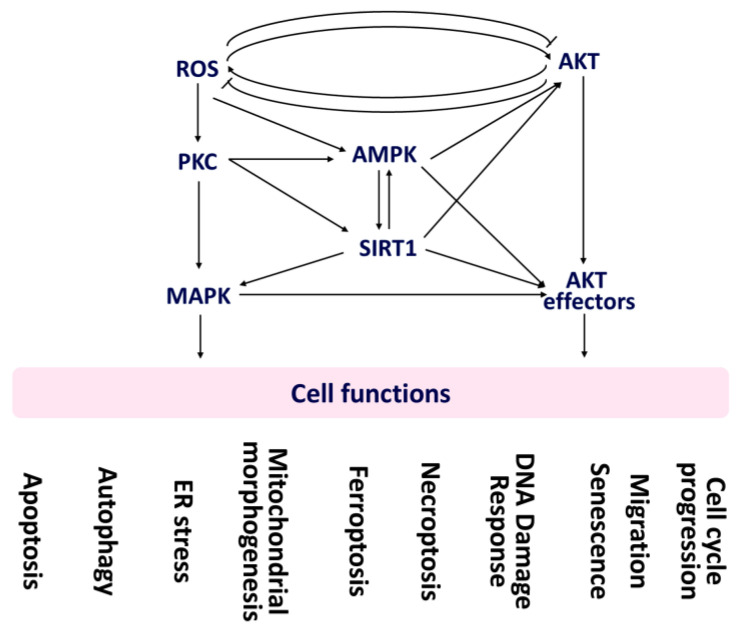
Network for oxidative stress and AKT pathway regulations of cell functions in cancer cell treatments. Several AKT effectors are included in this review, such as forkhead box transcription factors (FOXO), c-Myc, hypoxia-inducible factor (HIF), mechanistic target of rapamycin complex 1/2 (mTORC1/2), mTOR substrate S6 kinase 1/2 (S6K1/2), sterol regulatory element-binding protein 1 (SREBP1), and glycogen synthase kinase 3 (GSK3). Several cell functions are included, such as apoptosis, autophagy, ER stress, mitochondrial morphogenesis, ferroptosis, necroptosis, DNA damage response, senescence, migration, and cell-cycle progression.

Different treatments may show different responses to oxidative stress and AKT activation. Notably, the following examples from various cell lines demonstrate the potential for interaction between oxidative stress and AKT activation. This needs careful investigation in case other cell lines are concerned because their genetic mutations may differ. Four responses to oxidative stress and AKT, namely (1) oxidative stress activates AKT, (2) oxidative stress inhibits AKT, (3) AKT induces oxidative stress, and (4) AKT suppresses oxidative stress, were summarized as follows (Figure 1).

Excessive oxidative stress can activate AKT [18] (Figure 1). 4-Hydroxyestradiol activates AKT in epithelial cells, which is otherwise suppressed by oxidative stress inhibitor and AKT siRNA [19]. PM_2.5_ induces ROS generation and AKT activation in endothelial cells, which were suppressed by *N*-acetylcysteine, suggesting that PM_2.5_-induced oxidative stress can activate AKT [20]. Oxidative stress activates PI3K/AKT [21] to trigger apoptosis in chondrocytes [22]. Mitochondrial superoxide activates PI3K/AKT and mTORC1 to induce autophagy during muscle differentiation [23]. 

Oxidative stress may inhibit AKT expression (Figure 1). Nexrutine^R^ [24] and kaempferol [25] cause oxidative stress in melanoma and pancreatic cancer cells, respectively. This inactivates PI3K/AKT/mTOR signaling, which is reverted by the pre-treatment of oxidative stress inhibitor *N*-acetylcysteine. Baicalin triggers apoptosis and autophagy of osteosarcoma cells by up-regulating ROS and down-regulating PI3K/AKT/mTOR [26].

AKT may induce oxidative stress in cancer cells [27] (Figure 1). AKT activates NADPH oxidases (NOXs) to enhance ROS generation in cancer cells [18]. In contrast, AKT inhibitor enhances chloroquine-induced cellular ROS and mitochondrial superoxide generation of prostate cells, suggesting AKT may suppress oxidative stress [28] (Figure 1).

Accordingly, oxidative stress and PI3K/AKT/mTOR show an interplay relationship and modulate several cellular stress responses such as apoptosis [23,26] and autophagy [26,29] (Figure 1). In addition to apoptosis and autophagy, the regulations between oxidative stress and AKT in regulating other cell functions are also discussed in this review, such as endoplasmic reticulum (ER) stress, mitochondrial morphogenesis (fission and fusion), ferroptosis, necroptosis, senescence, migration, and cell-cycle progression (Figure 1). Brief overviews of these cell functions will be given below (Section 2.1, Section 2.2, Section 2.3, Section 2.4, Section 2.5, Section 2.6, Section 2.7, Section 2.8, Section 2.9 and Section 2.10).

Natural products may generate oxidative stress [30] and modulate AKT expression [31,32] in cancer cell treatments. However, the potential regulation of cell functions by natural product-modulating oxidative stress and AKT pathway (AKT and AKT effectors) lacks systemic understanding. The modulating effects of natural products on oxidative stress and the AKT pathway will be discussed later.

The impacts of oxidative stress (Section 2), AKT (Section 3), and AKT effectors (Section 4) on regulating cell functions for cancer cells are illustrated in Figure 1. This review also discusses the impact of several natural product-modulating oxidative stress (Section 5), AKT (Section 6), and AKT effectors (Section 7) on regulating cell functions for cancer cell treatments.

## 2. Oxidative Stress Modulates Cell Functions

Oxidative stress provides a complex network regulating the AKT pathway (AKT and AKT effectors) and controlling different cell functions. The relationship between oxidative stress and cell functions is discussed below (Section 2.1, Section 2.2, Section 2.3, Section 2.4, Section 2.5, Section 2.6, Section 2.7, Section 2.8, Section 2.9 and Section 2.10) (Table 1).

### 2.1. Apoptosis and Oxidative Stress

Oxidative stress induction by drugs has become a common strategy in cancer therapy [83,84,85,86] because oxidative stress may contribute to early apoptosis [87] and leads to mitochondrial dysfunction [88,89,90]. Oxidative stress generations by various anticancer drugs or phytochemicals are closely related to apoptosis induction in cancers [91].

Interestingly, oxidative stress may regulate several mediators and affect apoptosis (Figure 1, Table 1). For example, oxidative stress may activate protein kinase C (PKC) [33,34]. PKC may regulate sirtuin 1 (SIRT1) [92], a class III protein deacetylase. SIRT1 is an important survival protein in regulating oxidative stress [35]. Moreover, PKC induces mitogen-activated protein kinase (MAPK) activation, namely the PKC-MAPK pathway [36]. SIRT1 overexpression can activate MAPK, such as extracellular-regulated kinase (ERK) [37], which may inhibit apoptosis [93]. Alternatively, another MAPK p38 may enhance apoptosis [38] by regulating the PI3K/AKT expression [39].

### 2.2. Autophagy and Oxidative Stress

Autophagy is an intracellular catabolic process where long-lived proteins and dysfunctional organelles are degraded for recycling to generate energy under nutrient depletion or stress [94]. Oxidative stress controls autophagy in modulating cell survival and development [95,96]. The interaction between oxidative stress and autophagy in cancer cells has been reported from tumor initiation to cancer therapy [97].

Oxidative stress may regulate several mediators and affect autophagy (Figure 1, Table 1). Oxidative stress may regulate MAPK [98] to modulate autophagy [40]. Similarly, reactive oxygen species (ROS)-modulating drugs such as juglanin induce autophagy in human breast cancer progression via ROS/c-Jun N-terminal kinases (JNKs) promotion [41]. p38 MAPK suppresses autophagy by activating Unc-51-like kinase 1 (ULK1) [42].

Moreover, MAPK may interact with AMPK [43]. AMPK may cooperate with ULK1 and mTORC1 to regulate autophagy [44,45]. Therefore, ROS is essential in regulating autophagy (Figure 1).

### 2.3. ER Stress and Oxidative Stress

ER is a dynamic organelle involving several cellular functions [99,100]. Three primary unfolded protein response (UPR) mediators are identified, including protein kinase-RNA-like ER kinase (PERK), inositol-requiring enzyme 1 alpha (IRE1α), and activating transcription factor 6 (ATF6) [46] (Table 1). Under cell stress, the ER environment is unstable, protein maturation is dysfunctional, and misfolded protein accumulates, which triggers UPR to recover normal ER function by attenuating protein translation, enhancing degradation of misfolded proteins, and up-regulating molecular chaperones for protein folding [99,100].

Many drugs possess modulating effects of oxidative stress and ER stress [47] for cancer cell death. For example, curcumin analog WZ35 may induce oxidative stress-dependent ER stress and G2/M arrest, leading to cell death of prostate cancer cells [101]. Oxidative stress may regulate several mediators and affect ER stress (Figure 1, Table 1). MAPK signaling can regulate ER stress response [48]. Consequently, ER stress can induce apoptosis via MAPK p38 and JNK [49].

### 2.4. Mitochondrial Morphogenesis and Oxidative Stress

Mitochondria have comprehensive redox homeostasis and apoptosis functions in cancer cells [102]. Mitochondrial morphogenesis is a dynamic interchange process between mitochondrial fission and fusion [103], regulated by several mediators [104]. For example, the fusion-related proteins include mitofusin 1 and 2 (MFN1/MFN2) and optic atrophic protein 1 (OPA1) [105]. Fission-related proteins have mitochondrial fission protein 1 (FIS1), dynamin-related protein1 (DRP1 or DNM1L), and mitochondrial fission factor (MFF) [106,107,108].

Oxidative stress may regulate several mediators and affect mitochondrial morphogenesis (Figure 1, Table 1), leading to apoptosis and cell death [109]. For example, ROS activates mitochondrial fission through DRP1 [50]. DRP1 interacts with FIS1 to cause mitochondrial fission and oxidative stress [51]. MFN2 is required for ROS production and inflammation in macrophages [110]. siMFN1 induces ROS generation of myoblast cells, which is suppressed by MFN1 overexpression [52]. Moreover, high glucose could cause oxidative stress in renal tubular epithelial cells and trigger mitochondrial fission and apoptosis [104]. Accordingly, inhibiting mitochondrial fission and enhancing mitochondrial fusion prevents apoptosis [111], whereas induction of mitochondrial fission promotes apoptosis [104].

Moreover, MAPK activation causes mitochondrial fission (Figure 1*,* Table 1). For example, JNK activation can phosphorylate MFN2 for its ubiquitin-proteasome degradation and induce mitochondrial fission in sarcoma U2OS cells [53]. MAPK, such as ERK2 and p38 activation, can phosphorylate DRP1, causing mitochondrial fission in mesenchymal stem cells [54]. 

### 2.5. Ferroptosis and Oxidative Stress

Ferroptosis is a distinct type of cell death associated with oxidative stress-induced iron uptake, lipid peroxidation, and glutathione peroxidase 4 (GPX4) down-regulation [112]. Drug-inducing oxidative stress for ferroptosis is a novel strategy in cancer therapy [113,114]. Several mediators of ferroptosis, such as GPX4, ACSL4, prostaglandin-endoperoxide synthase 2 (PTGS2; COX2), and CHAC1, have been identified [115], and their impact on autophagy was described (Figure 1, Table 1).

Accumulating evidence demonstrates that ferroptosis-based cell death causes antiproliferation of cancer cells (Table 1). Ferroptosis inducer RSL3 induces ROS generation and inhibits GPX4 to trigger ferroptosis in colon cancer cells [55]. Ginsenoside Re, a ginseng-derived compound, inhibits 6-hydroxydopamine-promoted oxidative stress of neuroblastoma cells by increasing GPX4 expression [116]. In contrast, GPX4 down-regulation improves oxidative stress-induced cell death of chondrocytes [56].

Down-regulation of acyl-CoA synthetase long-chain family member 4 (ACSL4) alleviates oxidative stress to suppress stroke-induced ferroptosis and recover neurological function (Table 1) [57]. Similarly, ACSL4 knockdown decreases oxidative stress and improves the survival of neurons [58]. Furthermore, oxidative stress activates transforming growth factor-β-activated kinase 1 (TAK1) to phosphorylate MAPK and NF-κB, inducing PTGS2 expression, which is reverted by *N*-acetylcysteine [59]. Albumin-induced oxidative stress also up-regulates PTGS2 signaling in proximal tubular cells [117]. Down-regulation of autophagy activates the ROS-MAPK1/3 axis to up-regulate PTGS2 expression [60]. Overexpression of ChaC glutathione-specific gamma-glutamylcyclotransferase 1 (CHAC1), a ROS sensor, dramatically depletes glutathione [61], which may induce oxidative stress. Dihydroartemisinin up-regulates CHAC1 expression to induce ferroptosis of liver cancer cells [62]. Accordingly, oxidative stress induces PTGS2 expression, and ACSL4 and CHAC1 cause oxidative stress, while GPX4 inhibits oxidative stress (Table 1). NFE2-like bZIP transcription factor 2 (NFE2L2; NRF2) is an essential transcription factor of oxidative stress that regulates ferroptosis involving glutathione modulation [63]. 

Moreover, MAPK activation contributes to ferroptosis (Figure 1, Table 1) and its associated cell death [118]. For example, severe cold stress induces ferroptosis [64]. The potent ferroptosis inducer erastin is also associated with MAPK activation [119] and activates p38 downstream of lipid peroxidation [64].

### 2.6. Necroptosis and Oxidative Stress

Oxidative stress may regulate several mediators and trigger necroptosis (Table 1) [66,67]. Necrotic cells are characterized by cell swelling and poor plasma membrane integrity [120]. Necroptosis is a programmed type of necrosis. Receptor-interacting serine/threonine-protein kinase 1 (RIPK1), RIPK3, and mixed lineage kinase domain such as pseudokinase (MLKL) are modulators for the necroptotic process [65]. 

For example, RETRA, the small molecule regulating the REactivation of Transcriptional Reporter Activity, can induce p53-associated gene expressions. RETRA promotes necroptosis in cervical cancer cells by phosphorylating RIPK1, RIPK3, and MLKL and inducing oxidative stress generation, which is reverted by necrostatin-1 [121]. Heat stress causes intestinal injury by up-regulating RIPK1/RIPK3 and inducing necroptosis, which is suppressed by oxidative stress scavenger *N*-acetylcysteine [122].

MAPK has a modulating ability for necroptosis (Figure 1). For example, sulforaphane, a cruciferous vegetable-derived compound, suppresses necroptosis of microglia-mediated neuron damage by inhibiting MAPK expression [123]. Dimethyl fumarate, a drug for treating multiple sclerosis, promotes necroptosis of colon cancer cells by inducing oxidative stress and activating MAPK [124].

### 2.7. DNA Damage Response and Oxidative Stress

Oxidative stress induces DNA damage [125] and DNA damage response (DDR) [126] in cancer cells [127]. Oxidative stress and DDR responses affect several mediators, such as MAPK ERK 1/2 and p38 [68] (Figure 1, Table 1). Moreover, ERK is triggered by oxidative stress to inhibit cell proliferation during DDR [68].

Moreover, oxidative stress can modulate some DNA repair machinery (Table 1). For example, oxidative stress inhibits 8-oxoguanine DNA glycosylase 1 (OGG1), which is an initiator of base excision repair (BER) [69]. Thioredoxin, a redox protein, can down-regulate apurinic/apyrimidinic endonuclease 1 (APE1), a BER protein [70]. Down-regulation of MAPK p38-dependent excision repair cross-complementing 1 (XRCC1) suppresses DNA repair function [128]. Accordingly, oxidative stress and DNA repair affect several signaling pathways involving MAPK (Figure 1).

### 2.8. Senescence and Oxidative Stress

Cellular senescence arrests the cycling of damaged cell proliferation as a tumor suppressor mechanism. ROS induces senescence in several cell types [129,130]. For example, SIRT1, SIRT3, and SIRT6 may inhibit vascular senescence [71] (Table 1).

Moreover, MAPK also regulates senescence (Figure 1, Table 1) [72,131]. Sirtinol, a SIRT1 inhibitor, causes senescence of cancer cells by down-regulating Ras–MAPK [131]. Busulfan-promoted senescence of diploid WI38 fibroblasts by inducing ROS and activating MAPK, reverted by *N*-acetylcysteine [72].

### 2.9. Migration and Oxidative Stress

Oxidative stress can regulate several mediators affecting cell migration (Figure 1, Table 1). For example, ERK that belongs to the MAPK family may regulate cell migration [93]. SIRT1 is a class III protein deacetylase that controls oxidative stress [35]. SIRT1 overexpression can activate MAPK/ERK signaling [37]. SIRT1 induces epithelial-mesenchymal transition (EMT) in melanoma cells by down-regulating E-cadherin (CDH1) and up-regulating N-cadherin (CDH2) and vimentin (VIM) [73].

Moreover, oxidative stress, MAPK/ERK, and protein kinase C zeta (PKCζ) play essential roles in regulating epidermal growth factor (EGF)-stimulated proliferation and migration of human corneal cells [74]. Moreover, PKC also phosphorylates AMPK [75]. SIRT1 can activate AMPK and vice versa [132]. Therefore, drug-induced oxidative stress overexpression also activates AMPK to suppress EMT and migration in cancer cells (Figure 1, Table 1).

### 2.10. Cell-Cycle Progression and Oxidative Stress

The involvement of oxidative stress-modulating AMPK, SIRT1, and MAPK in several functions was mentioned in Table 2. Notably, AMPK, SIRT1, and MAPK can regulate cell-cycle progression. Metformin causes G1 arrest of leukemia cells depending on AMPK activation and cyclin D1 down-regulation [76]. AMPK [77] and SIRT1 [79] also regulate the phosphorylation of CDK inhibitor p27^kip1^. Moreover, SIRT1 governs cell-cycle progression by modulating acetylation and phosphorylation of checkpoint kinase 2 (CHK2) [78]. SIRT1 silencing causes G1 arrest, accompanied by down-regulating CDK2/4/6 [80]. MAPK can modulate cell-cycle arrest [81]. MAPK is also tightly regulated with cyclin-dependent kinase (CDK) to control cell-cycle progression [82].

## 3. AKT Modulates Cell Functions

In addition to oxidative stress, AKT exhibits a complex network, such as MAPK, AMPK, and SIRT1, to regulate its downstream signaling (AKT effectors) (Figure 1). AKT can interplay with MAPK and AKT [164,165,166]. For example, AKT phosphorylates RAF protein kinase to inhibit MAPK expression [167]. AZD6244 inactivates ERK by activating PI3K/AKT [168]. Moreover, MAPK may interact with AMPK [43]. ROS may regulate AMPK [169] to modulate PI3K/AKT signaling [170]. SIRT1 can reciprocally regulate AMPK [132]. SIRT1 also activates PI3K/AKT [171]. This AKT network is summarized (Figure 1). Detailed information for AKT-regulated cell functions is summarized in Section 3.1, Section 3.2, Section 3.3, Section 3.4, Section 3.5, Section 3.6, Section 3.7, Section 3.8, Section 3.9 and Section 3.10 (Table 2).

### 3.1. Apoptosis and AKT

The impact of AKT on regulating apoptosis and several apoptosis mediators is summarized (Figure 1, Table 2). NPRL-Z-1, a topoisomerase II poison, triggers apoptosis and ROS production of renal cancer cells by inhibiting AKT [172]. PI3Kα inhibitor DFX24 shows antiproliferation and apoptosis of lung cancer cells by inhibiting PI3K/AKT and ERK but promoting EPH receptor B6 (EPHB6) expression [141]. AKT activates mTOR [142]. In addition to autophagy, mTOR also regulates apoptosis [143].

Moreover, Ki8751, a vascular endothelial growth factor (VEGF) receptor 2 inhibitor, induces ROS generation and apoptosis of breast cancer cells by decreasing the phosphorylation of AKT and peroxisome proliferator-activated receptor-γ coactivator 1-α (PGC1α), which improves the nucleus translocation of PGC1α and mitochondrial transcription factor A (TFAM) expression, and mitochondrial biogenesis [173]. 14-(3-Fluorophenyl)-8,13,13b,14-tetrahydroindolo[2′,3′:3,4]pyrido[2,1-b]quinazolin-5(7H)-one, an evodiamine derivative, triggers apoptosis of gastric cancer cells by suppressing PI3K/AKT [174].

### 3.2. Autophagy and AKT

AKT shows the impact on modulating autophagy, and several autophagy mediators are summarized (Figure 1, Table 2). Antiproliferation effects of anticancer drugs result from targeting PI3K/AKT/mTOR-mediated autophagy [144]. W922, a PI3K/AKT/mTOR inhibitor, induces antiproliferation and autophagy of colon cancer cells by down-regulating PI3K/AKT/mTOR [175]. ULK1 knockdown inactivates AKT-FOXO3a signaling and down-regulates hepatic mevalonate/cholesterol biosynthesis gene expressions [145].

### 3.3. ER Stress and AKT

AKT can regulate ER stress (Figure 1). Bardoxolone methyl, an NRF2 activator and NF-κB inhibitor, promotes ER stress, apoptosis, and autophagy in chronic myeloid leukemia cells, accompanied by inactivating PI3K/AKT/mTOR and p38 MAPK but activating ERK expression [176]. Tunicamycin induces ER stress by decreasing RIPK1 expression and inactivating AKT/mTOR in melanocytes, reverted by RIPK1 overexpression [177].

Several mediators of ER stress have been reported, such as glucose-regulated protein 78 (GRP78; HSPA5), IRE1, PERK, and ATF6 (Table 2). These ER stress mediators exhibit distinct modulation by PI3K/AKT/mTOR. Several examples of ER stress-modulating effects of PI3K/AKT/mTOR are known (Table 2).

GRP78 modulates either unfolded protein response (UPR) or induces PI3K/AKT activation for pro-survival [146]. GRP78 binds to IRE1, PERK, and ATF6 in normal conditions but dissociates at acute ER stress [146]. AKT-mTOR can inactivate IRE1 [178]. However, IRE1 also inactivates AKT to trigger apoptosis of hepatocytes, associated with ER stress [147]. PERK is essential to AKT activation during ER stress [148]. Inhibition of ATF6 suppresses subtilase cytotoxin-activated AKT [179]. Similarly, ATF6a can phosphorylate and activate AKT in intestinal epithelial cells [149]. ER stress also inactivates AKT to up-regulate CHOP expression and induce cell death [150]. Alkylphosphocholine erufosine, an AKT-mTOR inhibitor, induces ROS generation and ER stress in oral cancer cells [180].

### 3.4. Mitochondrial Morphogenesis and AKT

AKT can control mitochondrial fission/fusion (Figure 1). Arsenic and copper, robust oxidative stress inducers, promote mitochondrial fission in chicken skeletal muscle by up-regulating dynamin-related protein-1 (DRP1), down-regulating mitochondrial fusion genes, and inhibiting PI3K/AKT/mTOR [181]. Breast cancer cells stably expressing phosphatidylserine decarboxylase provide mitochondrial fission, accompanied by inactivating AKT and ERK [182]. Fissioned mitochondria-inhibited systemic metastasis is suppressed by leflunomide, a potent activator of mitochondrial fusion proteins.

Several mediators of mitochondrial morphogenesis (fission and fusion) have been reported, such as DRP1, FIS1, MFN1, and MFN2 (Table 2). These mitochondrial morphogenesis mediators show a distinct modulation by PI3K/AKT/mTOR. Several examples of mitochondrial morphogenesis-modulating effects of PI3K/AKT/mTOR were described as follows.

AKT modulates mitochondrial fusion and fission proteins to regulate mitochondrial morphogenesis (Table 2) [183]. Amyloid-β induces sustained AKT activation to activate DRP1, leading to mitochondrial fission and neuronal apoptosis [151]. Carbon monoxide (CO) releasing molecule-2 (CORM2), a CO producer, inhibits mitochondrial fission by activating AKT and suppressing FIS1 expression in lipopolysaccharide (LPS)-treated alveolar macrophages [152]. Inhibition of MFN1 suppresses AKT-associated mitochondrial fusion in mouse embryonic fibroblasts [153]. Similarly, MFN2 knockdown in embryonic stem cells inactivates AKT [154]. This suggests that AKT induces mitochondrial fusion depending on MFN1 and MFN2.

### 3.5. Ferroptosis and AKT

AKT shows impacts on the modulation of ferroptosis (Figure 1). Lung cancer cells down-regulate ferroptosis by up-regulating PI3K/AKT/mTOR [184]. Several mediators of ferroptosis have been reported, such as GPX4, ACSL4, PTGS2, and CHAC1 [115]. These ferroptosis mediators exhibit a distinct modulation by PI3K/AKT/mTOR.

Several examples of ferroptosis-modulating effects of PI3K/AKT/mTOR were described (Table 2). Insulin induces ROS production and GPX4 expressions of breast cancer cells, suppressed by PI3K inhibitor LY294002. This finding indicates that PI3K/AKT is activated by insulin to down-regulate GPX4 [155]. AKT inhibition by MK2206 up-regulates ACSL4 expression in sh-SIRT3 gallbladder cancer cells to induce ferroptosis and suppresses EMT expression and cell migration [156]. PTGS2 can phosphorylate and activate AKT/NF-κB. MiR-124-3p exhibits antiproliferation and down-regulates PTGS2 expression of prostate cancer cells by inactivating AKT signaling [157]. Inhibition of CHAC1, an early ferroptosis mediator, decreases the cell viability of uveal melanoma cells by inactivating AKT/mTOR [158].

### 3.6. Necroptosis and AKT

AKT shows impacts on modulating necroptosis (Figure 1). FTY720, a potent immunosuppressant, induces apoptosis, autophagy, and necroptosis in glioblastoma cells [185] by suppressing PI3K/AKT/mTOR/p70S6K but inducing ROS-JNK-p53 signaling [185]. Several mediators of necroptosis have been reported, such as RIPK1, RIPK3, and MLKL. These necroptosis mediators show distinct modulations by PI3K/AKT/mTOR. Auranofin, a rheumatoid arthritis drug, has been validated to repurpose application to trigger apoptosis and necroptosis in auranofin-sensitive lung cancer cells, accompanied by suppressing the PI3K/AKT/mTOR pathway. Moreover, thioredoxin reductase 1 (TXNRD1) can reverse the auranofin-induced PI3K/AKT/mTOR inactivation [186].

Several examples of necroptosis-modulating effects of PI3K/AKT/mTOR were described as follows (Table 2). AKT activation suppresses RIPK1 inhibitor-induced antiproliferation of liver cancer cells [159], suggesting that RIPK1 acts as an upstream activator to up-regulate AKT expression. Moreover, AKT is activated by RIPK1 during necroptosis, partly mediated by mTORC1, and connects RIPK1 to JNK activation [187]. Inhibition of RIPK1 with necrostatin-1 inactivates AKT/mTOR in palmitic acid-treated cardiomyocytes [188], supporting the rationale that RIPK1 activates PI3K/AKT/mTOR signaling. RIPK3 induces kidney fibrogenesis by activating AKT and ATP citrate lyase (ACL), suppressed by RIPK3 knockdown [160]. Additionally, RIPK3 is also a modulator of necroptosis, acting as an upstream regulator to MLKL [161].

### 3.7. DNA Damage Response and AKT

AKT also controls DNA damage responses such as DNA damage/repair (Figure 1, Table 2). This concept was supported by several reports as follows. TH588 (a MutT homolog 1 (MTH1) inhibitor) and BKM120 (a pan-PI3K inhibitor) combined treatment promotes DNA damage and apoptosis by activating PI3K/AKT/mTOR in glioma cells [189]. Various PI3K isoforms differentially regulate the cell cycle, DNA replication, and DNA damage repair [190].

Several mediators of DNA damage response have been reported (Table 2). PI3K/AKT/mTOR can activate DNA-dependent protein kinase, catalytic subunit (DNA-PKcs) kinase to enhance proliferation, radioresistance, and DNA double-strand break (DSB) repair (Table 2) [162]. Moreover, the MRN complex consisting of meiotic recombination 11 (MRE11), RAD50 double-strand break repair protein (RAD50), and nibrin (NBS1) is the DSB sensor that recruits ATM serine/threonine kinase (ATM). ATM was reported to phosphorylate and activate AKT1 [163]. MK-2206 (AKT inhibitor) enhances the cell-killing effects of topoisomerase II inhibitors acting on soft tissue sarcomas and gastrointestinal stromal tumors by promoting DNA damage (γH2AX) and down-regulating homology recombination repair (RAD51) (Table 2) [165]. Xeroderma pigmentosum, complementation group C (XPC) is a nucleotide excision repair (NER) accessory protein. XPC silencing down-regulates AKT/mTOR [166].

Moreover, combined with radiation, PKI-587, a dual PI3K/mTOR inhibitor, suppresses cell proliferation and tumor growth of liver cancer, accompanied by apoptosis. This PKI-587/radiation combined treatment inhibits PI3K/AKT/mTOR and homologous recombination (HR) repair-related kinases such as the ATM and the ATM and Rad3-related (ATR) in liver cancer cells (Table 2) [164]. PI3K signaling is overexpressed in ovarian cancer, contributing to chemoresistance, DNA replication, and genome stability. AKT improves DSB repair of non-homologous end joining (NHEJ) mediated by DNA-PK [191] (Table 2). Therefore, the inactivation of PI3K may inhibit DNA repair from improving ovarian cancer therapy [192].

### 3.8. Senescence and AKT

AKT can regulate senescence (Figure 1). Endothelial cells inhibit senescence-related inflammation by inactivating PI3K/AKT/mTOR [193]. Estrogen in breast cancer 1 (GREB1) overexpression initiates senescence by activating PI3K/AKT/mTOR [194]. Several senescence mediators have been reported, such as SIRT1, SIRT3, SIRT6, senescence-associated secretory phenotype (SASP), and p21^WAF1^. These senescence mediators respond with a distinct modulation by PI3K/AKT/mTOR. Several examples of senescence-modulating effects of PI3K/AKT/mTOR were described as follows.

SIRT1, SIRT3, and SIRT6, the isoforms of the sirtuin family, exhibit modulating functions for AKT activation (Table 2) [167]. SIRT1 deacetylates AKT to promote AKT-phosphatidylinositol 3,4,5 triphosphate (PIP3) binding to phosphorylate and activating AKT. In contrast, SIRT3 inhibits mitochondrial ROS generation to suppress AKT activation. SIRT6 forms a complex with c-Jun and causes chromatin condensation to suppress AKT signaling [167].

Additionally, chronic activation of PI3K/AKT/mTORC1 signaling triggers the oncogene-induced senescence-like phenotype, namely AKT-induced senescence (Table 2) [168]. AKT promotes SASP, the secretion of interleukin (IL)-1*α*, IL-1*β*, IL-6, and IL-8, inducing senescence [168]. Moreover, p53-dependent SASP is suppressed by AKT inhibition [195]. Therefore, AKT can induce senescence. Additionally, p21^WAF1/CIP1^ is a senescence initiator [196]. AKT up-regulates p21 expression [169]. Accordingly, AKT induces senescence-like cell growth arrest associated with p21 (Table 2).

Cells may show premature senescence in response to oncogene activation, DNA damage- or ROS-causing agents [197]. The PI3K/AKT/mTOR pathway can regulate senescence and prolong the life span of human primary fibroblasts [197]. Dysfunction of phosphatase and tensin homolog (PTEN), a negative modulator of AKT, causes cellular senescence [197]. UV irradiation promotes AKT/mTOR activation to trigger senescence [198,199,200,201]. Overexpression of myristoylated AKT induces oncogene-induced senescence in primary cultured human endothelial cells [169]. PTEN dysfunction promotes oncogene-induced senescence in primary murine fibroblasts [202]. Therefore, PI3K/AKT/mTOR is essential to oncogene-induced senescence [203].

Moreover, transforming growth factor-β-activated kinase 1 (TAK1) shows the senescence function with persistent SASP expression by activating p38 and PI3K/AKT/mTOR in human stromal cells [204]. Progesterone induces senescence and inhibits glycolytic metabolism of glioblastoma by inactivating EGFR/PI3K/AKT/mTOR [205]. PI3K/AKT/mTOR is essential to induce senescence. Membrane metallo-endopeptidase (MME) is the downstream effector of PI3K to induce senescence [206]. Cervical cancers show low alpha2A-adrenergic receptor (ADRA2A) levels and poor prognoses [207]. Overexpressing ADRA2A causes senescence (β-galactosidase staining) in cervical cancer cells by down-regulating PI3K/AKT/mTOR, which is reverted by ADRA2A knockdown.

### 3.9. Migration and AKT

AKT also modulates cell migration (Figure 1). E2F2, a member of the E2F transcription factor family, is overexpressed in gastric cancer with poor overall survival. E2F2 overexpression in gastric cancer cells improves migration and invasion by down-regulating PI3K/AKT/mTOR-mediated autophagy [208].

Several mediators of migration have been reported, such as matrix metalloproteinase 2 (MMP2), MMP9, and EMT signaling (Table 2). These migration mediators exhibit distinct modulation by PI3K/AKT/mTOR. Several examples of migration-modulating effects of PI3K/AKT/mTOR were earlier reported. AKT indicates an interaction relationship with MMP2 and MMP9 to modulate EMT [2,12]. Several examples demonstrate their interaction connecting to migration. Overexpression of Rab11a, a Rab GTPase, improves MMP2 expression and PI3K/AKT activation in promoting migration of liver cancer cells, which is suppressed by AKT inhibitor [209]. This suggests that AKT can activate MMP2 to improve the migration of cancer cells. PI3K/AKT can up-regulate MMP9 expression in limbal epithelial cells [210]. MMP9 activates the AKT/PI3K to trigger the EMT process, such as CDH1 down-regulation and CDH2 up-regulation, enhancing the proliferation and invasion of Wilms’ tumor-derived cells [170]. Moreover, AKT activation also regulates other EMT-related signaling proteins, such as VIM and snail (SNAI1) [171]. Therefore, AKT, MMP2, MMP9, and EMT-related signaling cooperatively modulate cell migration (Table 2).

Moreover, ADRA2A overexpression induces antiproliferation, apoptosis, and anti-migration/invasion in cervical cancer cells, accompanied by suppressing PI3K/AKT/mTOR [207]. ADRA2A knockdown reversed these changes. Pleckstrin homology such as domain family A member 2 (PHLDA2) knockdown inhibits proliferation and PI3K, promotes apoptosis and autophagy, and blocks EMT by PI3K/AKT/mTOR signaling in colon cancer cells [211].

### 3.10. Cell-Cycle Progression and AKT

The involvement of AKT-modulating AMPK, SIRT1, and MAPK in several functions was mentioned in Table 3. Notably, AMPK, SIRT1, and MAPK can regulate cell-cycle progression. The detailed regulation of the cell cycle was discussed in Section 2.10.

## 4. AKT Effectors Modulate Cell Functions

As described above, AKT connects to AMPK, SIRT1, and MAPK to regulate cell functions. This network (AMPK-SIRT1-MAPK) is summarized to connect AKT effectors (FOXO, c-Myc, mTORC1/2, S6K1/2, SREBP1, 4EBP1, HIF, and GSK3) (Figure 1, Table 3). Several reports support this rationale, as summarized in Table 3.

As for AMPK/AKT effectors (Table 3), AMPK activates FOXO by phosphorylation [212]. 4-O-Methyl-ascochlorin up-regulates AMPK and down-regulates c-Myc of leukemia cells. Similarly, 5-aminoimidazole-4-carboxamide ribonucleotide (AICAR), an AMPK activator, down-regulates c-Myc expression [213]. AMPK shows reciprocal regulation to mTORC1. AMPK inhibits mTORC1 to suppress cell proliferation, while mTORC1 inhibits AMPK to enhance cell proliferation under nutrient stress [214]. In contrast, AMPK activates mTORC2 to enhance cell survival under nutrient stress [215]. AMPK dephosphorylates to inactivate mTOR and its downstream S6K1 and 4EBP1 [216]. AMPK is validated as upstream of SREBP. AMPK phosphorylates SREBP-1c to inhibit proteolytic cleavage SREBP-1c [217]. Hypoxia-inducible factor 1 subunit α (HIF-1α; HIF1A) and AMPK show the interplay regulating cellular hypoxia adaptation [218]. Moreover, AKT cooperating with GSK3 inactivates AMPK signaling [13]. Accordingly, AMPK can connect AKT effectors to regulate cell functions (Figure 1).

For SIRT1/AKT effectors (Table 3), oxidative stress induces the SIRT1-FOXO3 complex formation to improve FOXO3 deacetylation by SIRT1 [235]. FOXO1 up-regulates SIRT1 expression [219]. c-Myc up-regulates SIRT1 mRNA and protein expression [220]. SIRT1 is the upstream inhibitor of mTORC1 [221]. Inhibition of SIRT1 by nicotinamide activates mTORC1. SIRT1 defected mice exhibit mTORC2/AKT inactivation [222]. Overexpression of SIRT1 activates S6K1 signaling to suppress the senescence of fibroblasts [223]. SREBP-1c acetylation enhances lipogenesis in obese mice. SIRT1 deacetylates SREBP-1c and can treat lipid metabolism disorders [224]. SIRT1 knockout causes 4EBP1 phosphorylation [221]. Hypoxia inhibits SIRT1 expression to activate HIF1A [225]. Moreover, SIRT1 can inactivate GSK3, whereas GSK activation suppresses SIRT1-inducing effects [226]. Accordingly, SIRT1 can connect AKT effectors to regulate cell functions (Figure 1).

For MAPK/AKT effectors (Table 3), down-regulation of PI3K/AKT and MAPK (ERK) activates FOXO and induces apoptosis of pancreatic cancer cells [227]. AKT-phosphorylated FOXO1 binds to the IQ Motif Containing GTPase Activating Protein 1 (IQGAP1) to inactivate ERK [228]. MAPK up-regulation blocks GSK3-β-dependent c-Myc degradation of asparagine-restricted melanoma cells. Inhibition of MAPK-c-Myc signaling exhibits antiproliferation of melanoma cells [229]. Inhibition of mTORC1 activates MAPK in prostate cancer cells [230]. mTORC2 inactivates p38 by stabilization of p38 phosphatase (DUSP10) [231]. SIRT1 up-regulates ERK to promote the proliferation of senescent fibroblasts [223]. ERK1/2 can phosphorylate SREBP-1a [232]. p38 phosphorylates to up-regulate 4EBP1 expression [233]. MAPK activates HIF involving p300/CREB binding protein (CBP) [36]. Moreover, GSK3 is a suppressor of ERK1/2 [234]. Accordingly, MAPK can connect AKT effectors to regulate cell functions (Figure 1).

Detailed information on AKT effectors affecting cell functions is mentioned (Section 4.1, Section 4.2, Section 4.3, Section 4.4, Section 4.5, Section 4.6, Section 4.7, Section 4.8, Section 4.9 and Section 4.10) (Table 4).

### 4.1. Apoptosis and AKT Effectors

The connection between AKT effectors and apoptosis is widely investigated in several studies (Table 4). For example, FOXO up-regulation triggers apoptosis of several cancer cells [236]. AKT down-regulation causes FOXO3a-mediated apoptosis of prostate cancer [237]. c-Myc can modulate cancer cell proliferation and apoptosis [253]. Glutaminolysis activates mTORC1 to suppress autophagy and trigger apoptosis of cancer cells [266]. Moreover, a high concentration of mTORC1 inhibitor (Everolimus) [332] triggers extrinsic apoptosis of colon cancer cells associated with inhibiting 4EBP1 [267]. Similarly, mTORC2 knockdown enhances apoptosis of breast cancer cells [268].

S6K1 and S6K2 have distinct functions in cancer cells [333]. S6K1 knockdown activates AKT to suppress apoptosis, while S6K2 knockdown inactivates AKT to promote apoptosis [281]. Additionally, inhibition of S6K1 by rosmarinic acid methyl ester (RAME) triggers apoptosis of cervical cancer cells [282]. It indicates that S6K1 and S6K2 can differentially regulate apoptosis in cancer cells. SREBP1 knockdown causes antiproliferation and triggers apoptosis in pancreatic cancer cells [292]. In contrast, SREBP1 overexpression induced by high glucose enhances proliferation and inhibits apoptosis of pancreatic cancer cells [293]. It indicates that SREBP1 can inhibit apoptosis in cancer cells.

Additionally, 4EBP1 knockdown suppresses enzastaurin-triggered apoptosis in cancer cells [302]. Torin 1, a mTORC1/2 inhibitor, activates extrinsic apoptosis of colon cancer cells by dephosphorylating 4EBP1 [267]. It indicates that 4EBP1 can inhibit apoptosis in cancer cells. HIF1A up-regulates miR-21 [312] to suppress apoptosis of pancreatic cancer cells [313]. GSK3 consists of two isoforms (α and β). GSK3 can regulate apoptosis [323]. Leucine zipper tumor suppressor 1 (LZTS1) inhibits apoptosis of pancreatic cancer cells by inactivating AKT/GSK-3 [324].

### 4.2. Autophagy and AKT Effectors

The connection between AKT effectors and autophagy is widely investigated in several studies (Table 4). The relationship between FOXO and autophagy in cancer cells was reviewed [238]. For example, SIRT1 improves FOXO1 deacetylation for inducing autophagy of cardiac myocytes by overexpressing RAB7A, member RAS oncogene family (RAB7A) for autophagosome-lysosome fusion [240]. Moreover, FOXO3 also shows autophagy-inducible function during muscle atrophy by enhancing several autophagy genes [239,334].

Myc knockdown impairs autophagosome formation to block autophagy by dephosphorylating JNK1 [254]. Cancerous inhibitor of [protein phosphatase 2A] PP2A (CIP2A) is an allosteric inhibitor of the mTORC1/PP2A complex, inhibiting mTORC1-dependent autophagy [270]. mTORC1 phosphorylates ULK1 to inhibit the early stage of autophagy [269]. Additionally, mTORC2 indirectly blocks autophagy by activating mTORC1 and AKT [271].

Moreover, rosmarinic acid methyl ester (RAME) inhibits S6K1 by triggering autophagy in cervical cancer cells [282]. High glucose induces SREBP1 overexpression to suppress the autophagy of pancreatic cancer cells [293]. YXM110, a synthesized phenanthroquinolizidine, down-regulates 4EBP1, associated with autophagy of cancer cells [303]. 4EBP1 overexpression suppresses bortezomib-promoted autophagy of liver cancer cells [304]. It indicates that 4EBP1 can inhibit autophagy in cancer cells. HIF1A promotes the autophagy of pancreatic cancer [314]. GSK3 can regulate autophagy by modulating autophagy inducers such as ULK1 [325].

### 4.3. ER Stress and AKT Effectors

Several studies have widely investigated the connection between AKT effectors and ER stress (Table 4). For example, FOXO can interplay with ER stress in cancer [335]. FOXO1 inhibitor (AS1842856) triggers ER stress in unstimulated T cells [241]. FOXO inhibits nutrient restriction-induced ER stress of Tsc1 mutant cells [242]. It indicates that FOXO inhibits ER stress. Additionally, c-Myc causes ER stress by activating IRE1α-X-box binding protein 1 (XBP1) signaling [255]. c-Myc induces an adaptive ER stress in mice with liver tumor burden. In contrast, c-Myc knockdown down-regulates GRP78, ATF4, and CHOP [256]. It indicates that c-Myc induces ER stress.

Torin 1, a mTORC1/2 inhibitor, activates ER stress of colon cancer cells [267]. It indicates that mTORC1/2 inhibits ER stress. S6K1 deficiency inhibits palmitic acid-induced ER stress in immortalized mouse hepatocytes [283], suggesting that S6K1 induces ER stress. SREBP1 knockdown triggers ER stress in glioblastoma cells [294]. In contrast, ER stress may activate SREBP1 of liver cancer cells [295]. It indicates that SREBP1 and ER stress exhibit reciprocal regulation. Torin 1 also inhibits 4EBP1 to induce ER stress in colon cancer cells [267]. It demonstrates that 4EBP1 inhibits ER stress. Thapsigargin triggers ER stress by up-regulating HIF1A and HIF2α expression [315]. GSK3 activation is essential for ER stress-triggered pancreatic β-cell apoptosis [326].

### 4.4. Mitochondrial Morphogenesis and AKT Effectors

Several studies have widely investigated the connection between AKT effectors and mitochondrial fission/fusion (Table 4). For example, FOXO1 promotes MFN1 and MFN2 (fusion) expression but inhibits DRP1 and FIS1 (fission) expression, resulting in enlarged mitochondria of hepatocytes [243]. FOXO3 overexpression inhibits mitochondrial fission of cardiomyocytes by down-regulating MIEF2 [243]. However, FOXO3 may cause mitochondrial fission by activating Drp1 in phenylephrine-stimulated adult cardiomyocytes. It indicates that FOXO may regulate mitochondrial fission and fusion.

Moreover, c-Myc triggers mitochondrial fission, up-regulates oxidative signaling, and promotes ROS generation [257]. mTORC1 plays a central role in regulating the interchange between mitochondrial fission and fusion [272]. mTORC1 inhibits mitochondrial fusion but enhances mitochondrial fission. Additionally, mTORC2 enhances AKT phosphorylation (Ser 473) and provides indirect regulation to several AKT downstream signaling [272,327].

mTORC1 activates S6K to inhibit mitochondrial fusion [272]. S6K1 knockout enhances mitochondrial fission of mouse embryonic fibroblasts [284]. It indicates that S6K inhibits mitochondrial fusion. Activating mitochondrial fission deacetylates SREBP1 while inhibiting mitochondrial fission acetylates SREBP1 in liver cancer cells [296]. Furthermore, mTORC1 suppresses 4EBP1-inactivated mitochondrial fission, enhancing fission [272]. mTORC1 down-regulation induces mitochondrial fusion by 4EBP-dependent translational inhibition of mitochondrial fission process protein 1 (MTFP1) [305]. It indicates that 4EBP1 inhibits mitochondrial fission. HIF1A phosphorylates DRP1 to induce mitochondrial fission [316,317]. In response to oxidative stress, GSK3β-mediated DRP1 phosphorylation triggers mitochondrial elongation of cervical cancer cells [327].

### 4.5. Ferroptosis and AKT Effectors

Several studies have widely investigated the connection between other AKT effectors and ferroptosis (Table 4). For example, FOXO is co-expressed in ferroptosis-related lncRNA signaling [244]. c-Myc can interact with cysteine dioxygenase 1 (CDO1) [258] and enhances ferroptosis [259]. mTORC1 is known as a central ferroptosis modulator. mTORC1 up-regulates SREBP1 to improve cancer cell tolerance to ferroptosis [273]. In contrast, the mTORC1 inhibitor suppresses GPX4 expression to enhance cell sensitivity to ferroptosis [274]. It indicates that mTORC1 inhibits ferroptosis. S6K2-depleted cells activate SREBP1 and induce ferroptosis, as demonstrated by up-regulating ferroptosis marker prostaglandin-endoperoxide synthase 2 (PTGS2) [285]. Everolimus and RSL3/erastin show cooperative antiproliferation and ferroptosis in renal cancer cells by suppressing mTOR-4EBP1 signaling [306]. Hypoxia-induced HIF1A suppresses ferroptosis in metastasis of gastric cancer [318]. GSK-3β is a positive effector of ferroptosis. Down-regulation of GSK-3β enhances ferroptosis resistance [328].

### 4.6. Necroptosis and AKT Effectors

Several studies have widely investigated the connection between other AKT effectors and mitochondrial fission/fusion (Table 4). For example, platycodin D promotes necroptosis of prostate cancer cells by up-regulating FOXO3a expression and phosphorylating MLKL [245]. MYC shows an antinecroptotic function by inhibiting RIPK1-RIPK3 complex formation [260]. Additionally, inhibition of mTORC1 but not mTORC2 suppresses necroptosis of cardiomyocytes by RIP1 inhibition-mediated transcriptional factor EB (TFEB) activation [275]. Necroptosis is associated with hyperphosphorylation of FoxO, GSK3, and S6K1/2 [187]. SREBP1 up-regulates lipid production to inhibit cell proliferation during necroptosis, reverted by inactivating SREBP1 [297]. mTOR inhibitors suppress tumor necrosis factor (TNF)-induced necroptosis of fibrosarcoma cells by down-regulating mTOR and 4EBP1 [307]. Oxygen-glucose deprivation causes necroptosis, accompanied by up-regulating HIF1A [319]. GSK3β is a crucial modulator of 1,4-naphthoquinone (DMNQ)-induced necroptosis of glioma cells [329].

### 4.7. DNA Damage Response and AKT Effectors

Several studies have widely investigated the connection between AKT effectors and DNA damage response (Table 4). For example, *N*-methyl-*N*′-nitro-*N*-nitrosoguanidine induces DNA damage in lung cancer cells by enhancing the nuclear import of FOXO1, which regulates DNA damage repair [246]. Moreover, FOXO3a suppresses genomic instability by inhibiting DNA double-strand break-induced mutations [247].

c-Myc evokes oxidative stress to trigger DNA damage [261]. Bcl2 up-regulates c-Myc expression to inactivate apurinic/apyrimidinic endonuclease (APE1) for suppressing DNA repair [262]. DNA damage activates mTORC1 [276] and mTORC2 [277]. For example, X-ray-induced DNA damage phosphorylates mTORC2 depending on DNA-PK [277]. DNA damage activates S6K1 and interacts with MDM2 to regulate DNA damage response [286]. Moreover, S6K1 also shows DNA repair function by phosphorylating cyclin-dependent kinase 1 (CDK1) and MutS homolog 6 (MSH6) [287].

Moreover, the lipogenic liver X receptor (LXR)-SREBP1 axis can regulate DNA repair, such as in the up-regulation of DNA repair gene polynucleotide kinase/phosphatase (PNKP), which is down-regulated in several cancer cells [298]. mTOR inhibition suppresses DNA replication and repair via 4E-BP1 activation, reverted by 4EBP1 depletion [308]. DNA damage suppresses HIF1A expression [320]. GSK3 can regulate DNA repair [323]. Inhibition of GSK3A blocks DNA repair in response to DNA-damaging drugs [330].

### 4.8. Senescence and AKT Effectors

The connection between AKT effectors and senescence is widely investigated in several studies (Table 4). For example, FOXO3a down-regulation improves the senescence of dermal fibroblasts [248]. FOXO1 is a negative modulator of senescence in T cells [249]. C-Myc enhances the oncogene-induced senescence of tumor cells [263].

mTORC1 improves preosteoblast senescence [278]. mTORC2 induces senescence of endothelial cells by down-regulating NRF2 [279]. S6K1/2 deletion suppresses the senescence of mouse embryonic fibroblasts [289]. Senescent endothelial cells show up-regulation of S6K1 [288]. SREBP1 induces lipogenesis and senescence of fibroblasts [299]. Moreover, discodermolide resistance is generated by enhancing senescence, associated with the down-regulation of 4EBP1 [309]. HIF1A inhibition promotes senescence by up-regulating p21 and down-regulating telomerase reverse transcriptase (TERT) [321]. Inhibition of GSK3 triggers the senescence of normal hepatocytes [331].

### 4.9. Migration and AKT Effectors

The connection between AKT effector and migration is widely investigated in several studies (Table 4). For example, FOXO is a mediator for regulating EMT expression [250]. FOXO3a suppresses the invasion of mammary adenocarcinoma cells and blocks TGF-β1-promoted EMT in mouse mammary epithelial cells [251]. c-Myc knockdown inhibits cell migration of liver cancer cells [264]. mTORC1 inhibition suppresses hypoxia-induced migration of keratinocytes [280]. Knockdown of mTORC2 prevents cell migration [268].

p85 isoform of S6K1 enhances migration and tumor growth [290]. SREBP1 enhances the migration of breast cancer cells [300]. 4EBP1 down-regulation promotes EMT and migration of colon cancer cells [310]. HIF1A enhances EMT and migration in pancreatic cancer stem cells [314]. GSK3 can regulate invasion and metastasis [323].

### 4.10. Cell-Cycle Progression and AKT Effectors

PI3K inhibitor ETP-45658 causes cell-cycle arrest of breast cancer cells depending on FOXO [252]. S6K2 inhibitor induces G1 arrest to inhibit the proliferation of melanoma cells [291]. PI3K/mTOR inhibitor PQR309 inhibits the PI3K/AKT/mTOR/c-Myc axis and causes G1 arrest of endometrial cancer cells [265]. Mature SREBP1 is regulated by hyperphosphorylation for G2/M arrested cervical cancer cells [301]. Prothioconazole causes G1 arrest of extravillous trophoblast cells by enhancing 4EBP1 expression [311]. HIF1A promotes G1 arrest of colon cancer cells by down-regulating c-Myc-activated gene expressions [322]. GSK3 can regulate cell-cycle progression [323].

## 5. Natural Products Regulate Oxidative Stress-Modulated Cell Functions

Natural products are commonly rich in antioxidants. Antioxidants may have distinct functions for preventing [336,337] and promoting oxidative stress [338,339]. This dual function of antioxidants may depend on concentration effects. Low concentrations of antioxidants are reported to down-regulate oxidative stress, while high concentrations show harmful effects [340,341,342]. In general, oxidative stress is generated when the cellular antioxidants and prooxidants lose balance. Natural products may act as oxidative stress inducers in certain environments to modulate redox homeostasis, triggering cell death [30]. Several oxidative stress-inducible natural products that modulate several cell functions were provided (Section 5.1, Section 5.2, Section 5.3, Section 5.4, Section 5.5, Section 5.6, Section 5.7, Section 5.8, Section 5.9 and Section 5.10) (Table 5).

### 5.1. Natural Products Targeting Apoptosis through Oxidative Stress

There are several oxidative stress-inducing natural products that modulate apoptosis (Table 5). Cryptocarya-derived cryptocaryone triggers oxidative stress and promotes apoptosis of oral cancer cells [343]. Several *Rubus fairholmianus*-derived compounds stimulate oxidative stress and cause apoptosis of breast cancer cells [30]. Sanguinarine, a *Sanguinaria canadensis*-derived compound, is a bloodroot plant-derived natural alkaloid with antifungal effects [363]. The repurposing function of sanguinarine has been applied to trigger apoptosis for anticancer treatment. For example, sanguinarine inhibits thioredoxin reductase to induce oxidative stress and trigger apoptosis of cancer cells [344].

### 5.2. Natural Products Targeting Autophagy through Oxidative Stress

Several oxidative stress-inducible natural products are known that modulate autophagy (Table 5). Isoaaptamine, a marine sponge-derived compound, induces apoptosis and autophagy of breast cancer cells by generating oxidative stress [345]. Neferine, a *Nelumbo nucifera*-derived dibenzylisoquinoline alkaloid, induces the generation of ROS to trigger the autophagy of lung cancer cells [346]. Piperlongumine, a *Piper longum*-derived compound, induces autophagy and cell death of osteosarcoma cells, reverted by *N*-acetylcysteine, suggesting that piperlongumine induces autophagy depending on oxidative stress [347].

### 5.3. Natural Products Targeting ER Stress through Oxidative Stress

Several anticancer drugs with oxidative stress-modulating ability can regulate ER stress (Table 5) [47]. For example, sarsasapogenin, an *Anemarrhena asphodeloides*-derived compound, promotes oxidative stress and ER stress in cervical cancer cells, reverted by *N*-acetylcysteine [348].

### 5.4. Natural Products Targeting Mitochondrial Morphogenesis through Oxidative Stress

There are several oxidative stress-inducible natural products that modulate mitochondrial morphogenesis (Table 5). Ferric ammonium citrate (FAC) induces ROS generation, iron overload, and apoptosis [349]. Still, FAC inhibits mitochondrial fusion/fission of bone marrow mesenchymal stem cells (BMSCs), which is reverted by a *Herba epimedii*-derived flavonoid glucoside icariin [349]. The T-2 Toxin, a fungal secondary metabolite, enhances oxidative stress, abnormal mitochondrial fission/fusion, and apoptosis in liver cells [350]. Oxidative stress promotes DRP-1 translocation from cytoplasm to mitochondria, leading to mitochondrial fission [364], reverted by the natural disaccharide trehalose [351].

### 5.5. Natural Products Targeting Ferroptosis through Oxidative Stress

Several oxidative stress-inducible natural products modulate ferroptosis (Table 5). Apigenin, a fruit and vegetable-derived flavonoid, suppresses myeloperoxidase-induced oxidative stress to block ferroptosis of neuron cells [352]. Tagitinin C, a *Tithonia diversifolia*-derived lactone, triggers ferroptosis by inducing glutathione (GSH) depletion and lipid peroxidation [353].

### 5.6. Natural Products Targeting Necroptosis through Oxidative Stress

Several oxidative stress-inducing natural products modulate necroptosis (Table 5). Curcumol, a *Curcuma wenyujin*-derived sesquiterpenoid, induces mitochondrial superoxide and JNK1/2 activation, associated with RIPK1/RIPK3-regulating necroptosis in hepatic stellate cells, which are suppressed by ROS scavenger, *N*-acetylcysteine and JNK1/2 inhibitor (SP600125). Accordingly, curcumol induces necroptosis in a ROS and JNK-dependent manner [354]. Shikonin, a *Lithospermum euchroma*-derived compound, induces ROS generation and RIPK1/RIPK3/MLKL expression in nasopharyngeal cancer cells, leading to necroptosis, which is suppressed by necrostatin-1 [355].

### 5.7. Natural Products Targeting DNA Damage Response through Oxidative Stress

Several oxidative stress-inducible natural products that enhance DNA damage response were provided as follows (Table 5). Cryptocaryone causes oxidative stress-dependent DNA damage in oral cancer cells [343]. Sinuleptolide, a soft corals-derived natural product, induces antiproliferation and oxidative stress in oral cancer cells, accompanied by DNA damage [356].

### 5.8. Natural Products Targeting Senescence through Oxidative Stress

Several oxidative stress-inducible natural products that modulate senescence were provided as following (Table 5). Apigenin inhibits oxidative stress-triggered senescence of lung fibroblasts [357]. Gingerenone A, a ginger-derived compound, inhibits proliferation and triggers oxidative stress and senescence of breast cancer cells, reverted by *N*-acetylcysteine [358].

### 5.9. Natural Products Targeting Migration through Oxidative Stress

Several oxidative stress-inducible natural products modulate migration (Table 5). Salinomycin promotes oxidative stress generation to decrease the proliferation and migration of prostate cancer cells [359]. Withaferin A blocks migration and invasion and induces oxidative stress of oral cancer cells, reverted by *N*-acetylcysteine [360].

### 5.10. Natural Products Targeting Cell-Cycle Progression through Oxidative Stress

Lycopene, a plant-derived carotenoid, inhibits 7-ketocholesterol-triggered oxidative stress and G1 arrest of macrophages [361]. Gracillin, a *Dioscorea nipponica*-derived steroidal saponin, induces G1 arrest of leukemic cells by oxidative stress [362].

## 6. Natural Products Regulate AKT-Modulated Cell Functions

Several AKT-modulated natural products modulate diverse cell functions (Table 6) (Section 6.1, Section 6.2, Section 6.3, Section 6.4, Section 6.5, Section 6.6, Section 6.7, Section 6.8, Section 6.9 and Section 6.10).

### 6.1. Natural Products Targeting Apoptosis through AKT

Some natural products protect from apoptosis, but others induce it. Several reports investigated the apoptosis-protecting effects of natural products involving AKT (Table 6). For example, the suppression of oxidative stress and inflammation. Ginsenoside Rd, a *Panax japonicus*-derived natural product, enhances neural cell survival by reducing oxidative stress, increasing antioxidant expression, activating PI3K/AKT and ERK 1/2 pathways, and reducing apoptosis [377]. Troxerutin, a rutin-derived semi-synthetic bioflavonoid, decreases ROS and apoptosis by up-regulating antioxidant enzymes and translocating NRF2 [393]. Crocin, a crocus flower-derived carotenoid, suppresses retinal ischemia/reperfusion injury-triggered apoptosis of ganglion cells by activating AKT [369].

Troxerutin also inactivates acetylcholinesterase and activates PI3K/AKT in Alzheimer’s disease models (Table 6) [393]. Moreover, fucoxanthin, a marine seaweed-derived carotenoid, suppresses H_2_O_2_-triggered ROS generation and apoptosis by down-regulating H_2_O_2_-activated AKT and ERK expression [376].

Several reports investigated the apoptosis-promoting effects of natural products involving AKT (Table 6). For example, CXC195, a tetramethylpyrazine with antioxidant activity, triggers apoptosis in liver cancer cells by suppressing the PI3K/AKT/mTOR pathway [370]. Emodin, a natural anthraquinone, induces antiproliferation and apoptosis of leukemia cells by up-regulating phosphatase and tensin homolog (PETN) and inhibiting PI3K/AKTmRNA expression [373]. Acetyl-lupeolic acid, a *Boswellia carterii*-derived compound, triggers apoptosis in prostate cancer cells by suppressing AKT/mTOR [365].

Moreover, 4β-hydroxywithanolide E triggers apoptosis of breast cancer cells by suppressing PI3K/AKT (Table 6) [380]. YVPGP, an *Anthopleura anjunae*-derived peptide, shows antiproliferation against prostate cancer cells by inactivating PI3K/AKT/mTOR [395]. Piperlongumine, a *Piper longum*-derived compound, induces antiproliferation and apoptosis of colon cancer cells by suppressing Ras/PI3K/AKT/mTOR [388]. Trametenolic acid B, a *Trametes lactinea*-derived compound, shows neuroprotective and antiapoptosis ability in oxygen-glucose deprivation/reoxygenation-damaged SH-SY5Y cells by up-regulating PI3K/AKT/mTOR [392]. Timosaponin TAIII, a rhizome of *Anemarrhena asphodeloides*-derived compound, induces apoptosis in lung cancer cells by down-regulating PI3K/AKT/mTOR and Ras/Raf/MEK/ERK pathways [391]. Diallyl trisulfide, a bioactive compound from processed garlic, shows anticancer effects [444]. Diallyl trisulfide induces apoptosis of prostate cancer cells by inhibiting AKT phosphorylation and inducing inactivation [372]. Fisetin, a bioactive flavonoid derived from strawberry, apple, and onion, triggers apoptosis of osteosarcoma cells involving MAPK and PI3K/AKT signaling [375]. Myricetin, the onions and grape-derived flavonoid, induces apoptosis of umbilical vascular endothelial cells by inhibiting AKT [383]. Grape proanthocyanidin induces antiproliferation and apoptosis of pancreatic cancer cells by inactivating AKT [389].

Lupiwighteone, a *Cadophora gregata*-derived compound, triggers apoptosis in breast cancer cells by inactivating PI3K/AKT/mTOR (Table 6) [382]. Ginsenoside Rh2, a ginseng-derived compound, shows antiproliferation and apoptosis of osteosarcoma by activating mitogen-activated protein kinase (MAPK) and inactivating PI3K/AKT/mTOR and nuclear factor-κB (NF-κB) [378]. 9-Demethylmucroniferanine A, Tibetan Medicine *Corydalis hendersonii* Hemsl-derived bioactive compound, triggers apoptosis in gastric cancer cells by suppressing PI3K/AKT/mTOR and topoisomerase I [371]. Uvaol, a natural triterpenoid, down-regulates anti-apoptosis gene Bcl-2 and up-regulates pro-apoptotic protein Bax, accompanied by inactivating AKT/PI3K signaling in liver cancer HepG2 cells [394].

Additionally, asperpyrone A, an Aspergillus-derived compound, induces antiproliferation and apoptosis of pancreatic cancer cells by down-regulating ROS-mediated PI3K/AKT (Table 6) [366]. Piperine, a natural alkaloid, inhibits the migration of prostate cancer cells via suppressing AKT/mTOR/MMP9 signaling [387]. Krukovine, an *Abuta grandifolia*-derived alkaloid, shows antiproliferation of lung cancer cells by inactivating PI3K/AKT/mTOR/p70s6k and down-regulating ERK [381]. NAP, a *Nereis virens*-derived serine protease, shows antiproliferation and apoptosis in lung cancer cells by down-regulating the PI3K/AKT/mTOR pathway [384].

Transforming growth factor (TGF)-β1 overexpression activates PI3K/AKT/mTOR in breast cancer and increases the resistance potential to chemotherapy (Table 6) [445]. Applying PI3K/AKT/mTOR axis inhibitors such as NVP-BEZ235 is a potential therapy for breast cancer [385]. Caffeic acid phenethyl ester (CAPE), a propolis-derived bioactive compound, shows antiproliferation to cancer cells by down-regulating PI3K/AKT/mTOR [368]. Combined NVP-BEZ235 and CAPE synergistically induce antiproliferation and apoptosis [445].

Ferulin C, a *Ferula ferulaeoides*-derived natural product, induces apoptosis and autophagy by down-regulating AKT-mTOR signaling (Table 6) [374]. A cruciferous vegetable-derived natural compound, sulforaphane, can inhibit AKT/mTOR [446]. Combined with everolimus, it can reduce the drug resistance of bladder cancer cells [447]. Grincamycin B, a *Streptomyces lusitanus*-derived natural product, inhibits glioma cell proliferation and invasion by suppressing PI3K/AKT and alkaline phosphatase (PHOA) signaling [379]. Bavachinin, an active *Proralea corylifolia*-derived flavanone, can activate MAPK and AKT signaling in lung cancer cells [367].

### 6.2. Natural Products Targeting Autophagy through AKT

Natural products may exhibit autophagy-modulating effects involving AKT (Table 6). For example, tanshinone IIA, a *Salvia miltiorrhiza* Bunge-derived bioactive compound, suppresses proliferation and induces autophagy of breast cancer cells by inactivating PI3K/AKT/mTOR signaling [411]. Patulin, a *Penicillium*-derived compound, promotes ROS generation and autophagy of liver cancer cells by inactivating AKT1/mTOR, reverted by *N*-acetylcysteine [408]. Fisetin exhibits a dual function for inhibiting PI3K/AKT and mTOR and promotes cytotoxic autophagy in prostate cancer cells [403]. Allicin, a bioactive compound from crushed garlic, triggers autophagy of liver cancer cells by inactivating AKT [397]. Crocin promotes autophagy and causes cell death of cervical cancer cells by activating AKT [400].

Notably, some natural products exhibit bifunctional effects for modulating autophagy and apoptosis (Table 6). For example, spicatoside A, a *Liriope platyphylla*-derived steroidal saponin, induces autophagy at 24 h by inactivating PI3K/AKT/mTOR, but it induces apoptosis at long-term exposure [409]. Grape seed procyanidin B2 enhances apoptosis and autophagy of colorectal cancer cells by inactivating AKT [390]. 6-Gingerol, a ginger-derived compound, inhibits hydrogen peroxide-promoted apoptosis of human umbilical vein endothelial cells (HUVECs) by inactivating PI3K/AKT/mTOR and inducing autophagy [405]. Additionally, spicatoside A, a *Liriope platyphylla*-derived compound, induces autophagy and apoptosis of colon cancer cells by down-regulating PI3K/AKT/mTOR [409]. Streptomyces sp metabolite(s) triggers apoptosis and autophagy of cervical cancer cells by down-regulating mTOR [410].

Similarly, chaetocochin J, a natural alkaloid, promotes apoptosis and autophagy of colon cancer cells by inactivating PI3K/AKT/mTOR and activating AMPK (Table 6) [398]. Falcarindiol, an *Oenanthe javanica*-derived compound, induces apoptosis and autophagy of oral cancer cells by inactivating PI3K/AKT/mTOR/p70S6K [402]. CLE-10, a *Carpesium abrotanoides*-derived compound, induces apoptosis and autophagy of breast cancer cells by inactivating PI3K/AKT/mTOR [399]. Geraniol up-regulates KEAP1/NRF2/heme oxygenase-1 (HO-1), activates antioxidant expressions, and promotes PI3K/AKT/mTOR to inhibit myocardial autophagy and apoptosis [404].

Moreover, echinatin, a *Glycyrrhiza uralensis*-derived compound, shows antiproliferation, apoptosis, and autophagy of esophageal cancer cells by inactivating AKT/mTOR (Table 6) [401]. Neferine, liensinine, and isoliensinine, the *Nelumbo nucifera*-derived bisbenzylisoquinoline alkaloids, trigger apoptosis and autophagy of prostate cancer cells by inactivating PI3K/AKT [407]. Ilimaquinone, a marine sponge-derived compound, induces ROS generation, apoptosis, and autophagy by down-regulating AKT and ERK and up-regulating p38 [406].

### 6.3. Natural Products Targeting ER Stress through AKT

Natural products may exhibit ER stress-modulating effects involving AKT (Table 6). For example, ischemia-reperfusion (I/R) injury induces ER stress and inactivates PI3K/AKT/mTOR, which are reverted by ginkgolide B, a *Ginkgo biloba*-derived terpene lactone. Therefore, ginkgolide B shows PI3K/AKT/mTOR activating ability to suppress ER stress and I/R injury [415]. Methamphetamine (METH), a neuron stimulant and addictive drug, causes irreversible pathological changes to neurons, associated with ER stress induction and PI3K/AKT/mTOR inactivation [416]. Aromadendrin, a flavanonol natural product, can inhibit METH-induced autophagy, apoptosis, and ER stress of SH-SY5y cells, accompanied by up-regulating PI3K/AKT/mTOR [412]. Procyanidin B2 suppresses ER stress of endothelial cells involving peroxisome proliferator-activated receptor δ (PPARδ) [417], which activates AKT signaling to cause endothelial dysfunction in diabetic mice [448].

β-Carotene, a vitamin A precursor, inhibits ROS generation, induces antioxidant expression, inhibits apoptosis, autophagy, and ER stress, and activates PI3K/AKT/mTOR [414]. Bleomycin causes pulmonary fibrosis, associated with ER stress and PI3K/AKT expression, recovered by ER stress or PI3K inhibitors [413].

### 6.4. Natural Products Targeting Mitochondrial Morphogenesis through AKT

Natural products may prevent and improve mitochondrial fission (Table 6). For example, icariin suppresses ferric ammonium citrate-inhibited mitochondrial fusion/fission of bone marrow mesenchymal stem cells by activating PI3K/AKT/mTOR and inactivating ERK1/2 and JNK pathways [349]. For comparison, lupeol, a *Bombax ceiba*-derived natural product, triggers mitochondrial fission of renal cancer cells [418]. Moreover, lupeol blocks 12-O-tetradecanoyl-phorbol-13-acetate (TPA)-promoting AKT activation and skin cancer growth in CD-1 mice [419]. Accordingly, the AKT impact on regulating mitochondrial fission of lupeol is warranted for detailed investigation.

Moreover, allicin also suppresses 6-hydroxydopamine-up-regulated FIS1 and DRP1 expressions in pheochromocytoma PC12 cells, which trigger mitochondrial fission [449]. However, the role of AKT in mitochondrial fission-promoting effects of allicin was not reported by this study, and it warrants a detailed investigation in the future.

### 6.5. Natural Products Targeting Ferroptosis through AKT

The ferroptosis impact of natural products involving AKT was rarely investigated. Prostate [450] and lung [184] cancer cells highly express PI3K/AKT. Lung cancer cells inhibit ferroptosis by activating PI3K/AKT/mTOR [184]. In contrast, PI3K and mTOR inactivations induce ferroptosis in cancer cells [273]. Accordingly, it warrants a detailed investigation to identify AKT-modulating natural products in the future (Table 6).

### 6.6. Natural Products Targeting Necroptosis through AKT

Natural products may exhibit necroptosis-modulating effects involving AKT (Table 6). For example, paeoniflorigenone, *Paonia suffruticosa*-derived natural products, shows antiproliferation, apoptosis, and autophagy in head and neck cancer cells by inactivating PI3K/AKT/mTOR/p70S6K signaling [386]. Moreover, paeoniflorigenone-inhibiting PI3K/AKT/mTOR/p70S6K signaling leads to a suppression of necroptosis by inactivating necroptotic proteins (RIP and MLKL) in head and neck cancer cells [386].

### 6.7. Natural Products Targeting DNA Damage Response through AKT

Natural products may exhibit DNA damage response-modulating effects involving AKT (Table 6). For example, cerberin, a cardenolide isolated from the fruit kernel of *Cerbera odollam*, induces antiproliferation, anti-migration, apoptosis, ROS production, and DNA damage in cancer cells, accompanied by inhibiting PI3K/AKT/mTOR signaling [420]. Cucurbitacin-A, a cucurbitaceous plant-derived compound, exhibited antiproliferation, ROS generation, and DNA damage of ovarian cancer cells by inactivating PI3K/AKT/mTOR [422].

Additionally, lanatoside C, a natural antiarrhythmic product derived from *Digitalis lanata*, shows antiproliferation, apoptosis, and DNA damage in cancer cells by inactivating PI3K/AKT/mTOR (Table 6) [423]. Peruvoside, a *Cascabela thevetia*-derived cardiac glycoside, induces antiproliferation and DNA damage and inhibits autophagy in breast, lung, and liver cancer cells, accompanied by PI3K/AKT/mTOR [425]. Strophanthidin, a natural cardiac glycoside, causes apoptosis and DNA damage in several cancer cells (breast, lung, and liver), associated with down-regulating PI3K/AKT/mTOR [427]. Romidepsin, a *Chromobacterium violaceum*-derived natural product, promotes oxidative stress and DNA damage by activating PI3K/AKT/mTOR and MAPK signaling in rhabdomyosarcoma cells [426]

Moreover, paclitaxel induces ROS generation, DNA damage, and apoptosis in lung cancer cells by suppressing EGFR/PI3K/AKT/mTOR (Table 6) [424]. TA25, a *Salvia miltiorrhiza*-derived tanshinone IIA analog, causes ROS generation and DNA damage in lung cancer cells by inactivating PI3K/AKT/mTOR but up-regulating p53 protein [428]. Chalcone is a typical core structure in natural products [421].

### 6.8. Natural Products Targeting Senescence through AKT

Some natural products provide senescence-modulating effects involving AKT (Table 6). For example, coroglaucigenin, a *Calotropis gigantean*-derived compound, shows antiproliferation, autophagy, and senescence in colon cancer cells by dissociating HSP90 with CDK4 and AKT, degrading CDK4, and inactivating AKT [429]. Additionally, bleomycin down-regulates PTEN and activates PI3K/AKT/mTOR to cause senescence in lung cancer cells [430]. PTEN knockdown inhibits autophagy and induces senescence of A549 cells, which is reverted by the mTOR inhibitor. Therefore, PTEN and PI3K/AKT/mTOR axis regulate bleomycin-induced senescence by inhibiting autophagy. Moreover, doxycycline triggers Notch1 activation, apoptosis, and autophagy by suppressing the PI3K/AKT/mTOR axis in osteosarcoma cells [431]. Fisetin reverses adriamycin-induced senescence of vascular endothelial cells by up-regulating PTEN, an AKT inhibitor [432]. Proanthocyanidins suppress interleukin-1β-induced senescence of nucleus pulposus cells by activating AKT [433].

### 6.9. Natural Products Targeting Migration through AKT

Natural products may exhibit migration-modulating effects involving AKT (Table 6). In addition to antiproliferation, vegetable-derived fisetin inhibits the migration and invasion of pancreatic cancer cells by down-regulating PI3K/AKT/mTOR [435]. Moreover, fisetin suppresses metastasis in tumor-bearing mice [451]. Additionally, platycodin D, a *Platycodon grandiflorum*-derived triterpenoid saponin, inhibits breast cancer cell proliferation, migration, and invasion by inhibiting EGFR-mediated AKT and MAPK signaling [437]. Alisol A, a triterpenoid in the *Alismatis* rhizome, promotes autophagy and inhibits PI3K/AKT/mTOR in breast cancer cells [396]. Alisol A inhibits migration and invasion by down-regulating MMP2 and MMP9 [396].

Moreover, falcarindiol, an *Ostericum koreanum* Kitagawa-derived natural product, inhibits proliferation, migration, and invasion (Table 6). Falcarindiol also induces apoptosis and autophagy by inactivating PI3K/AKT/mTOR and activating ERK1/2 and p38 in oral cancer cells [402]. Artemisinin, a natural antimalarial product, suppresses uveal melanoma cell migration and invasion ability by inactivating PI3K/AKT/mTOR, which is reverted by AKT or mTOR activators such as Sc79 and MHY1485 [452]. Except for antiproliferation, melatonin induces anti-migration, anti-invasion, and apoptosis in gallbladder cancer cells by suppressing PI3K/AKT/mTOR, which is reverted by the antioxidant *N*-acetylcysteine [453]. This suggests that melatonin affects proliferation, apoptosis, and migration in a ROS-dependent manner. Additionally, fisetin blocks epidermal growth factor-induced migration of retinal pigment epithelial cells by inactivating AKT and down-regulating MMP-9 expression [439]. Myricetin, the grape-derived flavonoid, inhibits the angiogenesis of endothelial cells by inactivating AKT [383]. Crocin suppresses invasion of cervical cancer cells by activating AKT [400]. Crocetin, a saffron-derived carotenoid, enhances the angiogenesis of endothelial cells by activating AKT [434].

### 6.10. Natural Products Targeting Cell-Cycle Progression through AKT

Carvacrol, an aromatic plant-derived natural product, induces G1 arrest of breast cancer cells by suppressing CDK4, CDK6, pRB, and cyclin D1 expressions, accompanied by down-regulating PI3/AKT [438]. Carvacrol also blocks the cell-cycle progression of cervical cancer cells [454]. Glaucocalyxin A, a *Rabdosia japonica*-derived natural product, causes G2/M arrest to block the cell-cycle progression of melanoma cells [440]. Glycyrrhizin, a licorice roots-derived bioactive compound, initiates apoptosis and halts cell-cycle progression in cervical cancer cells [441]. Paclitaxel causes G2/M arrest of mammary tumor cells by down-regulating AKT [442]. Fisetin causes G1 arrest in prostate cancer cells, accompanied by inactivating AKT [436]. Proanthocyanidin induces G2/M arrest of pancreatic cancer cells by inhibiting AKT [389]. Safranal, a saffron-derived natural product, induces G2/M arrest of colon cancer cells by inactivating AKT [443].

## 7. Natural Products Regulate AKT Effectors-Modulated Cell Functions

Several natural products modulate several cell functions through AKT effector modulation (Section 7.1, Section 7.2, Section 7.3, Section 7.4, Section 7.5, Section 7.6, Section 7.7, Section 7.8, Section 7.9 and Section 7.10) (Table 7).

### 7.1. Natural Products Targeting Apoptosis through AKT effectors

Several studies investigated the connection between natural products and AKT effectors-triggered apoptosis (Table 7). For example, juglanthraquinone C, a *Juglans mandshurica*-derived compound, promotes ROS generation and triggers apoptosis of liver cancer cells by up-regulating FOXO signaling [455]. Dioscin, a natural steroidal saponin, triggers apoptosis of colon cancer cells by enhancing c-Myc ubiquitination [461].

A combined treatment of the mTORC1/2 inhibitor PP242 with curcumin promotes apoptosis of renal cancer cells [469]. Cucurbitacin B, a Thai herb *Trichosanthes cucumerina*-derived compound, triggers apoptosis by inhibiting MTORC1 expression in gastric cancer cells [468]. Neferine, a *Nelumbo nucifera*-derived compound, inhibits S6K1 and enhances apoptosis of neuroblastoma cells [474]. Emodin, a natural anthraquinone product, triggers apoptosis of liver cancer cells in SREBP1-dependent and -independent manners [477]. Luteolin, a natural flavonoid, blocks methylglyoxal-triggered apoptosis and increases cell proliferation of neuron cancer cells by inactivating mTOR/4EBP1 [481]. Moreover, salternamide A, a *Streptomyces*-derived natural product, inhibits hypoxia-promoted HIF1A overexpression and triggers apoptosis of colon cancer cells [484]. Grifolin triggers apoptosis of osteosarcoma cells by dephosphorylating AKT, FOXO, and GSK3 [488].

### 7.2. Natural Products Targeting Autophagy by AKT Effectors

The connection of natural products to AKT effector-triggered autophagy is widely investigated (Table 7). For example, the Brazil-wood-derived compound brazilin triggers FOXO3a-mediated autophagy and cell death by breaking calcium homeostasis [456]. 4-O-Methylascochlorin, a methylated derivative of ascochlorin, enhances autophagy of glioblastoma by inhibiting c-Myc [462]. Cucurbitacin B promotes autophagy of gastric cancer cells by down-regulating mTORC1 [468]. Additionally, neferine, a *Nelumbo nucifera*-derived S6K1 inhibitor, promotes autophagy of neuroblastoma cells [474]. Moreover, curcumin nicotinate inhibits SREBP1 expression of leukemia cells by recovering autophagy flux [478]. Parthenolide, a feverfew (*Tanacetum parthenium*)-derived plant compound, triggers autophagy by down-regulating 4EBP1 [482]. Moreover, geraniol, a monoterpene natural product, promotes the autophagy of CoCl2-treated lung cancer cells through the HIF1A/Beclin-1 pathway [485]. 11′-Deoxyverticillin A (C42) induces autophagy of colon cancer cells by up-regulating GSK3 expression [489].

### 7.3. Natural Products Targeting ER Stress through AKT Effectors

Except for FOXO, S6K1/2, and HIF, some AKT effectors such as c-Myc, mTORC1/2, SREBP1, and 4EBP1, were reported to regulate ER stress by natural product treatments (Table 7). For example, Z-ligustilide, a butanolide natural product, promotes c-Myc-mediated apoptosis of oral cancer cells by up-regulating ER stress signaling [463]. Tunicamycin, a natural antibiotic, enhances ER stress of prostate cancer cells by increasing mTORC1 expression [470]. Ginger extract exhibits mTOR-SREBP1-modulating function to regulate ER stress [479]. Moreover, α-solanine, a glycoalkaloid poison, induces autophagy and ER stress by down-regulating 4EBP1 [483]. Berberine, a *Coptis chinensis*-derived natural product, suppresses ER stress in amyloid precursor protein/PS1 mice by down-regulating GSK3β activity [490].

### 7.4. Natural Products Targeting Mitochondrial Morphogenesis through AKT Effectors

Except for GSK3, above AKT effectors were rarely investigated with respect to natural products that target mitochondrial fission/fusion. Resveratrol suppresses 1-methyl-4-phenylpyridinium-induced mitochondrial fission of nigral dopaminergic cells by up-regulating GSK3 expression [491].

### 7.5. Natural Products Targeting Ferroptosis through AKT Effectors

Some AKT effectors, such as FOXO, c-Myc, S6K1/2, SREBP1, and 4EBP1, were rarely investigated with respect to natural products that target ferroptosis. However, some AKT effectors, such as mTORC1/2 and HIF, were reported to regulate ferroptosis by natural product treatments (Table 7). For example, quercetin, a dietary flavonoid, triggers autophagy by down-regulating mTORC1 [471]. D-mannose inhibits HIF-2α to suppress chondrocyte ferroptosis [486]. Nobiletin, a citrus peel-derived flavonoid, causes ferroptosis of melanoma cells which is down-regulated by GSK3β knockdown [492].

### 7.6. Natural Products Targeting Necroptosis through AKT Effectors

AKT effectors provided by natural products that target necroptosis were rarely investigated.

### 7.7. Natural Products Targeting DNA Damage Response through AKT Effectors

Among these AKT effectors, only FOXO was reported to regulate DNA damage response by natural product treatments (Table 7). For example, purple corn extract reduces cigarette smoke-induced DNA damage to rodent blood cells by up-regulating FOX3a [457]. 6-Bromoindirubin-3′-oxime, a hemi-synthetic GSK3β inhibitor of indirubin derivative, inhibits DNA damage in fibroblasts [493].

### 7.8. Natural Products Targeting Senescence through AKT Effectors

Except for S6K1/2, SREBP1, and 4EBP1, some AKT effectors such as FOXO, c-Myc, mTORC1/2, and HIF were reported to regulate senescence by natural product treatments (Table 7). For example, resveratrol induces tumor suppressor DLC1-dependent senescence of breast cancer cells by down-regulating FOXO3a [458]. Oridonin triggers senescence of colon cancer cells by down-regulating c-Myc [464]. Baicalein and baicalin enhance the senescence of melanoma cells, suppressed by activating mTORC1-HIFα [472]. The GSK3β inhibitor of indirubin (6-bromoindirubin-3′-oxime) inhibits cellular senescence in fibroblasts [493].

### 7.9. Natural Products Targeting Migration through AKT Effectors

Except for mTORC1/2, SREBP1, and 4EBP1, some AKT effectors such as FOXO, c-Myc, S6K1/2, and HIF were reported to regulate migration by natural product treatments (Table 7). For example, sulforaphane, a sulfur-rich natural product, blocks angiogenesis by activating FOXO expression [459]. Ellagic acid suppresses acidity-promoted invasiveness of gastric cancer cells by down-regulating twist 1 and c-Myc expression [465]. 15(S)-hydroxyeicosatetraenoic acid enhances angiogenesis by activating S6K1 [475]. Furthermore, sulforaphane blocks the angiogenesis of colon cancer cells by down-regulating HIF1A and VEGF expression [487]. Nordentatin, an *Enkleia siamensis*-derived natural product, suppresses the migration of neuroblastoma cells by down-regulating GSK3 expression [494].

### 7.10. Natural Products Targeting Cell-Cycle Progression through AKT Effectors

Harmine hydrochloride, a *Peganum harmala*-derived alkaloid, caused G2/M arrest of breast cancer cells by down-regulating PI3K/AKT and up-regulating FOXO3a expression [460]. Berbamine, a Chinese medicinal herb-derived compound, induced G1 arrest of gastric cancer cells by down-regulating c-Myc expression [466]. Demethyleneberberine caused G1 arrest of lung cancer cells by down-regulating c-Myc/HIF1A [467]. 4-Hydroxyderricin, an *Angelica keiskei* Koidzumi-derived natural product, induced G1 or G2/M arrest for liver cancer cells (HepG2 and Huh7 cells) by down-regulating mTOR [473]. Bufadienolide, a traditional Chinese drug Chan’Su-derived cardiac glycoside, caused G1 arrest of breast and prostate cancer cells by inhibiting insulin-like growth factor-I (IGF1)-activated phosphorylation of mTOR, S6K1, and 4EBP1 [476]. Berberine caused G1 arrest of colon cancer cells by down-regulating SREBP1 expression [480]. Arctigenin induced G1 arrest of breast cancer cells by suppressing phosphorylation of GSK3 [495].

## 8. Conclusions

Oxidative stress and the AKT pathway exhibit versatile effects in regulating cell function for cancer cell development and treatment. However, current information generally focuses on some of them without a comprehensive integration, particularly for AKT effectors such as FOXO, c-Myc, mTORC1/2, S6K1/2, SREBP1, 4EBP1, HIF, and GSK3.

As mentioned above, the impacts of oxidative stress and the AKT pathway (AKT and its effector) are well integrated into several cell functions such as apoptosis, autophagy, ER stress, mitochondrial fission/fusion, ferroptosis, necroptosis, DNA damage response, senescence, migration and cell-cycle progression.

In addition to establishing the connection between oxidative stress, AKT pathway, and cell functions, their impacts on cancer treatment by natural products are summarized and evaluated. Mounting literature evidence shows that several anticancer natural products regulate oxidative stress and AKT pathway. Accordingly, natural products with modulating effects on oxidative stress and AKT pathway are expected to provide the potential for cancer cell function regulation and impacts on cancer therapy. However, the contribution of oxidative stress and AKT pathway are as yet rarely connected to cell functions in anticancer treatments using natural products. After a detailed literature search, some cell functions are attributed to the modulating effects of oxidative stress and the AKT pathway, although some are not reported.

It should be noted that many reports for oxidative stress and AKT pathway-associated cell functions cited in this review are derived from investigations of several cancer cell lines. Since the genetic mutations for these cancer cell lines differ, the association between oxidative stress, AKT pathway, and some cell functions may be restricted or become dominant only in some cancer cells or unique treatments. This warrants a careful investigation of these relationships if different cancer cells are concerned.

**Figure 2:**
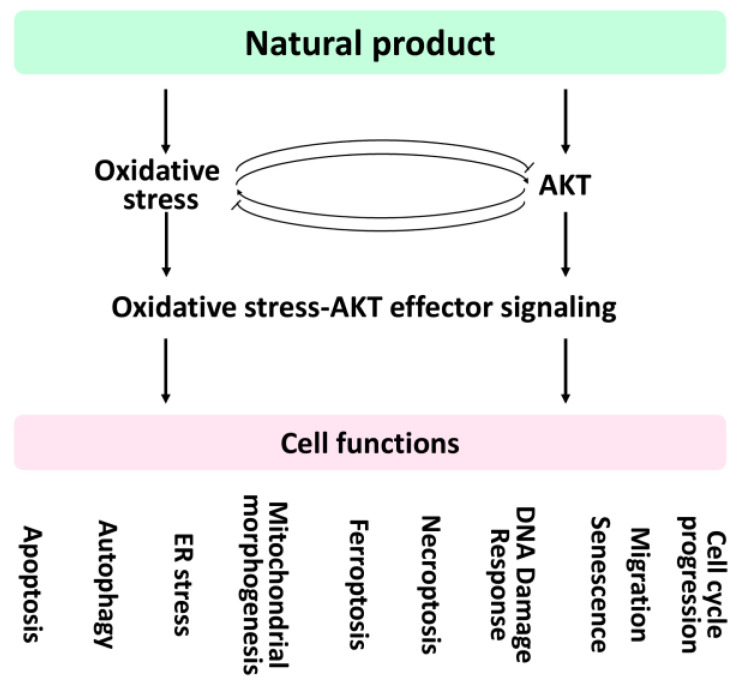
Hypothesis: Natural products modulating oxidative stress and the AKT pathway (AKT and AKT effectors) regulate several cell functions. The oxidative stress-AKT effector signaling is shown in Figure 1. Detailed future works are warranted to assess additional cell functions that are affected by natural products for anticancer treatment.

We hypothesize that the involvement of oxidative stress and AKT pathway in natural product performance also provides potential anticancer impacts through the modulation of several cell functions (Figure 2). This review sheds light on the impacts of oxidative stress and AKT pathway-regulated cell functions, providing a better understanding and future directions for curing cancer with natural products.

## Figures and Tables

**Table 1:** Oxidative stress regulates cell functions via several mediators.

	Cell Functions									
	Apoptosis	Autophagy	ER Stress	Mitochondrial Morphogenesis	Ferroptosis	Necroptosis	DNA Damage Response	Senescence	Migration	Cell-Cycle Progression
**Mediators**	PKC [33,34] SIRT1 [35] MAPK [36,37,38] AKT [39]	MAPK [40,41,42] AMPK [43] ULK1, mTORC1 [44,45]	PERK, IRE1α, ATF6 [46,47] MAPK [48,49]	DRP1 [50] FIS1 [51] MFN1 [52] MFN2 [53] MAPK [54]	GPX4 [55,56] ACSL4 [57,58] PTGS2 [59,60] CHAC1 [61,62] NRF2 [63] MAPK [64]	RIPK1, RIPK3, MLKL [65,66,67]	MAPK [68] OGG1 [69] APE1 [70]	SIRT1, SIRT3, SIRT6 [71] MAPK [72]	SIRT1, CDH1, CDH2, VIM [73] MAPK, PKCζ [74] AMPK [75]	AMPK [76,77] SIRT1 [78,79,80] MAPK [81,82]

**Table 2:** AKT regulates cell functions via several mediators.

	Cell Functions									
	Apoptosis	Autophagy	ER Stress	Mitochondrial Morphogenesis	Ferroptosis	Necroptosis	DNA Damage Response	Senescence	Migration	Cell-Cycle Progression
**Mediators**	PI3K, MAPK [133] mTOR [134,135]	PI3K, mTOR [136] ULK1 [137]	GRP78 [138] IRE1 [139] PERK [140] ATF6 [141] CHOP [142]	DRP1 [143] FIS1 [144] MFN1 [145] MFN2 [146]	GPX4 [147] ACSL4 [148] PTGS2 [149] CHAC1 [150]	RIPK1 [151] RIPK3 [152,153] MLKL [153]	DNA-PKcs [154] ATM [155,156] ATR [156] RAD51 [157] XPC [158]	SIRT1, SIRT3, SIRT6 [159] IL-1*α*, IL-1*β*, IL-6, IL-8 [160] p21 [161]	MMP2, MMP9 CDH1, CDH2 [162] VIM, SNAI1 [163]	AMPK [76,77] SIRT1 [78,79,80] MAPK [81,82]

**Table 3:** AMPK-SIRT1-MAPK network connects to AKT effectors.

	FOXO	c-Myc	mTORC1/2	S6K1/2	SREBP1	4EBP1	HIF	GSK3
**AMPK**	[212]	[213]	[214,215]	[216]	[217]	[216]	[218]	[13]
**SIRT1**	[219]	[220]	[221,222]	[223]	[224]	[221]	[225]	[226]
**MAPK**	[227,228]	[229]	[230,231]	[223]	[232]	[233]	[36]	[234]

**Table 4:** AKT effectors regulate cell functions.

	Cell functions									
AKT Effectors	Apoptosis	Autophagy	ER Stress	Mitochondrial Morphogenesis	Ferroptosis	Necroptosis	DNA Damage Response	Senescence	Migration	Cell-Cycle Progression
**FOXO**	[236,237]	[238,239,240]	[241,242]	[243]	[244]	[245]	[246,247]	[248,249]	[250,251]	[252]
**c-Myc**	[253]	[254]	[255,256]	[257]	[258,259]	[260]	[261,262]	[263]	[264]	[265]
**mTORC1/2**	[266,267,268]	[269,270,271]	[267]	[272]	[273,274]	[275]	[276,277]	[278,279]	[268,280]	[265]
**S6K1/2**	[281,282]	[282]	[283]	[272,284]	[285]	[187]	[286,287]	[288,289]	[290]	[291]
**SREBP1**	[292,293]	[293]	[294,295]	[296]	[273,285]	[297]	[298]	[299]	[300]	[301]
**4EBP1**	[267,302]	[303,304]	[267]	[272,305]	[306]	[307]	[308]	[309]	[310]	[311]
**HIF**	[312,313]	[314]	[315]	[316,317]	[318]	[319]	[320]	[321]	[314]	[322]
**GSK3**	[323,324]	[325]	[326]	[327]	[328]	[329]	[323,330]	[331]	[323]	[323]

X indicates the cell functions regulated by AKT effectors are not available according to Google Scholar and PubMed search (retrieval date: 16 June 2022).

**Table 5:** Oxidative Stress-modulating natural products that modulate cell functions.

	Cell Functions									
	Apoptosis	Autophagy	ER Stress	Mitochondrial Morphogenesis	Ferroptosis	Necroptosis	DNA Damage Response	Senescence	Migration	Cell-Cycle Progression
**Natural products**	Cryptocaryone [343] *Rubus fairholmianus*-derived compounds [30] Sanguinarine [344]	Isoaaptamine [345] Neferine [346] Piperlongumine [347]	Sarsasapogenin [348]	Icariin [349] T-2 toxin [350] Trehalose [351]	Apigenin [352] Tagitinin C [353]	Curcumol [354] Shikonin [355]	Cryptocaryone [343] Sinuleptolide [356]	Apigenin [357] Gingerenone A [358]	Salinomycin [359] Withaferin A [360]	Lycopene [361] Gracillin [362]

**Table 6:** AKT-modulated natural products that modulate cell functions.

	Cell Functions									
	Apoptosis	Autophagy	ER Stress	Mitochondrial Morphogenesis	Ferroptosis	Necroptosis	DNA Damage Response	Senescence	Migration	Cell-Cycle Progression
**Natural products**	Acetyl-lupeolic acid [365] Asperpyrone A [366] Bavachinin [367] Caffeic acid phenethyl ester [368] Crocin [369], CXC195 [370] 9-Demethylmucroniferanine A [371] Diallyl trisulfide [372] Emodin [373], Ferulin C [374] Fisetin [375], Fucoxanthin [376] Ginsenoside Rd [377] Ginsenoside Rh2 [378] Grincamycin B [379] 4β-Hydroxywithanolide E [380] Krukovine [381] Lupiwighteone [382] Myricetin [383] NAP [384] NVP-BEZ235 [385] Paeoniflorigenone [386] Piperine [387] Piperlongumine [388] Proanthocyanidin [389] Procyanidin B2 [390] Timosaponin TAIII [391] Trametenolic acid B [392] Troxerutin [393], Uvaol [394], YVPGP [395]	Alisol A [396] Allicin [397] Chaetocochin J [398] CLE-10 [399] Crocin [400] Echinatin [401] Falcarindiol [402] Fisetin [403] Geraniol [404] 6-Gingerol [405] Ilimaquinone [406] Neferine, Liensinine Isoliensinine [407] Paeoniflorigenone [386] Patulin [408] Procyanidin B2 [390] Spicatoside A [409] *Streptomyces* sp metabolite(s) [410] Tanshinone IIA [411]	Aromadendrin [412] Bleomycin [413] β-Carotene [414] Ginkgolide B [415] Methamphetamine [416] Procyanidin B2 [417]	Lupeol [418,419]	X	Paeoniflorigenone [386]	Cerberin [420] Chalcone [421] Cucurbitacin-A [422] Lanatoside C [423] Paclitaxel [424] Peruvoside [425] Romidepsin [426] Strophanthidin [427] TA25 [428]	Coroglaucigenin [429] Bleomycin [430] Doxycycline [431] Fisetin [432] Proanthocyanidins [433]	Alisol A [396] Crocetin [434] Fisetin [435,436] Myricetin [383] Platycodin D [437]	Carvacrol [438] Crocin [400] Fisetin [439] Glaucocalyxin A [440] Glycyrrhizin [441] Paclitaxel [442] Proanthocyanidin [389] Safranal [443]

X indicates those natural products regulated cell functions by AKT are not available according to Google Scholar and PubMed search (retrieval date: 16 June 2022).

**Table 7:** Natural products affecting cell functions through AKT effector modulation.

	Cell Functions									
AKT Effectors	Apoptosis	Autophagy	ER Stress	Mitochondrial Morphogenesis	Ferroptosis	Necroptosis	DNA Damage Response	Senescence	Migration	Cell-Cycle Progression
**FOXO**	Juglanthraquinone C [455]	Brazilin [456]	X	X	X	X	Purple corn extract [457]	Resveratrol [458]	Sulforaphane [459]	Harmine hydrochloride [460]
**c-Myc**	Dioscin [461]	4-O-Methylascochlorin [462]	Z-Ligustilide [463]	X	X	X	X	Oridonin [464]	Ellagic acid [465]	Berbamine [466], Demethyleneberberine [467]
**mTORC1/2**	Cucurbitacin B [468], PP242/Curcumin [469]	Cucurbitacin B [468]	Tunicamycin [470]	X	Quercetin [471]	X	X	Baicalein, baicalin [472]	X	4-Hydroxyderricin [473]
**S6K1/2**	Neferine [474]	Neferine [474]	X	X	X	X	X	X	15(S)-Hydroxyeicosatetraenoic acid [475]	Bufadienolide [476]
**SREBP1**	Emodin [477]	Curcumin nicotinate [478]	Ginger extract [479]	X	X	X	X	X	X	Berberine [480]
**4EBP1**	Luteolin [481]	Parthenolide [482]	α-Solanine [483]	X	X	X	X	X	X	Bufadienolide [476]
**HIF**	Salternamide A [484]	Geraniol [485]	X	X	D-mannose [486]	X	X	Baicalein, Baicalin [472]	Sulforaphane [487]	Demethyleneberberine [467]
**GSK3**	Grifolin [488]	11′-Deoxyverticillin A [489]	Berberine [490]	Resveratrol [491]	Nobiletin [492]	X	6-Bromoindirubin-3′-oxime [493]	6-Bromoindirubin-3′-oxime [493]	Nordentatin [494]	Arctigenin [495]

‘X’ indicates AKT effectors of natural product regulated cell functions that are not available according to a Google Scholar and PubMed search (retrieval date: 16 June 2022).

## Data Availability

Data are contained within the article.

## References

[1] Manning B.D., Toker A. (2017). AKT/PKB signaling: Navigating the network. Cell.

[2] Tang J.Y., Cheng Y.B., Chuang Y.T., Yang K.H., Chang F.R., Liu W., Chang H.W. (2022). Oxidative stress and AKT-associated angiogenesis in a zebrafish model and its potential application for withanolides. Cells.

[3] Carnero A. (2010). The PKB/AKT pathway in cancer. Curr. Pharm. Des..

[4] Osaki M., Oshimura M., Ito H. (2004). PI3K-Akt pathway: Its functions and alterations in human cancer. Apoptosis.

[5] Song M., Bode A.M., Dong Z., Lee M.H. (2019). AKT as a therapeutic target for cancer. Cancer Res..

[6] Yang W.L., Wu C.Y., Wu J., Lin H.K. (2010). Regulation of Akt signaling activation by ubiquitination. Cell Cycle.

[7] Sugiyama M.G., Fairn G.D., Antonescu C.N. (2019). Akt-ing up just about everywhere: Compartment-specific Akt activation and function in receptor tyrosine kinase signaling. Front. Cell Dev. Biol..

[8] Hoxhaj G., Manning B.D. (2020). The PI3K-AKT network at the interface of oncogenic signalling and cancer metabolism. Nat. Rev. Cancer.

[9] Bang O.S., Ha B.G., Park E.K., Kang S.S. (2000). Activation of Akt is induced by heat shock and involved in suppression of heat-shock-induced apoptosis of NIH3T3 cells. Biochem. Biophys. Res. Commun..

[10] Wan Y.S., Wang Z.Q., Shao Y., Voorhees J.J., Fisher G.J. (2001). Ultraviolet irradiation activates PI3-kinase/AKT survival pathway via EGF receptors in human skin in vivo. Int. J. Oncol..

[11] Stegeman H., Kaanders J.H., Wheeler D.L., van der Kogel A.J., Verheijen M.M., Waaijer S.J., Iida M., Grenman R., Span P.N., Bussink J. (2012). Activation of AKT by hypoxia: A potential target for hypoxic tumors of the head and neck. BMC Cancer.

[12] Shiau J.P., Chuang Y.T., Cheng Y.B., Tang J.Y., Hou M.F., Yen C.Y., Chang H.W. (2022). Impacts of oxidative stress and PI3K/AKT/mTOR on metabolism and the future direction of investigating fucoidan-modulated metabolism. Antioxidants.

[13] Suzuki T., Bridges D., Nakada D., Skiniotis G., Morrison S.J., Lin J.D., Saltiel A.R., Inoki K. (2013). Inhibition of AMPK catabolic action by GSK3. Mol. Cell.

[14] Betteridge D.J. (2000). What is oxidative stress?. Metabolism.

[15] Sies H., Fink G. (2019). Oxidative Stress. Stress: Physiology, Biochemistry, and Pathology.

[16] Azmanova M., Pitto-Barry A. (2022). Oxidative stress in cancer therapy: Friend or enemy?. Chembiochem.

[17] Karimian A., Mir S.M., Parsian H., Refieyan S., Mirza-Aghazadeh-Attari M., Yousefi B., Majidinia M. (2019). Crosstalk between Phosphoinositide 3-kinase/Akt signaling pathway with DNA damage response and oxidative stress in cancer. J. Cell Biochem..

[18] Koundouros N., Poulogiannis G. (2018). Phosphoinositide 3-Kinase/Akt signaling and redox metabolism in cancer. Front. Oncol..

[19] Okoh V.O., Felty Q., Parkash J., Poppiti R., Roy D. (2013). Reactive oxygen species via redox signaling to PI3K/AKT pathway contribute to the malignant growth of 4-hydroxy estradiol-transformed mammary epithelial cells. PLoS ONE.

[20] Rui W., Guan L., Zhang F., Zhang W., Ding W. (2016). PM2.5-induced oxidative stress increases adhesion molecules expression in human endothelial cells through the ERK/AKT/NF-kappaB-dependent pathway. J. Appl. Toxicol..

[21] Uranga R.M., Katz S., Salvador G.A. (2013). Enhanced phosphatidylinositol 3-kinase (PI3K)/Akt signaling has pleiotropic targets in hippocampal neurons exposed to iron-induced oxidative stress. J. Biol. Chem..

[22] Li D., Ni S., Miao K.S., Zhuang C. (2019). PI3K/Akt and caspase pathways mediate oxidative stress-induced chondrocyte apoptosis. Cell Stress Chaperones.

[23] Kim J.H., Choi T.G., Park S., Yun H.R., Nguyen N.N.Y., Jo Y.H., Jang M., Kim J., Kim J., Kang I. (2018). Mitochondrial ROS-derived PTEN oxidation activates PI3K pathway for mTOR-induced myogenic autophagy. Cell Death Differ..

[24] Hambright H.G., Meng P., Kumar A.P., Ghosh R. (2015). Inhibition of PI3K/AKT/mTOR axis disrupts oxidative stress-mediated survival of melanoma cells. Oncotarget.

[25] Wang F., Wang L., Qu C., Chen L., Geng Y., Cheng C., Yu S., Wang D., Yang L., Meng Z. (2021). Kaempferol induces ROS-dependent apoptosis in pancreatic cancer cells via TGM2-mediated Akt/mTOR signaling. BMC Cancer.

[26] Pang H., Wu T., Peng Z., Tan Q., Peng X., Zhan Z., Song L., Wei B. (2022). Baicalin induces apoptosis and autophagy in human osteosarcoma cells by increasing ROS to inhibit PI3K/Akt/mTOR, ERK1/2 and beta-catenin signaling pathways. J. Bone Oncol..

[27] Dolado I., Nebreda A.R. (2008). AKT and oxidative stress team up to kill cancer cells. Cancer Cell.

[28] Degtyarev M., De Maziere A., Orr C., Lin J., Lee B.B., Tien J.Y., Prior W.W., van Dijk S., Wu H., Gray D.C. (2008). Akt inhibition promotes autophagy and sensitizes PTEN-null tumors to lysosomotropic agents. J. Cell Biol..

[29] Kma L., Baruah T.J. (2022). The interplay of ROS and the PI3K/Akt pathway in autophagy regulation. Biotechnol. Appl. Biochem..

[30] George B.P., Abrahamse H. (2019). Increased oxidative stress induced by rubus bioactive compounds induce apoptotic cell death in human breast cancer cells. Oxidative Med. Cell. Longev..

[31] Tewari D., Patni P., Bishayee A., Sah A.N., Bishayee A. (2022). Natural products targeting the PI3K-Akt-mTOR signaling pathway in cancer: A novel therapeutic strategy. Semin Cancer Biol..

[32] Issinger O.G., Guerra B. (2021). Phytochemicals in cancer and their effect on the PI3K/AKT-mediated cellular signalling. Biomed. Pharmacother..

[33] Hu C.T., Wu J.R., Cheng C.C., Wang S., Wang H.T., Lee M.C., Wang L.J., Pan S.M., Chang T.Y., Wu W.S. (2011). Reactive oxygen species-mediated PKC and integrin signaling promotes tumor progression of human hepatoma HepG2. Clin. Exp. Metastasis.

[34] Inoguchi T., Sonta T., Tsubouchi H., Etoh T., Kakimoto M., Sonoda N., Sato N., Sekiguchi N., Kobayashi K., Sumimoto H. (2003). Protein kinase C-dependent increase in reactive oxygen species (ROS) production in vascular tissues of diabetes: Role of vascular NAD(P)H oxidase. J. Am. Soc. Nephrol..

[35] Salminen A., Kaarniranta K., Kauppinen A. (2013). Crosstalk between oxidative stress and sirt1: Impact on the aging process. Int. J. Mol. Sci..

[36] Sang N., Stiehl D.P., Bohensky J., Leshchinsky I., Srinivas V., Caro J. (2003). MAPK signaling up-regulates the activity of hypoxia-inducible factors by its effects on p300. J. Biol. Chem..

[37] Zhao Y., Luo P., Guo Q., Li S., Zhang L., Zhao M., Xu H., Yang Y., Poon W., Fei Z. (2012). Interactions between SIRT1 and MAPK/ERK regulate neuronal apoptosis induced by traumatic brain injury in vitro and in vivo. Exp. Neurol..

[38] Dong Y., Yin S., Song X., Huo Y., Fan L., Ye M., Hu H. (2016). Involvement of ROS-p38-H2AX axis in novel curcumin analogues-induced apoptosis in breast cancer cells. Mol. Carcinog..

[39] Rane M.J., Song Y., Jin S., Barati M.T., Wu R., Kausar H., Tan Y., Wang Y., Zhou G., Klein J.B. (2010). Interplay between Akt and p38 MAPK pathways in the regulation of renal tubular cell apoptosis associated with diabetic nephropathy. Am. J. Physiol. Renal. Physiol..

[40] Zhou Y.Y., Li Y., Jiang W.Q., Zhou L.F. (2015). MAPK/JNK signalling: A potential autophagy regulation pathway. Biosci. Rep..

[41] Sun Z.L., Dong J.L., Wu J. (2017). Juglanin induces apoptosis and autophagy in human breast cancer progression via ROS/JNK promotion. Biomed. Pharmacother..

[42] He Y., She H., Zhang T., Xu H., Cheng L., Yepes M., Zhao Y., Mao Z. (2018). p38 MAPK inhibits autophagy and promotes microglial inflammatory responses by phosphorylating ULK1. J. Cell. Biol..

[43] Yuan J., Dong X., Yap J., Hu J. (2020). The MAPK and AMPK signalings: Interplay and implication in targeted cancer therapy. J. Hematol. Oncol..

[44] Lee J.W., Park S., Takahashi Y., Wang H.G. (2010). The association of AMPK with ULK1 regulates autophagy. PLoS ONE.

[45] Holczer M., Hajdu B., Lorincz T., Szarka A., Banhegyi G., Kapuy O. (2020). Fine-tuning of AMPK-ULK1-mTORC1 regulatory triangle is crucial for autophagy oscillation. Sci. Rep..

[46] Ozcan L., Tabas I. (2012). Role of endoplasmic reticulum stress in metabolic disease and other disorders. Annu. Rev. Med..

[47] Farooqi A.A., Li K.T., Fayyaz S., Chang Y.T., Ismail M., Liaw C.C., Yuan S.S., Tang J.Y., Chang H.W. (2015). Anticancer drugs for the modulation of endoplasmic reticulum stress and oxidative stress. Tumour. Biol..

[48] Darling N.J., Cook S.J. (2014). The role of MAPK signalling pathways in the response to endoplasmic reticulum stress. Biochim. Biophys. Acta.

[49] Mishra R., Karande A.A. (2014). Endoplasmic reticulum stress-mediated activation of p38 MAPK, Caspase-2 and Caspase-8 leads to abrin-induced apoptosis. PLoS ONE.

[50] Cid-Castro C., Moran J. (2021). Differential ROS-mediated phosphorylation of Drp1 in mitochondrial fragmentation induced by distinct cell death conditions in cerebellar granule neurons. Oxidative Med. Cell. Longev..

[51] Qi X., Qvit N., Su Y.C., Mochly-Rosen D. (2013). A novel Drp1 inhibitor diminishes aberrant mitochondrial fission and neurotoxicity. J. Cell Sci..

[52] Yang X., Xue P., Chen H., Yuan M., Kang Y., Duscher D., Machens H.G., Chen Z. (2020). Denervation drives skeletal muscle atrophy and induces mitochondrial dysfunction, mitophagy and apoptosis via miR-142a-5p/MFN1 axis. Theranostics.

[53] Chakraborty P.K., Murphy B., Mustafi S.B., Dey A., Xiong X., Rao G., Naz S., Zhang M., Yang D., Dhanasekaran D.N. (2018). Cystathionine beta-synthase regulates mitochondrial morphogenesis in ovarian cancer. FASEB J..

[54] Ren L., Chen X., Chen X., Li J., Cheng B., Xia J. (2020). Mitochondrial dynamics: Fission and fusion in fate determination of mesenchymal stem cells. Front. Cell Dev. Biol..

[55] Sui X., Zhang R., Liu S., Duan T., Zhai L., Zhang M., Han X., Xiang Y., Huang X., Lin H. (2018). RSL3 drives ferroptosis through GPX4 inactivation and ROS production in colorectal cancer. Front. Pharmacol..

[56] Miao Y., Chen Y., Xue F., Liu K., Zhu B., Gao J., Yin J., Zhang C., Li G. (2022). Contribution of ferroptosis and GPX4’s dual functions to osteoarthritis progression. EBioMedicine.

[57] Chen J., Yang L., Geng L., He J., Chen L., Sun Q., Zhao J., Wang X. (2021). Inhibition of Acyl-CoA synthetase long-chain family member 4 facilitates neurological recovery after stroke by regulation ferroptosis. Front. Cell Neurosci..

[58] Qu X.F., Liang T.Y., Wu D.G., Lai N.S., Deng R.M., Ma C., Li X., Li H.Y., Liu Y.Z., Shen H.T. (2021). Acyl-CoA synthetase long chain family member 4 plays detrimental role in early brain injury after subarachnoid hemorrhage in rats by inducing ferroptosis. CNS Neurosci. Ther..

[59] Onodera Y., Teramura T., Takehara T., Shigi K., Fukuda K. (2015). Reactive oxygen species induce Cox-2 expression via TAK1 activation in synovial fibroblast cells. FEBS Open Bio..

[60] Dang S., Xu H., Xu C., Cai W., Li Q., Cheng Y., Jin M., Wang R.X., Peng Y., Zhang Y. (2014). Autophagy regulates the therapeutic potential of mesenchymal stem cells in experimental autoimmune encephalomyelitis. Autophagy.

[61] Crawford R.R., Prescott E.T., Sylvester C.F., Higdon A.N., Shan J., Kilberg M.S., Mungrue I.N. (2015). Human CHAC1 protein degrades glutathione, and mrna induction is regulated by the transcription factors ATF4 and ATF3 and a bipartite ATF/CRE regulatory element. J. Biol. Chem..

[62] Wang Z., Li M., Liu Y., Qiao Z., Bai T., Yang L., Liu B. (2021). Dihydroartemisinin triggers ferroptosis in primary liver cancer cells by promoting and unfolded protein response-induced upregulation of CHAC1 expression. Oncol. Rep..

[63] Dong H., Xia Y., Jin S., Xue C., Wang Y., Hu R., Jiang H. (2021). Nrf2 attenuates ferroptosis-mediated IIR-ALI by modulating TERT and SLC7A11. Cell Death Dis..

[64] Hattori K., Ishikawa H., Sakauchi C., Takayanagi S., Naguro I., Ichijo H. (2017). Cold stress-induced ferroptosis involves the ASK1-p38 pathway. EMBO Rep..

[65] Weinlich R., Oberst A., Beere H.M., Green D.R. (2017). Necroptosis in development, inflammation and disease. Nat. Rev. Mol. Cell Biol..

[66] Choi K., Kim J., Kim G.W., Choi C. (2009). Oxidative stress-induced necrotic cell death via mitochondira-dependent burst of reactive oxygen species. Curr. Neurovasc. Res..

[67] He L., He T., Farrar S., Ji L., Liu T., Ma X. (2017). Antioxidants maintain cellular redox homeostasis by elimination of reactive oxygen species. Cell Physiol. Biochem..

[68] Rezatabar S., Karimian A., Rameshknia V., Parsian H., Majidinia M., Kopi T.A., Bishayee A., Sadeghinia A., Yousefi M., Monirialamdari M. (2019). RAS/MAPK signaling functions in oxidative stress, DNA damage response and cancer progression. J. Cell Physiol..

[69] Bravard A., Vacher M., Gouget B., Coutant A., de Boisferon F.H., Marsin S., Chevillard S., Radicella J.P. (2006). Redox regulation of human OGG1 activity in response to cellular oxidative stress. Mol. Cell Biol..

[70] Luo M., He H., Kelley M.R., Georgiadis M.M. (2010). Redox regulation of DNA repair: Implications for human health and cancer therapeutic development. Antioxid Redox Signal..

[71] Kida Y., Goligorsky M.S. (2016). Sirtuins, cell senescence, and vascular aging. Can. J. Cardiol..

[72] Probin V., Wang Y., Zhou D. (2007). Busulfan-induced senescence is dependent on ROS production upstream of the MAPK pathway. Free Radic. Biol. Med..

[73] Sun T., Jiao L., Wang Y., Yu Y., Ming L. (2018). SIRT1 induces epithelial-mesenchymal transition by promoting autophagic degradation of E-cadherin in melanoma cells. Cell Death Dis..

[74] Huo Y.N., Chen W., Zheng X.X. (2015). ROS, MAPK/ERK and PKC play distinct roles in EGF-stimulated human corneal cell proliferation and migration. Cell Mol. Biol. (Noisy-le-grand).

[75] Heathcote H.R., Mancini S.J., Strembitska A., Jamal K., Reihill J.A., Palmer T.M., Gould G.W., Salt I.P. (2016). Protein kinase C phosphorylates AMP-activated protein kinase alpha1 Ser487. Biochem. J..

[76] Zhou X., Kuang Y., Liang S., Wang L. (2019). Metformin inhibits cell proliferation in SKM-1 cells via AMPK-mediated cell cycle arrest. J. Pharmacol. Sci..

[77] Liang J., Shao S.H., Xu Z.X., Hennessy B., Ding Z., Larrea M., Kondo S., Dumont D.J., Gutterman J.U., Walker C.L. (2007). The energy sensing LKB1-AMPK pathway regulates p27(kip1) phosphorylation mediating the decision to enter autophagy or apoptosis. Nat. Cell Biol..

[78] Zhang W., Feng Y., Guo Q., Guo W., Xu H., Li X., Yi F., Guan Y., Geng N., Wang P. (2020). SIRT1 modulates cell cycle progression by regulating CHK2 acetylation-phosphorylation. Cell Death Differ..

[79] He M., Yuan H., Tan B., Bai R., Kim H.S., Bae S., Che L., Kim J.S., Gao S.J. (2016). SIRT1-mediated downregulation of p27Kip1 is essential for overcoming contact inhibition of Kaposi’s sarcoma-associated herpesvirus transformed cells. Oncotarget.

[80] Hu Q., Wang G., Peng J., Qian G., Jiang W., Xie C., Xiao Y., Wang X. (2017). Knockdown of SIRT1 suppresses bladder cancer cell proliferation and migration and induces cell cycle arrest and antioxidant response through FOXO3a-mediated pathways. Biomed. Res. Int..

[81] Pumiglia K.M., Decker S.J. (1997). Cell cycle arrest mediated by the MEK/mitogen-activated protein kinase pathway. Proc. Natl. Acad. Sci. USA.

[82] Durandau E., Pelet S. (2021). Cross-regulation between CDK and MAPK control cellular fate. Quant. Biol..

[83] Ding H., Han C., Guo D., Chin Y.W., Ding Y., Kinghorn A.D., D’Ambrosio S.M. (2009). Selective induction of apoptosis of human oral cancer cell lines by avocado extracts via a ROS-mediated mechanism. Nutr. Cancer.

[84] Jeong J.C., Jang S.W., Kim T.H., Kwon C.H., Kim Y.K. (2010). Mulberry fruit (*Moris fructus*) extracts induce human glioma cell death in vitro through ROS-dependent mitochondrial pathway and inhibits glioma tumor growth in vivo. Nutr. Cancer.

[85] Widodo N., Priyandoko D., Shah N., Wadhwa R., Kaul S.C. (2010). Selective killing of cancer cells by Ashwagandha leaf extract and its component Withanone involves ROS signaling. PLoS ONE.

[86] Lee J.C., Hou M.F., Huang H.W., Chang F.R., Yeh C.C., Tang J.Y., Chang H.W. (2013). Marine algal natural products with anti-oxidative, anti-inflammatory, and anti-cancer properties. Cancer Cell Int..

[87] Samhan-Arias A.K., Martin-Romero F.J., Gutierrez-Merino C. (2004). Kaempferol blocks oxidative stress in cerebellar granule cells and reveals a key role for reactive oxygen species production at the plasma membrane in the commitment to apoptosis. Free Radic. Biol. Med..

[88] Oh S.H., Lim S.C. (2006). A rapid and transient ROS generation by cadmium triggers apoptosis via caspase-dependent pathway in HepG2 cells and this is inhibited through *N*-acetylcysteine-mediated catalase upregulation. Toxicol. Appl. Pharmacol..

[89] Li J.J., Tang Q., Li Y., Hu B.R., Ming Z.Y., Fu Q., Qian J.Q., Xiang J.Z. (2006). Role of oxidative stress in the apoptosis of hepatocellular carcinoma induced by combination of arsenic trioxide and ascorbic acid. Acta Pharmacol. Sin..

[90] Ehlers R.A., Hernandez A., Bloemendal L.S., Ethridge R.T., Farrow B., Evers B.M. (1999). Mitochondrial DNA damage and altered membrane potential (delta psi) in pancreatic acinar cells induced by reactive oxygen species. Surgery.

[91] Jeong C.H., Joo S.H. (2016). Downregulation of reactive oxygen species in apoptosis. J. Cancer Prev..

[92] Thompson J.W., Dave K.R., Saul I., Narayanan S.V., Perez-Pinzon M.A. (2013). Epsilon PKC increases brain mitochondrial SIRT1 protein levels via heat shock protein 90 following ischemic preconditioning in rats. PLoS ONE.

[93] Cho S.Y., Klemke R.L. (2000). Extracellular-regulated kinase activation and CAS/Crk coupling regulate cell migration and suppress apoptosis during invasion of the extracellular matrix. J. Cell Biol..

[94] Mizushima N., Klionsky D.J. (2007). Protein turnover via autophagy: Implications for metabolism. Annu. Rev. Nutr..

[95] Gibson S.B. (2013). Investigating the role of reactive oxygen species in regulating autophagy. Methods Enzymol..

[96] Forman H.J., Zhang H. (2021). Targeting oxidative stress in disease: Promise and limitations of antioxidant therapy. Nat. Rev. Drug Discov..

[97] Poillet-Perez L., Despouy G., Delage-Mourroux R., Boyer-Guittaut M. (2015). Interplay between ROS and autophagy in cancer cells, from tumor initiation to cancer therapy. Redox Biol..

[98] Son Y., Cheong Y.K., Kim N.H., Chung H.T., Kang D.G., Pae H.O. (2011). Mitogen-activated protein kinases and reactive oxygen species: How can ROS activate MAPK pathways?. J. Signal. Transduct..

[99] Hetz C., Papa F.R. (2018). The unfolded protein response and cell fate control. Mol. Cell.

[100] Fernandez A., Ordonez R., Reiter R.J., Gonzalez-Gallego J., Mauriz J.L. (2015). Melatonin and endoplasmic reticulum stress: Relation to autophagy and apoptosis. J. Pineal Res..

[101] Zhang X., Chen M., Zou P., Kanchana K., Weng Q., Chen W., Zhong P., Ji J., Zhou H., He L. (2015). Curcumin analog WZ35 induced cell death via ROS-dependent ER stress and G2/M cell cycle arrest in human prostate cancer cells. BMC Cancer.

[102] Chen Y., Zhang H., Zhou H.J., Ji W., Min W. (2016). Mitochondrial redox signaling and tumor progression. Cancers.

[103] Sabouny R., Shutt T.E. (2020). Reciprocal regulation of mitochondrial fission and fusion. Trends Biochem. Sci..

[104] Lee W.C., Chiu C.H., Chen J.B., Chen C.H., Chang H.W. (2016). Mitochondrial fission increases apoptosis and decreases autophagy in renal proximal tubular epithelial cells treated with high glucose. DNA Cell Biol..

[105] Hoppins S., Nunnari J. (2009). The molecular mechanism of mitochondrial fusion. Biochim. Biophys. Acta.

[106] Lackner L.L., Nunnari J.M. (2009). The molecular mechanism and cellular functions of mitochondrial division. Biochim. Biophys. Acta.

[107] Otera H., Wang C., Cleland M.M., Setoguchi K., Yokota S., Youle R.J., Mihara K. (2010). Mff is an essential factor for mitochondrial recruitment of Drp1 during mitochondrial fission in mammalian cells. J. Cell Biol..

[108] Loson O.C., Song Z., Chen H., Chan D.C. (2013). Fis1, Mff, MiD49, and MiD51 mediate Drp1 recruitment in mitochondrial fission. Mol. Biol. Cell.

[109] Kim I., Rodriguez-Enriquez S., Lemasters J.J. (2007). Selective degradation of mitochondria by mitophagy. Arch Biochem. Biophys..

[110] Tur J., Pereira-Lopes S., Vico T., Marin E.A., Munoz J.P., Hernandez-Alvarez M., Cardona P.J., Zorzano A., Lloberas J., Celada A. (2020). Mitofusin 2 in macrophages links mitochondrial ROS production, cytokine release, phagocytosis, autophagy, and bactericidal activity. Cell Rep..

[111] Youle R.J., Karbowski M. (2005). Mitochondrial fission in apoptosis. Nat. Rev. Mol. Cell Biol..

[112] Zhu J., Xiong Y., Zhang Y., Wen J., Cai N., Cheng K., Liang H., Zhang W. (2020). The molecular mechanisms of regulating oxidative stress-induced ferroptosis and therapeutic strategy in tumors. Oxidative Med. Cell. Longev..

[113] Zheng D.W., Lei Q., Zhu J.Y., Fan J.X., Li C.X., Li C., Xu Z., Cheng S.X., Zhang X.Z. (2017). Switching apoptosis to ferroptosis: Metal-organic network for high-efficiency anticancer therapy. Nano Lett..

[114] Su Y., Zhao B., Zhou L., Zhang Z., Shen Y., Lv H., AlQudsy L.H.H., Shang P. (2020). Ferroptosis, a novel pharmacological mechanism of anti-cancer drugs. Cancer Lett..

[115] Chen X., Comish P.B., Tang D., Kang R. (2021). Characteristics and biomarkers of ferroptosis. Front. Cell Dev. Biol..

[116] Lee G.H., Lee W.J., Hur J., Kim E., Lee H.G., Seo H.G. (2020). Ginsenoside Re mitigates 6-hydroxydopamine-induced oxidative stress through upregulation of GPX4. Molecules.

[117] Zhuang Y., Wang C., Wu C., Ding D., Zhao F., Hu C., Gong W., Ding G., Zhang Y., Chen L. (2018). Mitochondrial oxidative stress activates COX-2/mPGES-1/PGE2 cascade induced by albumin in renal proximal tubular cells. Oncotarget.

[118] Xie Y., Hou W., Song X., Yu Y., Huang J., Sun X., Kang R., Tang D. (2016). Ferroptosis: Process and function. Cell Death Differ..

[119] Antonuccio P., Minutoli L., Romeo C., Nicotina P.A., Bitto A., Arena S., Altavilla D., Zuccarello B., Polito F., Squadrito F. (2006). Lipid peroxidation activates mitogen-activated protein kinases in testicular ischemia-reperfusion injury. J. Urol..

[120] Troyano A., Fernandez C., Sancho P., de Blas E., Aller P. (2001). Effect of glutathione depletion on antitumor drug toxicity (apoptosis and necrosis) in U-937 human promonocytic cells. The role of intracellular oxidation. J. Biol. Chem..

[121] Mohanty S., Yadav P., Lakshminarayanan H., Sharma P., Vivekanandhan A., Karunagaran D. (2022). RETRA induces necroptosis in cervical cancer cells through RIPK1, RIPK3, MLKL and increased ROS production. Eur. J. Pharmacol..

[122] Li L., Tan H., Zou Z., Gong J., Zhou J., Peng N., Su L., Maegele M., Cai D., Gu Z. (2020). Preventing necroptosis by scavenging ROS production alleviates heat stress-induced intestinal injury. Int. J. Hyperth..

[123] Qin S., Yang C., Huang W., Du S., Mai H., Xiao J., Lu T. (2018). Sulforaphane attenuates microglia-mediated neuronal necroptosis through down-regulation of MAPK/NF-kappaB signaling pathways in LPS-activated BV-2 microglia. Pharmacol. Res..

[124] Xie X., Zhao Y., Ma C.Y., Xu X.M., Zhang Y.Q., Wang C.G., Jin J., Shen X., Gao J.L., Li N. (2015). Dimethyl fumarate induces necroptosis in colon cancer cells through GSH depletion/ROS increase/MAPKs activation pathway. Br. J. Pharmacol..

[125] Kello M., Takac P., Kubatka P., Kuruc T., Petrova K., Mojzis J. (2020). Oxidative stress-induced DNA damage and apoptosis in clove buds-treated MCF-7 cells. Biomolecules.

[126] Gonzalez-Quiroz M., Blondel A., Sagredo A., Hetz C., Chevet E., Pedeux R. (2020). When endoplasmic reticulum proteostasis meets the DNA damage response. Trends Cell Biol..

[127] Srinivas U.S., Tan B.W.Q., Vellayappan B.A., Jeyasekharan A.D. (2019). ROS and the DNA damage response in cancer. Redox Biol..

[128] Tsai M.S., Weng S.H., Chen H.J., Chiu Y.F., Huang Y.C., Tseng S.C., Kuo Y.H., Lin Y.W. (2012). Inhibition of p38 MAPK-dependent excision repair cross-complementing 1 expression decreases the DNA repair capacity to sensitize lung cancer cells to etoposide. Mol. Cancer Ther..

[129] Brandl A., Hartmann A., Bechmann V., Graf B., Nerlich M., Angele P. (2011). Oxidative stress induces senescence in chondrocytes. J. Orthop. Res..

[130] Brandl A., Meyer M., Bechmann V., Nerlich M., Angele P. (2011). Oxidative stress induces senescence in human mesenchymal stem cells. Exp. Cell Res..

[131] Ota H., Tokunaga E., Chang K., Hikasa M., Iijima K., Eto M., Kozaki K., Akishita M., Ouchi Y., Kaneki M. (2006). Sirt1 inhibitor, Sirtinol, induces senescence-like growth arrest with attenuated Ras-MAPK signaling in human cancer cells. Oncogene.

[132] Ruderman N.B., Xu X.J., Nelson L., Cacicedo J.M., Saha A.K., Lan F., Ido Y. (2010). AMPK and SIRT1: A long-standing partnership?. Am. J. Physiol. Endocrinol. Metab..

[133] Fan Y.H., Ding H.W., Kim D., Liu J.Y., Hong J.Y., Xu Y.N., Wang D., Yang X.S., Lee S.K. (2020). The PI3Kalpha inhibitor DFX24 suppresses tumor growth and metastasis in non-small cell lung cancer via ERK inhibition and EPHB6 reactivation. Pharmacol. Res..

[134] Hahn-Windgassen A., Nogueira V., Chen C.C., Skeen J.E., Sonenberg N., Hay N. (2005). Akt activates the mammalian target of rapamycin by regulating cellular ATP level and AMPK activity. J. Biol. Chem..

[135] Castedo M., Ferri K.F., Kroemer G. (2002). Mammalian target of rapamycin (mTOR): Pro- and anti-apoptotic. Cell Death Differ..

[136] Xu Z., Han X., Ou D., Liu T., Li Z., Jiang G., Liu J., Zhang J. (2020). Targeting PI3K/AKT/mTOR-mediated autophagy for tumor therapy. Appl. Microbiol. Biotechnol..

[137] Rajak S., Iannucci L.F., Zhou J., Anjum B., George N., Singh B.K., Ghosh S., Yen P.M., Sinha R.A. (2020). Loss of ULK1 attenuates cholesterogenic gene expression in mammalian hepatic cells. Front. Cell Dev. Biol..

[138] Casas C. (2017). GRP78 at the centre of the stage in cancer and neuroprotection. Front. Neurosci..

[139] Han C.Y., Lim S.W., Koo J.H., Kim W., Kim S.G. (2016). PHLDA3 overexpression in hepatocytes by endoplasmic reticulum stress via IRE1-Xbp1s pathway expedites liver injury. Gut.

[140] Bobrovnikova-Marjon E., Pytel D., Riese M.J., Vaites L.P., Singh N., Koretzky G.A., Witze E.S., Diehl J.A. (2012). PERK utilizes intrinsic lipid kinase activity to generate phosphatidic acid, mediate Akt activation, and promote adipocyte differentiation. Mol. Cell Biol..

[141] Stengel S.T., Fazio A., Lipinski S., Jahn M.T., Aden K., Ito G., Wottawa F., Kuiper J.W.P., Coleman O.I., Tran F. (2020). Activating transcription factor 6 mediates inflammatory signals in intestinal epithelial cells upon endoplasmic reticulum stress. Gastroenterology.

[142] Hyoda K., Hosoi T., Horie N., Okuma Y., Ozawa K., Nomura Y. (2006). PI3K-Akt inactivation induced CHOP expression in endoplasmic reticulum-stressed cells. Biochem. Biophys. Res. Commun..

[143] Kim D.I., Lee K.H., Gabr A.A., Choi G.E., Kim J.S., Ko S.H., Han H.J. (2016). Aβ-Induced Drp1 phosphorylation through Akt activation promotes excessive mitochondrial fission leading to neuronal apoptosis. Biochim. Biophys. Acta (BBA)-Mol. Cell Res..

[144] Shi J., Yu J., Zhang Y., Li Z., Gong L., Dong S., Mu R. (2018). Phosphatidylinositol 3-kinase-mediated HO-1/CO represses Fis1 levels and alleviates lipopolysaccharide-induced oxidative injury in alveolar macrophages. Exp. Ther. Med..

[145] Ong S.B., Hall A.R., Dongworth R.K., Kalkhoran S., Pyakurel A., Scorrano L., Hausenloy D.J. (2015). Akt protects the heart against ischaemia-reperfusion injury by modulating mitochondrial morphology. Thromb. Haemost..

[146] Yi S., Cui C., Huang X., Yin X., Li Y., Wen J., Luan Q. (2020). MFN2 silencing promotes neural differentiation of embryonic stem cells via the Akt signaling pathway. J. Cell Physiol..

[147] Lee S.-H. (2008). Insulin-induced GPX4 expression in breast cancer cells. J. Soonchunhyang Med. Sci..

[148] Liu L., Li Y., Cao D., Qiu S., Li Y., Jiang C., Bian R., Yang Y., Li L., Li X. (2021). SIRT3 inhibits gallbladder cancer by induction of AKT-dependent ferroptosis and blockade of epithelial-mesenchymal transition. Cancer Lett..

[149] Zhang Z. (2021). MiR-124-3p suppresses prostatic carcinoma by targeting PTGS2 through the AKT/NF-κB pathway. Mol. Biotechnol..

[150] Liu Y., Li M., Shi D., Zhu Y. (2019). Higher expression of cation transport regulator-like protein 1 (CHAC1) predicts of poor outcomes in uveal melanoma (UM) patients. Int. Ophthalmol..

[151] Huang H., Chen T., Zhou Y., Geng L., Shen T., Zhou L., Zheng S. (2018). RIPK1 inhibition enhances pirarubicin cytotoxic efficacy through AKT-P21-dependent pathway in hepatocellular carcinoma. Int. J. Med. Sci..

[152] Imamura M., Moon J.S., Chung K.P., Nakahira K., Muthukumar T., Shingarev R., Ryter S.W., Choi A.M., Choi M.E. (2018). RIPK3 promotes kidney fibrosis via AKT-dependent ATP citrate lyase. JCI Insight.

[153] Huang D., Chen P., Huang G., Sun H., Luo X., He C., Chen F., Wang Y., Zeng C., Su L. (2022). Salt-inducible kinases inhibitor HG-9-91-01 targets RIPK3 kinase activity to alleviate necroptosis-mediated inflammatory injury. Cell Death Dis..

[154] Glorieux M., Dok R., Nuyts S. (2020). The influence of PI3K inhibition on the radiotherapy response of head and neck cancer cells. Sci. Rep..

[155] Toulany M., Rodemann H.P. (2013). Potential of Akt mediated DNA repair in radioresistance of solid tumors overexpressing erbB-PI3K-Akt pathway. Transl. Cancer Res..

[156] Xie Y., Liu C., Zhang Y., Li A., Sun C., Li R., Xing Y., Shi M., Wang Q. (2021). PKI-587 enhances radiosensitization of hepatocellular carcinoma by inhibiting the PI3K/AKT/mTOR pathways and DNA damage repair. PLoS ONE.

[157] Boichuk S., Bikinieva F., Nurgatina I., Dunaev P., Valeeva E., Aukhadieva A., Sabirov A., Galembikova A. (2020). Inhibition of AKT-signaling sensitizes soft tissue sarcomas (STS) and gastrointestinal stromal tumors (GIST) to doxorubicin via targeting of homology-mediated DNA repair. Int. J. Mol. Sci..

[158] Teng X., Fan X.F., Li Q., Liu S., Wu D.Y., Wang S.Y., Shi Y., Dong M. (2019). XPC inhibition rescues cisplatin resistance via the Akt/mTOR signaling pathway in A549/DDP lung adenocarcinoma cells. Oncol. Rep..

[159] Pillai V.B., Sundaresan N.R., Gupta M.P. (2014). Regulation of Akt signaling by sirtuins: Its implication in cardiac hypertrophy and aging. Circ. Res..

[160] Astle M.V., Hannan K.M., Ng P.Y., Lee R.S., George A.J., Hsu A.K., Haupt Y., Hannan R.D., Pearson R.B. (2012). AKT induces senescence in human cells via mTORC1 and p53 in the absence of DNA damage: Implications for targeting mTOR during malignancy. Oncogene.

[161] Miyauchi H., Minamino T., Tateno K., Kunieda T., Toko H., Komuro I. (2004). Akt negatively regulates the *in vitro* lifespan of human endothelial cells via a p53/p21-dependent pathway. EMBO J..

[162] Tian K., Du G., Wang X., Wu X., Li L., Liu W., Wu R. (2022). MMP-9 secreted by M2-type macrophages promotes Wilms’ tumour metastasis through the PI3K/AKT pathway. Mol. Biol. Rep..

[163] Dong J., Zhai B., Sun W., Hu F., Cheng H., Xu J. (2017). Activation of phosphatidylinositol 3-kinase/AKT/snail signaling pathway contributes to epithelial-mesenchymal transition-induced multi-drug resistance to sorafenib in hepatocellular carcinoma cells. PLoS ONE.

[164] Lee E.R., Kim J.Y., Kang Y.J., Ahn J.Y., Kim J.H., Kim B.W., Choi H.Y., Jeong M.Y., Cho S.G. (2006). Interplay between PI3K/Akt and MAPK signaling pathways in DNA-damaging drug-induced apoptosis. Biochim. Biophys. Acta.

[165] Braicu C., Buse M., Busuioc C., Drula R., Gulei D., Raduly L., Rusu A., Irimie A., Atanasov A.G., Slaby O. (2019). A comprehensive review on MAPK: A promising therapeutic target in cancer. Cancers.

[166] Aksamitiene E., Kiyatkin A., Kholodenko B.N. (2012). Cross-talk between mitogenic Ras/MAPK and survival PI3K/Akt pathways: A fine balance. Biochem. Soc. Trans..

[167] Menges C.W., McCance D.J. (2008). Constitutive activation of the Raf-MAPK pathway causes negative feedback inhibition of Ras-PI3K-AKT and cellular arrest through the EphA2 receptor. Oncogene.

[168] Turke A.B., Song Y., Costa C., Cook R., Arteaga C.L., Asara J.M., Engelman J.A. (2012). MEK inhibition leads to PI3K/AKT activation by relieving a negative feedback on ERBB receptors. Cancer Res..

[169] Auciello F.R., Ross F.A., Ikematsu N., Hardie D.G. (2014). Oxidative stress activates AMPK in cultured cells primarily by increasing cellular AMP and/or ADP. FEBS Lett..

[170] Zhao Y., Hu X., Liu Y., Dong S., Wen Z., He W., Zhang S., Huang Q., Shi M. (2017). ROS signaling under metabolic stress: Cross-talk between AMPK and AKT pathway. Mol. Cancer.

[171] Zhao E., Hou J., Ke X., Abbas M.N., Kausar S., Zhang L., Cui H. (2019). The roles of sirtuin family proteins in cancer progression. Cancers.

[172] Wu S.Y., Pan S.L., Xiao Z.Y., Hsu J.L., Chen M.C., Lee K.H., Teng C.M. (2014). NPRL-Z-1, as a new topoisomerase II poison, induces cell apoptosis and ROS generation in human renal carcinoma cells. PLoS ONE.

[173] Ni H., Guo M., Zhang X., Jiang L., Tan S., Yuan J., Cui H., Min Y., Zhang J., Schlisio S. (2021). VEGFR2 inhibition hampers breast cancer cell proliferation via enhanced mitochondrial biogenesis. Cancer Biol. Med..

[174] Hao X., Deng J., Zhang H., Liang Z., Lei F., Wang Y., Yang X., Wang Z. (2021). Design, synthesis and bioactivity evaluation of novel N-phenyl-substituted evodiamine derivatives as potent anti-tumor agents. Bioorg. Med. Chem..

[175] Wang J., Liang D., Zhang X.P., He C.F., Cao L., Zhang S.Q., Xiao X., Li S.J., Cao Y.X. (2020). Novel PI3K/Akt/mTOR signaling inhibitor, W922, prevents colorectal cancer growth via the regulation of autophagy. Int. J. Oncol..

[176] Wang X.Y., Zhang X.H., Peng L., Liu Z., Yang Y.X., He Z.X., Dang H.W., Zhou S.F. (2017). Bardoxolone methyl (CDDO-Me or RTA402) induces cell cycle arrest, apoptosis and autophagy via PI3K/Akt/mTOR and p38 MAPK/Erk1/2 signaling pathways in K562 cells. Am. J. Transl. Res..

[177] Sun X., Wang T., Huang B., Ruan G., Xu A. (2020). RIPK1 regulates the survival of human melanocytes upon endoplasmic reticulum stress. Exp. Ther. Med..

[178] Sanchez-Alvarez M., Del Pozo M.A., Bakal C. (2017). AKT-mTOR signaling modulates the dynamics of IRE1 RNAse activity by regulating ER-mitochondria contacts. Sci. Rep..

[179] Yamazaki H., Hiramatsu N., Hayakawa K., Tagawa Y., Okamura M., Ogata R., Huang T., Nakajima S., Yao J., Paton A.W. (2009). Activation of the Akt-NF-kappaB pathway by subtilase cytotoxin through the ATF6 branch of the unfolded protein response. J. Immunol..

[180] Ansari S.S., Sharma A.K., Soni H., Ali D.M., Tews B., Konig R., Eibl H., Berger M.R. (2018). Induction of ER and mitochondrial stress by the alkylphosphocholine erufosine in oral squamous cell carcinoma cells. Cell Death Dis..

[181] Wang Y., Zhao H., Shao Y., Liu J., Li J., Luo L., Xing M. (2018). Copper or/and arsenic induces autophagy by oxidative stress-related PI3K/AKT/mTOR pathways and cascaded mitochondrial fission in chicken skeletal muscle. J. Inorg. Biochem..

[182] Humphries B.A., Cutter A.C., Buschhaus J.M., Chen Y.C., Qyli T., Palagama D.S.W., Eckley S., Robison T.H., Bevoor A., Chiang B. (2020). Enhanced mitochondrial fission suppresses signaling and metastasis in triple-negative breast cancer. Breast Cancer Res..

[183] Xie X., Shu R., Yu C., Fu Z., Li Z. (2022). Mammalian AKT, the Emerging roles on mitochondrial function in diseases. Aging Dis..

[184] Li G., Yang J., Zhao G., Shen Z., Yang K., Tian L., Zhou Q., Chen Y., Huang Y. (2021). Dysregulation of ferroptosis may involve in the development of non-small-cell lung cancer in Xuanwei area. J. Cell Mol. Med..

[185] Zhang L., Wang H., Ding K., Xu J. (2015). FTY720 induces autophagy-related apoptosis and necroptosis in human glioblastoma cells. Toxicol. Lett..

[186] Li H., Hu J., Wu S., Wang L., Cao X., Zhang X., Dai B., Cao M., Shao R., Zhang R. (2016). Auranofin-mediated inhibition of PI3K/AKT/mTOR axis and anticancer activity in non-small cell lung cancer cells. Oncotarget.

[187] McNamara C.R., Ahuja R., Osafo-Addo A.D., Barrows D., Kettenbach A., Skidan I., Teng X., Cuny G.D., Gerber S., Degterev A. (2013). Akt Regulates TNFalpha synthesis downstream of RIP1 kinase activation during necroptosis. PLoS ONE.

[188] Zhao M., Lu L., Lei S., Chai H., Wu S., Tang X., Bao Q., Chen L., Wu W., Liu X. (2016). Inhibition of receptor interacting protein kinases attenuates cardiomyocyte hypertrophy induced by palmitic acid. Oxidative Med. Cell. Longev..

[189] Chen Z., Chen C., Zhou T., Duan C., Wang Q., Zhou X., Zhang X., Wu F., Hua Y., Lin F. (2020). A high-throughput drug combination screen identifies an anti-glioma synergism between TH588 and PI3K inhibitors. Cancer Cell Int..

[190] Davis W.J., Lehmann P.Z., Li W. (2015). Nuclear PI3K signaling in cell growth and tumorigenesis. Front. Cell Dev. Biol..

[191] Xu N., Lao Y., Zhang Y., Gillespie D.A. (2012). Akt: A double-edged sword in cell proliferation and genome stability. J. Oncol..

[192] Huang T.T., Lampert E.J., Coots C., Lee J.M. (2020). Targeting the PI3K pathway and DNA damage response as a therapeutic strategy in ovarian cancer. Cancer Treat. Rev..

[193] Bent E.H., Gilbert L.A., Hemann M.T. (2016). A senescence secretory switch mediated by PI3K/AKT/mTOR activation controls chemoprotective endothelial secretory responses. Genes Dev..

[194] Haines C.N., Klingensmith H.D., Komara M., Burd C.J. (2020). GREB1 regulates PI3K/Akt signaling to control hormone-sensitive breast cancer proliferation. Carcinogenesis.

[195] Kim Y.Y., Jee H.J., Um J.H., Kim Y.M., Bae S.S., Yun J. (2017). Cooperation between p21 and Akt is required for p53-dependent cellular senescence. Aging Cell.

[196] Kumari R., Jat P. (2021). Mechanisms of cellular senescence: Cell cycle arrest and senescence associated secretory phenotype. Front. Cell Dev. Biol..

[197] Matic I., Revandkar A., Chen J., Bisio A., Dall’Acqua S., Cocetta V., Brun P., Mancino G., Milanese M., Mattei M. (2016). Identification of *Salvia haenkei* as gerosuppressant agent by using an integrated senescence-screening assay. Aging (Albany NY).

[198] Strozyk E., Kulms D. (2013). The role of AKT/mTOR pathway in stress response to UV-irradiation: Implication in skin carcinogenesis by regulation of apoptosis, autophagy and senescence. Int. J. Mol. Sci..

[199] Cao C., Wan Y. (2009). Parameters of protection against ultraviolet radiation-induced skin cell damage. J. Cell Physiol..

[200] Zhang Q.S., Maddock D.A., Chen J.P., Heo S., Chiu C., Lai D., Souza K., Mehta S., Wan Y.S. (2001). Cytokine-induced p38 activation feedback regulates the prolonged activation of AKT cell survival pathway initiated by reactive oxygen species in response to UV irradiation in human keratinocytes. Int. J. Oncol..

[201] Syed D.N., Afaq F., Mukhtar H. (2012). Differential activation of signaling pathways by UVA and UVB radiation in normal human epidermal keratinocytes. Photochem. Photobiol..

[202] Chen Z., Trotman L.C., Shaffer D., Lin H.K., Dotan Z.A., Niki M., Koutcher J.A., Scher H.I., Ludwig T., Gerald W. (2005). Crucial role of p53-dependent cellular senescence in suppression of Pten-deficient tumorigenesis. Nature.

[203] Xu Y., Li N., Xiang R., Sun P. (2014). Emerging roles of the p38 MAPK and PI3K/AKT/mTOR pathways in oncogene-induced senescence. Trends Biochem. Sci..

[204] Zhang B., Fu D., Xu Q., Cong X., Wu C., Zhong X., Ma Y., Lv Z., Chen F., Han L. (2018). The senescence-associated secretory phenotype is potentiated by feedforward regulatory mechanisms involving Zscan4 and TAK1. Nat. Commun..

[205] Atif F., Yousuf S., Espinosa-Garcia C., Sergeeva E., Stein D.G. (2019). Progesterone treatment attenuates glycolytic metabolism and induces senescence in glioblastoma. Sci. Rep..

[206] Liu X.L., Liu J.L., Xu Y.C., Zhang X., Wang Y.X., Qing L.H., Guo W., Ding J., Meng L.H. (2019). Membrane metallo-endopeptidase mediates cellular senescence induced by oncogenic PIK3CA(H1047R) accompanied with pro-tumorigenic secretome. Int. J. Cancer.

[207] Wang W., Guo X., Dan H. (2020). alpha2A-adrenergic receptor inhibits the progression of cervical cancer through blocking PI3K/AKT/mTOR pathway. Onco Targets Ther..

[208] Li H., Zhao S., Shen L., Wang P., Liu S., Ma Y., Liang Z., Wang G., Lv J., Qiu W. (2021). E2F2 inhibition induces autophagy via the PI3K/Akt/mTOR pathway in gastric cancer. Aging (Albany NY).

[209] Zhang Z.Y., Lu M., Liu Z.K., Li H., Yong Y.L., Zhang R.Y., Chen Z.N., Bian H. (2020). Rab11a regulates MMP2 expression by activating the PI3K/AKT pathway in human hepatocellular carcinoma cells. Pathol. Res. Pract..

[210] Cheng C.Y., Hsieh H.L., Hsiao L.D., Yang C.M. (2012). PI3-K/Akt/JNK/NF-kappaB is essential for MMP-9 expression and outgrowth in human limbal epithelial cells on intact amniotic membrane. Stem Cell Res..

[211] Ma Z., Lou S., Jiang Z. (2020). PHLDA2 regulates EMT and autophagy in colorectal cancer via the PI3K/AKT signaling pathway. Aging (Albany NY).

[212] Fasano C., Disciglio V., Bertora S., Lepore Signorile M., Simone C. (2019). FOXO3a from the nucleus to the mitochondria: A round trip in cellular stress response. Cells.

[213] Shin J.M., Jeong Y.J., Cho H.J., Magae J., Bae Y.S., Chang Y.C. (2016). Suppression of c-Myc induces apoptosis via an AMPK/mTOR-dependent pathway by 4-O-methyl-ascochlorin in leukemia cells. Apoptosis.

[214] Ling N.X.Y., Kaczmarek A., Hoque A., Davie E., Ngoei K.R.W., Morrison K.R., Smiles W.J., Forte G.M., Wang T., Lie S. (2020). mTORC1 directly inhibits AMPK to promote cell proliferation under nutrient stress. Nat. Metab..

[215] Kazyken D., Magnuson B., Bodur C., Acosta-Jaquez H.A., Zhang D., Tong X., Barnes T.M., Steinl G.K., Patterson N.E., Altheim C.H. (2019). AMPK directly activates mTORC2 to promote cell survival during acute energetic stress. Sci. Signal..

[216] Shi M., Hu Z., Zhang X., You Q., Wang W., Yan R., Zhu Z. (2020). AMPK activation suppresses mTOR/S6K1 phosphorylation and induces leucine resistance in rats with sepsis. Cell Biol. Int..

[217] Li Y., Xu S., Mihaylova M.M., Zheng B., Hou X., Jiang B., Park O., Luo Z., Lefai E., Shyy J.Y. (2011). AMPK phosphorylates and inhibits SREBP activity to attenuate hepatic steatosis and atherosclerosis in diet-induced insulin-resistant mice. Cell Metab..

[218] Li H., Satriano J., Thomas J.L., Miyamoto S., Sharma K., Pastor-Soler N.M., Hallows K.R., Singh P. (2015). Interactions between HIF-1alpha and AMPK in the regulation of cellular hypoxia adaptation in chronic kidney disease. Am. J. Physiol. Renal. Physiol..

[219] Xiong S., Salazar G., Patrushev N., Alexander R.W. (2011). FoxO1 mediates an autofeedback loop regulating SIRT1 expression. J. Biol. Chem..

[220] Yuan J., Minter-Dykhouse K., Lou Z. (2009). A c-Myc-SIRT1 feedback loop regulates cell growth and transformation. J. Cell Biol..

[221] Ghosh H.S., McBurney M., Robbins P.D. (2010). SIRT1 negatively regulates the mammalian target of rapamycin. PLoS ONE.

[222] Wang R.H., Kim H.S., Xiao C., Xu X., Gavrilova O., Deng C.X. (2011). Hepatic Sirt1 deficiency in mice impairs mTorc2/Akt signaling and results in hyperglycemia, oxidative damage, and insulin resistance. J. Clin. Investig..

[223] Huang J., Gan Q., Han L., Li J., Zhang H., Sun Y., Zhang Z., Tong T. (2008). SIRT1 overexpression antagonizes cellular senescence with activated ERK/S6k1 signaling in human diploid fibroblasts. PLoS ONE.

[224] Ponugoti B., Kim D.H., Xiao Z., Smith Z., Miao J., Zang M., Wu S.Y., Chiang C.M., Veenstra T.D., Kemper J.K. (2010). SIRT1 deacetylates and inhibits SREBP-1C activity in regulation of hepatic lipid metabolism. J. Biol. Chem..

[225] Ryu D.R., Yu M.R., Kong K.H., Kim H., Kwon S.H., Jeon J.S., Han D.C., Noh H. (2019). Sirt1-hypoxia-inducible factor-1alpha interaction is a key mediator of tubulointerstitial damage in the aged kidney. Aging Cell.

[226] Li X.H., Chen C., Tu Y., Sun H.T., Zhao M.L., Cheng S.X., Qu Y., Zhang S. (2013). Sirt1 promotes axonogenesis by deacetylation of Akt and inactivation of GSK3. Mol. Neurobiol..

[227] Roy S.K., Srivastava R.K., Shankar S. (2010). Inhibition of PI3K/AKT and MAPK/ERK pathways causes activation of FOXO transcription factor, leading to cell cycle arrest and apoptosis in pancreatic cancer. J. Mol. Signal..

[228] Pan C.W., Jin X., Zhao Y., Pan Y., Yang J., Karnes R.J., Zhang J., Wang L., Huang H. (2017). AKT-phosphorylated FOXO1 suppresses ERK activation and chemoresistance by disrupting IQGAP1-MAPK interaction. EMBO J..

[229] Pathria G., Verma S., Yin J., Scott D.A., Ronai Z.A. (2021). MAPK signaling regulates c-MYC for melanoma cell adaptation to asparagine restriction. EMBO Rep..

[230] Carracedo A., Ma L., Teruya-Feldstein J., Rojo F., Salmena L., Alimonti A., Egia A., Sasaki A.T., Thomas G., Kozma S.C. (2008). Inhibition of mTORC1 leads to MAPK pathway activation through a PI3K-dependent feedback loop in human cancer. J. Clin. Investig..

[231] Benavides-Serrato A., Anderson L., Holmes B., Cloninger C., Artinian N., Bashir T., Gera J. (2014). mTORC2 modulates feedback regulation of p38 MAPK activity via DUSP10/MKP5 to confer differential responses to PP242 in glioblastoma. Genes Cancer.

[232] Roth G., Kotzka J., Kremer L., Lehr S., Lohaus C., Meyer H.E., Krone W., Muller-Wieland D. (2000). MAP kinases Erk1/2 phosphorylate sterol regulatory element-binding protein (SREBP)-1a at serine 117 in vitro. J. Biol. Chem..

[233] Qin X., Jiang B., Zhang Y. (2016). 4E-BP1, a multifactor regulated multifunctional protein. Cell Cycle.

[234] Wang Q., Zhou Y., Wang X., Evers B.M. (2006). Glycogen synthase kinase-3 is a negative regulator of extracellular signal-regulated kinase. Oncogene.

[235] Brunet A., Sweeney L.B., Sturgill J.F., Chua K.F., Greer P.L., Lin Y., Tran H., Ross S.E., Mostoslavsky R., Cohen H.Y. (2004). Stress-dependent regulation of FOXO transcription factors by the SIRT1 deacetylase. Science.

[236] Huang H., Tindall D.J. (2007). Dynamic FoxO transcription factors. J. Cell Sci..

[237] Das T.P., Suman S., Alatassi H., Ankem M.K., Damodaran C. (2016). Inhibition of AKT promotes FOXO3a-dependent apoptosis in prostate cancer. Cell Death Dis..

[238] Farhan M., Silva M., Li S., Yan F., Fang J., Peng T., Hu J., Tsao M.S., Little P., Zheng W. (2020). The role of FOXOs and autophagy in cancer and metastasis-Implications in therapeutic development. Med. Res. Rev..

[239] Mammucari C., Milan G., Romanello V., Masiero E., Rudolf R., Del Piccolo P., Burden S.J., Di Lisi R., Sandri C., Zhao J. (2007). FoxO3 controls autophagy in skeletal muscle in vivo. Cell Metab..

[240] Hariharan N., Maejima Y., Nakae J., Paik J., Depinho R.A., Sadoshima J. (2010). Deacetylation of FoxO by Sirt1 plays an essential role in mediating starvation-induced autophagy in cardiac myocytes. Circ Res..

[241] Vallejo-Gracia A., Chen I.P., Perrone R., Besnard E., Boehm D., Battivelli E., Tezil T., Krey K., Raymond K.A., Hull P.A. (2020). FOXO1 promotes HIV latency by suppressing ER stress in T cells. Nat Microbiol.

[242] Gupta A., Stocker H. (2020). FoxO suppresses endoplasmic reticulum stress to inhibit growth of Tsc1-deficient tissues under nutrient restriction. eLife.

[243] Zhou L., Li R., Liu C., Sun T., Htet Aung L.H., Chen C., Gao J., Zhao Y., Wang K. (2017). Foxo3a inhibits mitochondrial fission and protects against doxorubicin-induced cardiotoxicity by suppressing MIEF2. Free Radic. Biol. Med..

[244] Pan J., Zhang X., Fang X., Xin Z. (2021). Construction on of a ferroptosis-related lncRNA-based model to improve the prognostic evaluation of gastric cancer patients based on bioinformatics. Front. Genet..

[245] SONG W. (2018). Platycodin D induces necroptosis of prostate cancer PC-3 cells through FOXO3a pathway. Tumor.

[246] Ju Y., Xu T., Zhang H., Yu A. (2014). FOXO1-dependent DNA damage repair is regulated by JNK in lung cancer cells. Int. J. Oncol..

[247] White R.R., Maslov A.Y., Lee M., Wilner S.E., Levy M., Vijg J. (2020). FOXO3a acts to suppress DNA double-strand break-induced mutations. Aging Cell.

[248] Kyoung Kim H., Kyoung Kim Y., Song I.H., Baek S.H., Lee S.R., Hye Kim J., Kim J.R. (2005). Down-regulation of a forkhead transcription factor, FOXO3a, accelerates cellular senescence in human dermal fibroblasts. J. Gerontol. A Biol. Sci. Med Sci..

[249] Delpoux A., Marcel N., Hess Michelini R., Katayama C.D., Allison K.A., Glass C.K., Quinones-Parra S.M., Murre C., Loh L., Kedzierska K. (2021). FOXO1 constrains activation and regulates senescence in CD8 T cells. Cell Rep..

[250] Ma Z., Xin Z., Hu W., Jiang S., Yang Z., Yan X., Li X., Yang Y., Chen F. (2018). Forkhead box O proteins: Crucial regulators of cancer EMT. Semin. Cancer Biol..

[251] Belguise K., Guo S., Sonenshein G.E. (2007). Activation of FOXO3a by the green tea polyphenol epigallocatechin-3-gallate induces estrogen receptor alpha expression reversing invasive phenotype of breast cancer cells. Cancer Res..

[252] Hill R., Kalathur R.K., Callejas S., Colaco L., Brandao R., Serelde B., Cebria A., Blanco-Aparicio C., Pastor J., Futschik M. (2014). A novel phosphatidylinositol 3-kinase (PI3K) inhibitor directs a potent FOXO-dependent, p53-independent cell cycle arrest phenotype characterized by the differential induction of a subset of FOXO-regulated genes. Breast Cancer Res..

[253] Hoffman B., Liebermann D.A. (2008). Apoptotic signaling by c-MYC. Oncogene.

[254] Toh P.P., Luo S., Menzies F.M., Rasko T., Wanker E.E., Rubinsztein D.C. (2013). Myc inhibition impairs autophagosome formation. Hum. Mol. Genet..

[255] Zhang Q. (2018). Stressing Myc-driven cancer out. Sci. Transl. Med..

[256] Carugo A., Minelli R., Sapio L., Soeung M., Carbone F., Robinson F.S., Tepper J., Chen Z., Lovisa S., Svelto M. (2019). p53 is a master regulator of proteostasis in SMARCB1-deficient malignant rhabdoid tumors. Cancer Cell.

[257] Badrinath N., Yoo S.Y. (2018). Mitochondria in cancer: In the aspects of tumorigenesis and targeted therapy. Carcinogenesis.

[258] Hao S., Yu J., He W., Huang Q., Zhao Y., Liang B., Zhang S., Wen Z., Dong S., Rao J. (2017). Cysteine dioxygenase 1 mediates erastin-induced ferroptosis in human gastric cancer cells. Neoplasia.

[259] Mou Y., Wang J., Wu J., He D., Zhang C., Duan C., Li B. (2019). Ferroptosis, a new form of cell death: Opportunities and challenges in cancer. J. Hematol. Oncol..

[260] Seong D., Jeong M., Seo J., Lee J.Y., Hwang C.H., Shin H.C., Shin J.Y., Nam Y.W., Jo J.Y., Lee H. (2020). Identification of MYC as an antinecroptotic protein that stifles RIPK1-RIPK3 complex formation. Proc. Natl. Acad. Sci. USA.

[261] Vafa O., Wade M., Kern S., Beeche M., Pandita T.K., Hampton G.M., Wahl G.M. (2002). c-Myc can induce DNA damage, increase reactive oxygen species, and mitigate p53 function: A mechanism for oncogene-induced genetic instability. Molecular. Cell.

[262] Jin Z., May W.S., Gao F., Flagg T., Deng X. (2006). Bcl2 suppresses DNA repair by enhancing c-Myc transcriptional activity. J. Biol. Chem..

[263] Wu C.H., van Riggelen J., Yetil A., Fan A.C., Bachireddy P., Felsher D.W. (2007). Cellular senescence is an important mechanism of tumor regression upon c-Myc inactivation. Proc. Natl. Acad. Sci. USA.

[264] Zhao Y., Jian W., Gao W., Zheng Y.X., Wang Y.K., Zhou Z.Q., Zhang H., Wang C.J. (2013). RNAi silencing of c-Myc inhibits cell migration, invasion, and proliferation in HepG2 human hepatocellular carcinoma cell line: C-Myc silencing in hepatocellular carcinoma cell. Cancer Cell Int..

[265] Hsin I.L., Shen H.P., Chang H.Y., Ko J.L., Wang P.H. (2021). Suppression of PI3K/Akt/mTOR/c-Myc/mtp53 positive feedback loop induces cell cycle arrest by dual PI3K/mTOR inhibitor PQR309 in endometrial cancer cell lines. Cells.

[266] Villar V.H., Nguyen T.L., Delcroix V., Teres S., Bouchecareilh M., Salin B., Bodineau C., Vacher P., Priault M., Soubeyran P. (2017). mTORC1 inhibition in cancer cells protects from glutaminolysis-mediated apoptosis during nutrient limitation. Nat. Commun..

[267] He K., Zheng X., Li M., Zhang L., Yu J. (2016). mTOR inhibitors induce apoptosis in colon cancer cells via CHOP-dependent DR5 induction on 4E-BP1 dephosphorylation. Oncogene.

[268] Li H., Lin J., Wang X., Yao G., Wang L., Zheng H., Yang C., Jia C., Liu A., Bai X. (2012). Targeting of mTORC2 prevents cell migration and promotes apoptosis in breast cancer. Breast Cancer Res. Treat..

[269] Kim Y.M., Park J.M., Grunwald D., Kim D.H. (2016). An expanded role for mTORC1 in autophagy. Mol. Cell Oncol..

[270] Puustinen P., Rytter A., Mortensen M., Kohonen P., Moreira J.M., Jaattela M. (2014). CIP2A oncoprotein controls cell growth and autophagy through mTORC1 activation. J. Cell Biol..

[271] Paquette M., El-Houjeiri L., Pause A. (2018). mTOR pathways in cancer and autophagy. Cancers.

[272] de la Cruz Lopez K.G., Toledo Guzman M.E., Sanchez E.O., Garcia Carranca A. (2019). mTORC1 as a regulator of mitochondrial functions and a therapeutic target in cancer. Front. Oncol..

[273] Yi J., Zhu J., Wu J., Thompson C.B., Jiang X. (2020). Oncogenic activation of PI3K-AKT-mTOR signaling suppresses ferroptosis via SREBP-mediated lipogenesis. Proc. Natl. Acad. Sci. USA.

[274] Zhang Y., Swanda R.V., Nie L., Liu X., Wang C., Lee H., Lei G., Mao C., Koppula P., Cheng W. (2021). mTORC1 couples cyst(e)ine availability with GPX4 protein synthesis and ferroptosis regulation. Nat. Commun..

[275] Abe K., Yano T., Tanno M., Miki T., Kuno A., Sato T., Kouzu H., Nakata K., Ohwada W., Kimura Y. (2019). mTORC1 inhibition attenuates necroptosis through RIP1 inhibition-mediated TFEB activation. Biochim. Biophys. Acta Mol. Basis Dis..

[276] Ma Y., Vassetzky Y., Dokudovskaya S. (2018). mTORC1 pathway in DNA damage response. Biochim. Biophys. Acta Mol. Cell Res..

[277] Shin S., Walker K.A., Yoon S.O. (2022). The PIKK-AKT connection in the DNA damage response. Sci. Signal..

[278] Chen A., Jin J., Cheng S., Liu Z., Yang C., Chen Q., Liang W., Li K., Kang D., Ouyang Z. (2022). mTORC1 induces plasma membrane depolarization and promotes preosteoblast senescence by regulating the sodium channel Scn1a. Bone Res..

[279] Yang H.W., Hong H.L., Luo W.W., Dai C.M., Chen X.Y., Wang L.P., Li Q., Li Z.Q., Liu P.Q., Li Z.M. (2018). mTORC2 facilitates endothelial cell senescence by suppressing Nrf2 expression via the Akt/GSK-3beta/C/EBPalpha signaling pathway. Acta Pharmacol. Sin..

[280] Yan T., Zhang J., Tang D., Zhang X., Jiang X., Zhao L., Zhang Q., Zhang D., Huang Y. (2017). Hypoxia regulates mTORC1-mediated keratinocyte motility and migration via the AMPK pathway. PLoS ONE.

[281] Sridharan S., Basu A. (2011). S6 kinase 2 promotes breast cancer cell survival via Akt. Cancer Res..

[282] Nam K.H., Yi S.A., Nam G., Noh J.S., Park J.W., Lee M.G., Park J.H., Oh H., Lee J., Lee K.R. (2019). Identification of a novel S6K1 inhibitor, rosmarinic acid methyl ester, for treating cisplatin-resistant cervical cancer. BMC Cancer.

[283] Pardo V., Gonzalez-Rodriguez A., Muntane J., Kozma S.C., Valverde A.M. (2015). Role of hepatocyte S6K1 in palmitic acid-induced endoplasmic reticulum stress, lipotoxicity, insulin resistance and in oleic acid-induced protection. Food Chem. Toxicol..

[284] Tran Q., Jung J.H., Park J., Lee H., Hong Y., Cho H., Kim M., Park S., Kwon S.H., Kim S.H. (2018). S6 kinase 1 plays a key role in mitochondrial morphology and cellular energy flow. Cell Signal..

[285] Chen H.-Y., Goldman A.R., Zayas-Bazan D., Reyes-Uribe P.I., Guterres A.N., Lipchick B., Basu S., Yin X., Axelrod M.J., Lu Y. (2021). Selective abrogation of S6K2 maps lipid homeostasis as a survival vulnerability in MAPKi-resistant NRAS^MUT^ melanoma. bioRxiv.

[286] Lai K.P., Leong W.F., Chau J.F., Jia D., Zeng L., Liu H., He L., Hao A., Zhang H., Meek D. (2010). S6K1 is a multifaceted regulator of Mdm2 that connects nutrient status and DNA damage response. EMBO J..

[287] Amar-Schwartz A., Ben-Hur V., Jbara A., Cohen Y., Barnabas G., Siegfried Z., Mashahreh B., Hassouna F., Shilo A., Abu-Odeh M. (2022). S6K1 phosphorylates Cdk1 and MSH6 to regulate DNA repair. bioRxiv.

[288] Rajapakse A.G., Yepuri G., Carvas J.M., Stein S., Matter C.M., Scerri I., Ruffieux J., Montani J.P., Ming X.F., Yang Z. (2011). Hyperactive S6K1 mediates oxidative stress and endothelial dysfunction in aging: Inhibition by resveratrol. PLoS ONE.

[289] Barilari M., Bonfils G., Treins C., Koka V., De Villeneuve D., Fabrega S., Pende M. (2017). ZRF1 is a novel S6 kinase substrate that drives the senescence programme. EMBO J..

[290] Zhang J., Guo J., Qin X., Wang B., Zhang L., Wang Y., Gan W., Pandolfi P.P., Chen W., Wei W. (2018). The p85 isoform of the kinase S6K1 functions as a secreted oncoprotein to facilitate cell migration and tumor growth. Sci. Signal..

[291] Li Y., Yu P., Long J., Tang L., Zhang X., Zhou Z., Cao D., Su J., Chen X., Peng C. (2022). A novel ribosomal protein S6 kinase 2 inhibitor attenuates the malignant phenotype of cutaneous malignant melanoma cells by inducing cell cycle arrest and apoptosis. Bioengineered.

[292] Sun Y., He W., Luo M., Zhou Y., Chang G., Ren W., Wu K., Li X., Shen J., Zhao X. (2015). SREBP1 regulates tumorigenesis and prognosis of pancreatic cancer through targeting lipid metabolism. Tumour Biol..

[293] Zhou C., Qian W., Li J., Ma J., Chen X., Jiang Z., Cheng L., Duan W., Wang Z., Wu Z. (2019). High glucose microenvironment accelerates tumor growth via SREBP1-autophagy axis in pancreatic cancer. J. Exp. Clin. Cancer Res..

[294] Griffiths B., Lewis C.A., Bensaad K., Ros S., Zhang Q., Ferber E.C., Konisti S., Peck B., Miess H., East P. (2013). Sterol regulatory element binding protein-dependent regulation of lipid synthesis supports cell survival and tumor growth. Cancer Metab..

[295] Nakagawa H., Umemura A., Taniguchi K., Font-Burgada J., Dhar D., Ogata H., Zhong Z., Valasek M.A., Seki E., Hidalgo J. (2014). ER stress cooperates with hypernutrition to trigger TNF-dependent spontaneous HCC development. Cancer Cell.

[296] Wu D., Yang Y., Hou Y., Zhao Z., Liang N., Yuan P., Yang T., Xing J., Li J. (2022). Increased mitochondrial fission drives the reprogramming of fatty acid metabolism in hepatocellular carcinoma cells through suppression of Sirtuin 1. Cancer Commun..

[297] Lu D., Parisi L., Gokcumen O., Atilla E. SREBP1 activation contributes to fatty acid accumulations in necroptosis. bioRxiv.

[298] Yang B., Zhang B., Cao Z., Xu X., Huo Z., Zhang P., Xiang S., Zhao Z., Lv C., Meng M. (2020). The lipogenic LXR-SREBF1 signaling pathway controls cancer cell DNA repair and apoptosis and is a vulnerable point of malignant tumors for cancer therapy. Cell Death Differ..

[299] Kim Y.M., Shin H.T., Seo Y.H., Byun H.O., Yoon S.H., Lee I.K., Hyun D.H., Chung H.Y., Yoon G. (2010). Sterol regulatory element-binding protein (SREBP)-1-mediated lipogenesis is involved in cell senescence. J. Biol. Chem..

[300] Bao J., Zhu L., Zhu Q., Su J., Liu M., Huang W. (2016). SREBP-1 is an independent prognostic marker and promotes invasion and migration in breast cancer. Oncol. Lett..

[301] Bengoechea-Alonso M.T., Punga T., Ericsson J. (2005). Hyperphosphorylation regulates the activity of SREBP1 during mitosis. Proc. Natl. Acad. Sci. USA.

[302] Dumstorf C.A., Konicek B.W., McNulty A.M., Parsons S.H., Furic L., Sonenberg N., Graff J.R. (2010). Modulation of 4E-BP1 function as a critical determinant of enzastaurin-induced apoptosis. Mol. Cancer Ther..

[303] Lai C.Y., Pan S.L., Yang X.M., Chang L.H., Chang Y.L., Yang P.C., Lee K.H., Teng C.M. (2013). Depletion of 4E-BP1 and regulation of autophagy lead to YXM110-induced anticancer effects. Carcinogenesis.

[304] Yu H.C., Hou D.R., Liu C.Y., Lin C.S., Shiau C.W., Cheng A.L., Chen K.F. (2013). Cancerous inhibitor of protein phosphatase 2A mediates bortezomib-induced autophagy in hepatocellular carcinoma independent of proteasome. PLoS ONE.

[305] Morita M., Prudent J., Basu K., Goyon V., Katsumura S., Hulea L., Pearl D., Siddiqui N., Strack S., McGuirk S. (2017). mTOR controls mitochondrial dynamics and cell survival via MTFP1. Mol. Cell.

[306] Yangyun W., Guowei S., Shufen S., Jie Y., Rui Y., Yu R. (2022). Everolimus accelerates Erastin and RSL3-induced ferroptosis in renal cell carcinoma. Gene.

[307] Wang Z., Feng J., Yu J., Chen G. (2019). FKBP12 mediates necroptosis by initiating RIPK1-RIPK3-MLKL signal transduction in response to TNF receptor 1 ligation. J. Cell Sci..

[308] Muller D., Shin S., Goullet de Rugy T., Samain R., Baer R., Strehaiano M., Masvidal-Sanz L., Guillermet-Guibert J., Jean C., Tsukumo Y. (2019). eIF4A inhibition circumvents uncontrolled DNA replication mediated by 4E-BP1 loss in pancreatic cancer. JCI Insight.

[309] Chao S.K., Horwitz S.B., McDaid H.M. (2011). Insights into 4E-BP1 and p53 mediated regulation of accelerated cell senescence. Oncotarget.

[310] Cai W., Ye Q., She Q.B. (2014). Loss of 4E-BP1 function induces EMT and promotes cancer cell migration and invasion via cap-dependent translational activation of snail. Oncotarget.

[311] Dong G., Zhang R., Hu Q., Martin E.M., Qin Y., Lu C., Xia Y., Wang X., Du G. (2022). Prothioconazole induces cell cycle arrest by up-regulation of EIF4EBP1 in extravillous trophoblast cells. Arch Toxicol..

[312] Mace T.A., Collins A.L., Wojcik S.E., Croce C.M., Lesinski G.B., Bloomston M. (2013). Hypoxia induces the overexpression of microRNA-21 in pancreatic cancer cells. J. Surg. Res..

[313] He G., Jiang Y., Zhang B., Wu G. (2014). The effect of HIF-1alpha on glucose metabolism, growth and apoptosis of pancreatic cancerous cells. Asia Pac. J. Clin. Nutr..

[314] Zhu H., Wang D., Zhang L., Xie X., Wu Y., Liu Y., Shao G., Su Z. (2014). Upregulation of autophagy by hypoxia-inducible factor-1alpha promotes EMT and metastatic ability of CD133+ pancreatic cancer stem-like cells during intermittent hypoxia. Oncol. Rep..

[315] Lee B.R., Chang S.Y., Hong E.H., Kwon B.E., Kim H.M., Kim Y.J., Lee J., Cho H.J., Cheon J.H., Ko H.J. (2014). Elevated endoplasmic reticulum stress reinforced immunosuppression in the tumor microenvironment via myeloid-derived suppressor cells. Oncotarget.

[316] Marsboom G., Toth P.T., Ryan J.J., Hong Z., Wu X., Fang Y.H., Thenappan T., Piao L., Zhang H.J., Pogoriler J. (2012). Dynamin-related protein 1-mediated mitochondrial mitotic fission permits hyperproliferation of vascular smooth muscle cells and offers a novel therapeutic target in pulmonary hypertension. Circ. Res..

[317] Madhu V., Boneski P.K., Silagi E., Qiu Y., Kurland I., Guntur A.R., Shapiro I.M., Risbud M.V. (2020). Hypoxic regulation of mitochondrial metabolism and mitophagy in nucleus pulposus cells is dependent on HIF-1alpha-BNIP3 axis. J. Bone Miner Res..

[318] Lin Z., Song J., Gao Y., Huang S., Dou R., Zhong P., Huang G., Han L., Zheng J., Zhang X. (2022). Hypoxia-induced HIF-1alpha/lncRNA-PMAN inhibits ferroptosis by promoting the cytoplasmic translocation of ELAVL1 in peritoneal dissemination from gastric cancer. Redox Biol..

[319] Yang X.S., Yi T.L., Zhang S., Xu Z.W., Yu Z.Q., Sun H.T., Yang C., Tu Y., Cheng S.X. (2017). Hypoxia-inducible factor-1 alpha is involved in RIP-induced necroptosis caused by in vitro and in vivo ischemic brain injury. Sci. Rep..

[320] Lou J.J., Chua Y.L., Chew E.H., Gao J., Bushell M., Hagen T. (2010). Inhibition of hypoxia-inducible factor-1alpha (HIF-1alpha) protein synthesis by DNA damage inducing agents. PLoS ONE.

[321] Kato H., Inoue T., Asanoma K., Nishimura C., Matsuda T., Wake N. (2006). Induction of human endometrial cancer cell senescence through modulation of HIF-1alpha activity by EGLN1. Int. J. Cancer.

[322] Koshiji M., Kageyama Y., Pete E.A., Horikawa I., Barrett J.C., Huang L.E. (2004). HIF-1alpha induces cell cycle arrest by functionally counteracting Myc. EMBO J..

[323] Martelli A.M., Evangelisti C., Paganelli F., Chiarini F., McCubrey J.A. (2021). GSK-3: A multifaceted player in acute leukemias. Leukemia.

[324] Liu L., Chen L., Yang J., Ye C., Yang P., Zhang L., Shen B. (2022). LZTS1 promotes proliferation and suppresses apoptosis by inhibiting the activation of AKT/GSK-3β signaling pathway in pancreatic cancer cells. Trop. J. Pharm. Res..

[325] Pan H.Y., Valapala M. (2022). Regulation of autophagy by the glycogen synthase kinase-3 (GSK-3) signaling pathway. Int. J. Mol. Sci..

[326] Wang L., Li J., Di L.J. (2022). Glycogen synthesis and beyond, a comprehensive review of GSK3 as a key regulator of metabolic pathways and a therapeutic target for treating metabolic diseases. Med. Res. Rev..

[327] Chou C.H., Lin C.C., Yang M.C., Wei C.C., Liao H.D., Lin R.C., Tu W.Y., Kao T.C., Hsu C.M., Cheng J.T. (2012). GSK3beta-mediated Drp1 phosphorylation induced elongated mitochondrial morphology against oxidative stress. PLoS ONE.

[328] Wang L., Ouyang S., Li B., Wu H., Wang F. (2021). GSK-3beta manipulates ferroptosis sensitivity by dominating iron homeostasis. Cell Death Discov..

[329] Ciotti S., Iuliano L., Cefalu S., Comelli M., Mavelli I., Di Giorgio E., Brancolini C. (2020). GSK3beta is a key regulator of the ROS-dependent necrotic death induced by the quinone DMNQ. Cell Death Dis..

[330] Grassilli E., Ianzano L., Bonomo S., Missaglia C., Cerrito M.G., Giovannoni R., Masiero L., Lavitrano M. (2014). GSK3A is redundant with GSK3B in modulating drug resistance and chemotherapy-induced necroptosis. PLoS ONE.

[331] Kim Y.M., Song I., Seo Y.H., Yoon G. (2013). Glycogen synthase kinase 3 inactivation induces cell senescence through sterol regulatory element binding protein 1-mediated lipogenesis in Chang cells. Endocrinol. Metab. (Seoul).

[332] Witzig T.E., Reeder C., Han J.J., LaPlant B., Stenson M., Tun H.W., Macon W., Ansell S.M., Habermann T.M., Inwards D.J. (2015). The mTORC1 inhibitor everolimus has antitumor activity *in vitro* and produces tumor responses in patients with relapsed T-cell lymphoma. Blood.

[333] Sridharan S., Basu A. (2020). Distinct roles of mTOR targets S6K1 and S6K2 in breast cancer. Int. J. Mol. Sci..

[334] Zhao J., Brault J.J., Schild A., Cao P., Sandri M., Schiaffino S., Lecker S.H., Goldberg A.L. (2007). FoxO3 coordinately activates protein degradation by the autophagic/lysosomal and proteasomal pathways in atrophying muscle cells. Cell Metab..

[335] Gonzalez-Quiroz M., Urra H., Limia C.M., Hetz C. (2018). Homeostatic interplay between FoxO proteins and ER proteostasis in cancer and other diseases. Semin. Cancer Biol..

[336] Chen W., Jia Z., Pan M.H., Anandh Babu P.V. (2016). Natural products for the prevention of oxidative stress-related diseases: Mechanisms and strategies. Oxidative Med. Cell. Longev..

[337] Yang L., Chen Y., Liu Y., Xing Y., Miao C., Zhao Y., Chang X., Zhang Q. (2020). The role of oxidative stress and natural antioxidants in ovarian aging. Front. Pharmacol..

[338] Cicero N., Gangemi S., Allegra A. (2022). Natural products and oxidative stress: Potential agents against multiple myeloma. Nat. Prod. Res..

[339] Peng S.Y., Lin L.C., Chen S.R., Farooqi A.A., Cheng Y.B., Tang J.Y., Chang H.W. (2021). Pomegranate extract (POMx) induces mitochondrial dysfunction and apoptosis of oral cancer cells. Antioxidants.

[340] Peng D., Zaika A., Que J., El-Rifai W. (2021). The antioxidant response in Barrett’s tumorigenesis: A double-edged sword. Redox Biol..

[341] Bouayed J., Bohn T. (2010). Exogenous antioxidants--Double-edged swords in cellular redox state: Health beneficial effects at physiologic doses versus deleterious effects at high doses. Oxidative Med. Cell. Longev..

[342] Tang J.Y., Ou-Yang F., Hou M.F., Huang H.W., Wang H.R., Li K.T., Fayyaz S., Shu C.W., Chang H.W. (2019). Oxidative stress-modulating drugs have preferential anticancer effects—Involving the regulation of apoptosis, DNA damage, endoplasmic reticulum stress, autophagy, metabolism, and migration. Semin. Cancer Biol..

[343] Chang H.S., Tang J.Y., Yen C.Y., Huang H.W., Wu C.Y., Chung Y.A., Wang H.R., Chen I.S., Huang M.Y., Chang H.W. (2016). Antiproliferation of *Cryptocarya concinna*-derived cryptocaryone against oral cancer cells involving apoptosis, oxidative stress, and DNA damage. BMC Complement. Altern. Med..

[344] Yao J., Duan D., Song Z.L., Zhang J., Fang J. (2020). Sanguinarine as a new chemical entity of thioredoxin reductase inhibitor to elicit oxidative stress and promote tumor cell apoptosis. Free Radic. Biol. Med..

[345] Wu C.F., Lee M.G., El-Shazly M., Lai K.H., Ke S.C., Su C.W., Shih S.P., Sung P.J., Hong M.C., Wen Z.H. (2018). Isoaaptamine induces T-47D cells apoptosis and autophagy via oxidative stress. Mar. Drugs.

[346] Poornima P., Weng C.F., Padma V.V. (2013). Neferine from *Nelumbo nucifera* induces autophagy through the inhibition of PI3K/Akt/mTOR pathway and ROS hyper generation in A549 cells. Food Chem..

[347] Wang Y., Wang J.W., Xiao X., Shan Y., Xue B., Jiang G., He Q., Chen J., Xu H.G., Zhao R.X. (2013). Piperlongumine induces autophagy by targeting p38 signaling. Cell Death Dis..

[348] Shen S., Zhang Y., Zhang R., Gong X. (2013). Sarsasapogenin induces apoptosis via the reactive oxygen species-mediated mitochondrial pathway and ER stress pathway in HeLa cells. Biochem. Biophys. Res. Commun..

[349] Yao X., Jing X., Guo J., Sun K., Deng Y., Zhang Y., Guo F., Ye Y. (2019). Icariin protects bone marrow mesenchymal stem cells against iron overload induced dysfunction through mitochondrial fusion and fission, PI3K/AKT/mTOR and MAPK pathways. Front. Pharmacol..

[350] Yang J., Guo W., Wang J., Yang X., Zhang Z., Zhao Z. (2020). T-2 toxin-induced oxidative stress leads to imbalance of mitochondrial fission and fusion to activate cellular apoptosis in the human liver 7702 cell line. Toxins.

[351] Tang Q., Zheng G., Feng Z., Chen Y., Lou Y., Wang C., Zhang X., Zhang Y., Xu H., Shang P. (2017). Trehalose ameliorates oxidative stress-mediated mitochondrial dysfunction and ER stress via selective autophagy stimulation and autophagic flux restoration in osteoarthritis development. Cell Death Dis..

[352] Shao C., Yuan J., Liu Y., Qin Y., Wang X., Gu J., Chen G., Zhang B., Liu H.K., Zhao J. (2020). Epileptic brain fluorescent imaging reveals apigenin can relieve the myeloperoxidase-mediated oxidative stress and inhibit ferroptosis. Proc. Natl. Acad. Sci. USA.

[353] Wei R., Zhao Y., Wang J., Yang X., Li S., Wang Y., Yang X., Fei J., Hao X., Zhao Y. (2021). Tagitinin C induces ferroptosis through PERK-Nrf2-HO-1 signaling pathway in colorectal cancer cells. Int. J. Biol. Sci..

[354] Jia Y., Wang F., Guo Q., Li M., Wang L., Zhang Z., Jiang S., Jin H., Chen A., Tan S. (2018). Curcumol induces RIPK1/RIPK3 complex-dependent necroptosis via JNK1/2-ROS signaling in hepatic stellate cells. Redox Biol..

[355] Liu T., Sun X., Cao Z. (2019). Shikonin-induced necroptosis in nasopharyngeal carcinoma cells via ROS overproduction and upregulation of RIPK1/RIPK3/MLKL expression. Onco Targets Ther..

[356] Chang Y.T., Huang C.Y., Li K.T., Li R.N., Liaw C.C., Wu S.H., Liu J.R., Sheu J.H., Chang H.W. (2016). Sinuleptolide inhibits proliferation of oral cancer Ca9-22 cells involving apoptosis, oxidative stress, and DNA damage. Arch Oral. Biol..

[357] Li B.S., Zhu R.Z., Lim S.H., Seo J.H., Choi B.M. (2021). Apigenin alleviates oxidative stress-induced cellular senescence via modulation of the SIRT1-NAD^+^-CD38 Axis. Am. J. Chin. Med..

[358] Yu T.J., Tang J.Y., Shiau J.P., Hou M.F., Yen C.H., Ou-Yang F., Chen C.Y., Chang H.W. (2022). Gingerenone A induces antiproliferation and senescence of breast cancer cells. Antioxidants.

[359] Ketola K., Hilvo M., Hyotylainen T., Vuoristo A., Ruskeepaa A.L., Oresic M., Kallioniemi O., Iljin K. (2012). Salinomycin inhibits prostate cancer growth and migration via induction of oxidative stress. Br. J. Cancer.

[360] Yu T.J., Tang J.Y., Ou-Yang F., Wang Y.Y., Yuan S.F., Tseng K., Lin L.C., Chang H.W. (2020). Low concentration of withaferin A inhibits oxidative stress-mediated migration and invasion in oral cancer cells. Biomolecules.

[361] Palozza P., Simone R., Catalano A., Boninsegna A., Bohm V., Frohlich K., Mele M.C., Monego G., Ranelletti F.O. (2010). Lycopene prevents 7-ketocholesterol-induced oxidative stress, cell cycle arrest and apoptosis in human macrophages. J. Nutr. Biochem..

[362] Chen C.R., Zhang J., Wu K.W., Liu P.Y., Wang S.J., Chen D.Y., Ji Z.N. (2015). Gracillin induces apoptosis in HL60 human leukemic cell line via oxidative stress and cell cycle arrest of G1. Pharmazie.

[363] Yang X.J., Miao F., Yao Y., Cao F.J., Yang R., Ma Y.N., Qin B.F., Zhou L. (2012). *In vitro* antifungal activity of sanguinarine and chelerythrine derivatives against phytopathogenic fungi. Molecules.

[364] Oettinghaus B., D’Alonzo D., Barbieri E., Restelli L.M., Savoia C., Licci M., Tolnay M., Frank S., Scorrano L. (2016). DRP1-dependent apoptotic mitochondrial fission occurs independently of BAX, BAK and APAF1 to amplify cell death by BID and oxidative stress. Biochim. Biophys. Acta.

[365] Schmidt C., Loos C., Jin L., Schmiech M., Schmidt C.Q., Gaafary M.E., Syrovets T., Simmet T. (2017). Acetyl-lupeolic acid inhibits Akt signaling and induces apoptosis in chemoresistant prostate cancer cells in vitro and in vivo. Oncotarget.

[366] Xu K., Guo C., Shi D., Meng J., Tian H., Guo S. (2019). Discovery of natural dimeric naphthopyrones as potential cytotoxic agents through ROS-mediated apoptotic pathway. Mar. Drugs.

[367] Pai J.T., Hsu M.W., Leu Y.L., Chang K.T., Weng M.S. (2021). Induction of G2/M cell cycle arrest via p38/p21(Waf1/Cip1)-dependent signaling pathway activation by bavachinin in non-small-cell lung cancer cells. Molecules.

[368] Pramanik K.C., Kudugunti S.K., Fofaria N.M., Moridani M.Y., Srivastava S.K. (2013). Caffeic acid phenethyl ester suppresses melanoma tumor growth by inhibiting PI3K/AKT/XIAP pathway. Carcinogenesis.

[369] Qi Y., Chen L., Zhang L., Liu W.B., Chen X.Y., Yang X.G. (2013). Crocin prevents retinal ischaemia/reperfusion injury-induced apoptosis in retinal ganglion cells through the PI3K/AKT signalling pathway. Exp. Eye Res..

[370] Chen X.L., Fu J.P., Shi J., Wan P., Cao H., Tang Z.M. (2015). CXC195 induces apoptosis and endoplastic reticulum stress in human hepatocellular carcinoma cells by inhibiting the PI3K/Akt/mTOR signaling pathway. Mol. Med. Rep..

[371] Luo T., Li Z., Deng X.M., Jiang K., Liu D., Zhang H.H., Shi T., Liu L.Y., Wen H.X., Li Q.E. (2022). Isolation, synthesis and bioactivity evaluation of isoquinoline alkaloids from *Corydalis hendersonii* Hemsl. against gastric cancer in vitro and in vivo. Bioorg. Med. Chem..

[372] Xiao D., Singh S.V. (2006). Diallyl trisulfide, a constituent of processed garlic, inactivates Akt to trigger mitochondrial translocation of BAD and caspase-mediated apoptosis in human prostate cancer cells. Carcinogenesis.

[373] Wang C.G., Zhong L., Liu Y.L., Shi X.J., Shi L.Q., Zeng L., Liu B.Z. (2017). Emodin exerts an antiapoptotic effect on human chronic myelocytic leukemia K562 cell lines by targeting the PTEN/PI3K-AKT signaling pathway and deleting BCR-ABL. Integr. Cancer Ther..

[374] Yao D., Pan D., Zhen Y., Huang J., Wang J., Zhang J., He Z. (2020). Ferulin C triggers potent PAK1 and p21-mediated anti-tumor effects in breast cancer by inhibiting Tubulin polymerization in vitro and in vivo. Pharmacol. Res..

[375] Li J.M., Li W.Y., Huang M.Y., Zhang X.Q. (2015). Fisetin, a dietary flavonoid induces apoptosis via modulating the MAPK and PI3K/Akt signalling pathways in human osteosarcoma (U-2 OS) cells. Bangladesh J. Pharmacol..

[376] Chiang Y.F., Tsai C.H., Chen H.Y., Wang K.L., Chang H.Y., Huang Y.J., Hong Y.H., Ali M., Shieh T.M., Huang T.C. (2021). Protective effects of fucoxanthin on hydrogen peroxide-induced calcification of heart valve interstitial cells. Mar. Drugs.

[377] Nabavi S.F., Sureda A., Habtemariam S., Nabavi S.M. (2015). Ginsenoside Rd and ischemic stroke; a short review of literatures. J. Ginseng. Res..

[378] Li C., Gao H., Feng X., Bi C., Zhang J., Yin J. (2020). Ginsenoside Rh2 impedes proliferation and migration and induces apoptosis by regulating NF-kappaB, MAPK, and PI3K/Akt/mTOR signaling pathways in osteosarcoma cells. J. Biochem. Mol. Toxicol..

[379] Yao Y., Sun S., Cao M., Mao M., He J., Gai Q., Qin Y., Yao X., Lu H., Chen F. (2020). Grincamycin B functions as a potent inhibitor for glioblastoma stem cell via targeting RHOA and PI3K/AKT. ACS Chem. Neurosci..

[380] Wang H.C., Hu H.H., Chang F.R., Tsai J.Y., Kuo C.Y., Wu Y.C., Wu C.C. (2019). Different effects of 4beta-hydroxywithanolide E and withaferin A, two withanolides from Solanaceae plants, on the Akt signaling pathway in human breast cancer cells. Phytomedicine.

[381] Lai H., Wang Y., Duan F., Li Y., Jiang Z., Luo L., Liu L., Leung E.L.H., Yao X. (2018). Krukovine suppresses KRAS-mutated lung cancer cell growth and proliferation by inhibiting the RAF-ERK pathway and inactivating AKT pathway. Front. Pharmacol..

[382] Won Y.S., Seo K.I. (2020). Lupiwighteone induces caspase-dependent and -independent apoptosis on human breast cancer cells via inhibiting PI3K/Akt/mTOR pathway. Food Chem. Toxicol..

[383] Kim G.D. (2017). Myricetin inhibits angiogenesis by inducing apoptosis and suppressing PI3K/Akt/mTOR signaling in endothelial cells. J. Cancer Prev..

[384] Chen Y., Tang Y., Tang Y., Yang Z., Ding G. (2019). Serine protease from *Nereis virens* inhibits H1299 lung cancer cell proliferation via the PI3K/AKT/mTOR pathway. Mar. Drugs.

[385] Yang X., Niu B., Wang L., Chen M., Kang X., Wang L., Ji Y., Zhong J. (2016). Autophagy inhibition enhances colorectal cancer apoptosis induced by dual phosphatidylinositol 3-kinase/mammalian target of rapamycin inhibitor NVP-BEZ235. Oncol. Lett..

[386] Park K.R., Lee H., Kim S.H., Yun H.M. (2022). Paeoniflorigenone regulates apoptosis, autophagy, and necroptosis to induce anti-cancer bioactivities in human head and neck squamous cell carcinomas. J. Ethnopharmacol..

[387] Zeng Y., Yang Y. (2018). Piperine depresses the migration progression via downregulating the Akt/mTOR/MMP9 signaling pathway in DU145 cells. Mol. Med. Rep..

[388] Kumar S., Agnihotri N. (2019). Piperlongumine, a piper alkaloid targets Ras/PI3K/Akt/mTOR signaling axis to inhibit tumor cell growth and proliferation in DMH/DSS induced experimental colon cancer. Biomed. Pharmacother..

[389] Prasad R., Vaid M., Katiyar S.K. (2012). Grape proanthocyanidin inhibit pancreatic cancer cell growth in vitro and in vivo through induction of apoptosis and by targeting the PI3K/Akt pathway. PLoS ONE.

[390] Zhang R., Yu Q., Lu W., Shen J., Zhou D., Wang Y., Gao S., Wang Z. (2019). Grape seed procyanidin B2 promotes the autophagy and apoptosis in colorectal cancer cells via regulating PI3K/Akt signaling pathway. Onco Targets Ther..

[391] Song X.Y., Han F.Y., Chen J.J., Wang W., Zhang Y., Yao G.D., Song S.J. (2019). Timosaponin AIII, a steroidal saponin, exhibits anti-tumor effect on taxol-resistant cells in vitro and in vivo. Steroids.

[392] Wang J., Wang A., He H., She X., He Y., Li S., Liu L., Luo T., Huang N., Luo H. (2019). Trametenolic acid B protects against cerebral ischemia and reperfusion injury through modulation of microRNA-10a and PI3K/Akt/mTOR signaling pathways. Biomed. Pharmacother..

[393] Zamanian M., Bazmandegan G., Sureda A., Sobarzo-Sanchez E., Yousefi-Manesh H., Shirooie S. (2021). The protective roles and molecular mechanisms of troxerutin (vitamin P4) for the treatment of chronic diseases: A mechanistic review. Curr. Neuropharmacol..

[394] Bonel-Perez G.C., Perez-Jimenez A., Gris-Cardenas I., Parra-Perez A.M., Lupianez J.A., Reyes-Zurita F.J., Siles E., Csuk R., Peragon J., Rufino-Palomares E.E. (2020). Antiproliferative and pro-apoptotic effect of uvaol in human hepatocarcinoma HepG2 cells by affecting G0/G1 cell cycle arrest, ROS production and AKT/PI3K signaling pathway. Molecules.

[395] Li X., Tang Y., Yu F., Sun Y., Huang F., Chen Y., Yang Z., Ding G. (2018). Inhibition of prostate cancer DU-145 cells proliferation by *Anthopleura anjunae* oligopeptide (YVPGP) via PI3K/AKT/mTOR signaling pathway. Mar. Drugs.

[396] Lou C., Xu X., Chen Y., Zhao H. (2019). Alisol A suppresses proliferation, migration, and invasion in human breast cancer MDA-MB-231 cells. Molecules.

[397] Chu Y.L., Ho C.T., Chung J.G., Rajasekaran R., Sheen L.Y. (2012). Allicin induces p53-mediated autophagy in Hep G2 human liver cancer cells. J. Agric. Food Chem..

[398] Hu S., Yin J., Yan S., Hu P., Huang J., Zhang G., Wang F., Tong Q., Zhang Y. (2021). Chaetocochin J, an epipolythiodioxopiperazine alkaloid, induces apoptosis and autophagy in colorectal cancer via AMPK and PI3K/AKT/mTOR pathways. Bioorg. Chem..

[399] Tian L., Cheng F., Wang L., Qin W., Zou K., Chen J. (2019). CLE-10 from *Carpesium abrotanoides* L. suppresses the growth of human breast cancer cells (MDA-MB-231) in vitro by inducing apoptosis and pro-death autophagy via the PI3K/Akt/mTOR Signaling Pathway. Molecules.

[400] Zhang J., Yang S.P., Wang K., Huang Y., Yang N., Yang Z.M., Zheng Z.R., Wang Y.J. (2020). Crocin induces autophagic cell death and inhibits cell invasion of cervical cancer SiHa cells through activation of PI3K/AKT. Ann. Transl. Med..

[401] Hong P., Liu Q.W., Xie Y., Zhang Q.H., Liao L., He Q.Y., Li B., Xu W.W. (2020). Echinatin suppresses esophageal cancer tumor growth and invasion through inducing AKT/mTOR-dependent autophagy and apoptosis. Cell Death Dis..

[402] Park K.R., Leem H.H., Kwon Y.J., Kwon I.K., Hong J.T., Yun H.M. (2022). Falcarindiol stimulates apoptotic and autophagic cell death to attenuate cell proliferation, cell division, and metastasis through the PI3K/AKT/mTOR/p70S6K pathway in human oral squamous cell carcinomas. Am. J. Chin. Med..

[403] Adhami V.M., Syed D.N., Khan N., Mukhtar H. (2012). Dietary flavonoid fisetin: A novel dual inhibitor of PI3K/Akt and mTOR for prostate cancer management. Biochem. Pharmacol..

[404] Younis N.S., Abduldaium M.S., Mohamed M.E. (2020). Protective effect of geraniol on oxidative, inflammatory and apoptotic alterations in isoproterenol-induced cardiotoxicity: Role of the Keap1/Nrf2/HO-1 and PI3K/Akt/mTOR pathways. Antioxidants.

[405] Wang S., Sun X., Jiang L., Liu X., Chen M., Yao X., Sun Q., Yang G. (2016). 6-Gingerol induces autophagy to protect HUVECs survival from apoptosis. Chem. Biol. Interact..

[406] Bai L.Y., Su J.H., Chiu C.F., Lin W.Y., Hu J.L., Feng C.H., Shu C.W., Weng J.R. (2021). Antitumor effects of a sesquiterpene derivative from marine sponge in human breast cancer cells. Mar. Drugs.

[407] Liu C.M., Wu Z., Pan B., An L., Zhu C., Zhou J., Jiang Y. (2021). The antiandrogenic effect of neferine, liensinine, and isoliensinine by inhibiting 5-alpha-reductase and androgen receptor expression via PI3K/AKT signaling pathway in prostate cancer. Pharmazie.

[408] Yang G., Bai Y., Wu X., Sun X., Sun M., Liu X., Yao X., Zhang C., Chu Q., Jiang L. (2018). Patulin induced ROS-dependent autophagic cell death in human hepatoma G2 cells. Chem. Biol. Interact..

[409] Kim W.K., Pyee Y., Chung H.J., Park H.J., Hong J.Y., Son K.H., Lee S.K. (2016). Antitumor activity of spicatoside A by modulation of autophagy and apoptosis in human colorectal cancer cells. J. Nat. Prod..

[410] Dan V.M., Muralikrishnan B., Sanawar R., JS V., Burkul B.B., Srinivas K.P., Lekshmi A., Pradeep N.S., Dastager S.G., Santhakumari B. (2018). Streptomyces sp metabolite(s) promotes Bax mediated intrinsic apoptosis and autophagy involving inhibition of mTOR pathway in cervical cancer cell lines. Sci. Rep..

[411] Lv C., Zeng H.W., Wang J.X., Yuan X., Zhang C., Fang T., Yang P.M., Wu T., Zhou Y.D., Nagle D.G. (2018). The antitumor natural product tanshinone IIA inhibits protein kinase C and acts synergistically with 17-AAG. Cell Death Dis..

[412] Lee H.S., Kim E.N., Jeong G.S. (2021). Aromadendrin protects neuronal cells from methamphetamine-induced neurotoxicity by regulating endoplasmic reticulum stress and PI3K/Akt/mTOR signaling pathway. Int. J. Mol. Sci..

[413] Hsu H.S., Liu C.C., Lin J.H., Hsu T.W., Hsu J.W., Su K., Hung S.C. (2017). Involvement of ER stress, PI3K/AKT activation, and lung fibroblast proliferation in bleomycin-induced pulmonary fibrosis. Sci. Rep..

[414] Zhao G., Zhang X., Wang H., Chen Z. (2020). Beta carotene protects H9c2 cardiomyocytes from advanced glycation end product-induced endoplasmic reticulum stress, apoptosis, and autophagy via the PI3K/Akt/mTOR signaling pathway. Ann. Transl. Med..

[415] Guo C., Zhang J., Zhang P., Si A., Zhang Z., Zhao L., Lv F., Zhao G. (2019). Ginkgolide B ameliorates myocardial ischemia reperfusion injury in rats via inhibiting endoplasmic reticulum stress. Drug Des. Devel. Ther..

[416] Xu X., Huang E., Luo B., Cai D., Zhao X., Luo Q., Jin Y., Chen L., Wang Q., Liu C. (2018). Methamphetamine exposure triggers apoptosis and autophagy in neuronal cells by activating the C/EBPbeta-related signaling pathway. FASEB J..

[417] Nie X., Tang W., Zhang Z., Yang C., Qian L., Xie X., Qiang E., Zhao J., Zhao W., Xiao L. (2020). Procyanidin B2 mitigates endothelial endoplasmic reticulum stress through a PPARdelta-dependent mechanism. Redox Biol..

[418] Sinha K., Chowdhury S., Banerjee S., Mandal B., Mandal M., Majhi S., Brahmachari G., Ghosh J., Sil P.C. (2019). Lupeol alters viability of SK-RC-45 (Renal cell carcinoma cell line) by modulating its mitochondrial dynamics. Heliyon.

[419] Saleem M., Afaq F., Adhami V.M., Mukhtar H. (2004). Lupeol modulates NF-kappaB and PI3K/Akt pathways and inhibits skin cancer in CD-1 mice. Oncogene.

[420] Hossan M.S., Chan Z.Y., Collins H.M., Shipton F.N., Butler M.S., Rahmatullah M., Lee J.B., Gershkovich P., Kagan L., Khoo T.J. (2019). Cardiac glycoside cerberin exerts anticancer activity through PI3K/AKT/mTOR signal transduction inhibition. Cancer Lett..

[421] Salehi B., Quispe C., Chamkhi I., El Omari N., Balahbib A., Sharifi-Rad J., Bouyahya A., Akram M., Iqbal M., Docea A.O. (2020). Pharmacological properties of chalcones: A review of preclinical including molecular mechanisms and clinical evidence. Front. Pharmacol..

[422] Liu J., Liu X., Ma W., Kou W., Li C., Zhao J. (2018). Anticancer activity of cucurbitacin-A in ovarian cancer cell line SKOV3 involves cell cycle arrest, apoptosis and inhibition of mTOR/PI3K/Akt signaling pathway. J. BUON.

[423] Reddy D., Kumavath R., Ghosh P., Barh D. (2019). Lanatoside C induces G2/M cell cycle arrest and suppresses cancer cell growth by attenuating MAPK, Wnt, JAK-STAT, and PI3K/AKT/mTOR signaling pathways. Biomolecules.

[424] Mohiuddin M., Kasahara K. (2021). Paclitaxel impedes EGFR-mutated PC9 cell growth via reactive oxygen species-mediated DNA damage and EGFR/PI3K/AKT/mTOR signaling pathway suppression. Cancer Genom. Proteom..

[425] Reddy D., Kumavath R., Tan T.Z., Ampasala D.R., Kumar A.P. (2020). Peruvoside targets apoptosis and autophagy through MAPK Wnt/beta-catenin and PI3K/AKT/mTOR signaling pathways in human cancers. Life Sci..

[426] Rossetti A., Petragnano F., Milazzo L., Vulcano F., Macioce G., Codenotti S., Cassandri M., Pomella S., Cicchetti F., Fasciani I. (2021). Romidepsin (FK228) fails in counteracting the transformed phenotype of rhabdomyosarcoma cells but efficiently radiosensitizes, *in vitro* and *in vivo*, the alveolar phenotype subtype. Int. J. Radiat. Biol..

[427] Reddy D., Ghosh P., Kumavath R. (2019). Strophanthidin attenuates MAPK, PI3K/AKT/mTOR, and Wnt/beta-catenin signaling pathways in human cancers. Front. Oncol..

[428] Wang T., Zou J., Wu Q., Wang R., Yuan C.L., Shu J., Zhai B.B., Huang X.T., Liu N.Z., Hua F.Y. (2021). Tanshinone IIA derivatives induced S-phase arrest through stabilizing c-myc G-quadruplex DNA to regulate ROS-mediated PI3K/Akt/mTOR pathway. Eur. J. Pharmacol..

[429] Huang Y.H., Lei J., Yi G.H., Huang F.Y., Li Y.N., Wang C.C., Sun Y., Dai H.F., Tan G.H. (2018). Coroglaucigenin induces senescence and autophagy in colorectal cancer cells. Cell Prolif..

[430] Sai X., Qin C., Wu Y., Zhao Y., Bian T. (2020). Downregulation of PTEN mediates bleomycin-induced premature senescence in lung cancer cells by suppressing autophagy. J. Int. Med. Res..

[431] Zhang K., Wu S., Wu H., Liu L., Zhou J. (2021). Effect of the Notch1-mediated PI3K-Akt-mTOR pathway in human osteosarcoma. Aging (Albany NY).

[432] Kim S.G., Sung J.Y., Kim J.R., Choi H.C. (2021). Fisetin-induced PTEN expression reverses cellular senescence by inhibiting the mTORC2-Akt Ser473 phosphorylation pathway in vascular smooth muscle cells. Exp. Gerontol..

[433] Chen H.W., Liu M.Q., Zhang G.Z., Zhang C.Y., Wang Z.H., Lin A.X., Kang J.H., Liu W.Z., Guo X.D., Wang Y.D. (2022). Proanthocyanidins inhibit the apoptosis and aging of nucleus pulposus cells through the PI3K/Akt pathway delaying intervertebral disc degeneration. Connect. Tissue Res..

[434] Nasirzadeh M., Rasmi Y., Rahbarghazi R., Kheradmand F., Karimipour M., Aramwit P., Astinfeshan M., Gholinejad Z., Daeihasani B., Saboory E. (2019). Crocetin promotes angiogenesis in human endothelial cells through PI3k-Akt-eNOS signaling pathway. Excli J..

[435] Xiao Y., Liu Y., Gao Z., Li X., Weng M., Shi C., Wang C., Sun L. (2021). Fisetin inhibits the proliferation, migration and invasion of pancreatic cancer by targeting PI3K/AKT/mTOR signaling. Aging (Albany NY).

[436] Khan N., Afaq F., Syed D.N., Mukhtar H. (2008). Fisetin, a novel dietary flavonoid, causes apoptosis and cell cycle arrest in human prostate cancer LNCaP cells. Carcinogenesis.

[437] Chun J., Kim Y.S. (2013). Platycodin D inhibits migration, invasion, and growth of MDA-MB-231 human breast cancer cells via suppression of EGFR-mediated Akt and MAPK pathways. Chem. Biol. Interact..

[438] Mari A., Mani G., Nagabhishek S.N., Balaraman G., Subramanian N., Mirza F.B., Sundaram J., Thiruvengadam D. (2021). Carvacrol promotes cell cycle arrest and apoptosis through PI3K/AKT signaling pathway in MCF-7 breast cancer cells. Chin. J. Integr. Med..

[439] Lin H.Y., Chen Y.S., Wang K., Chien H.W., Hsieh Y.H., Yang S.F. (2017). Fisetin inhibits epidermal growth factor-induced migration of ARPE-19 cells by suppression of AKT activation and Sp1-dependent MMP-9 expression. Mol. Vis..

[440] Chen J., Zhang W., Pan C., Fan J., Zhong X., Tang S. (2021). Glaucocalyxin A induces cell cycle arrest and apoptosis via inhibiting NF-kappaB/p65 signaling pathway in melanoma cells. Life Sci..

[441] Ahmad A., Tiwari R.K., Saeed M., Ahmad I., Ansari I.A. (2022). Glycyrrhizin mediates downregulation of notch pathway resulting in initiation of apoptosis and disruption in the cell cycle progression in cervical cancer cells. Nutr. Cancer.

[442] Ren X., Zhao B., Chang H., Xiao M., Wu Y., Liu Y. (2018). Paclitaxel suppresses proliferation and induces apoptosis through regulation of ROS and the AKT/MAPK signaling pathway in canine mammary gland tumor cells. Mol. Med. Rep..

[443] Zhang Y.B., Zhao Y., Guo J.Y., Cui H.F., Liu S. (2018). Anticancer activity of safranal against colon carcinoma is due to induction of apoptosis and G2/M cell cycle arrest mediated by suppression of mTOR/PI3K/Akt pathway. J. BUON.

[444] Mitra S., Das R., Emran T.B., Labib R.K., Noor E.T., Islam F., Sharma R., Ahmad I., Nainu F., Chidambaram K. (2022). Diallyl disulfide: A bioactive garlic compound with anticancer potential. Front. Pharmacol..

[445] Soltani A., Torki S., Ghahfarokhi M.S., Jami M.S., Ghatrehsamani M. (2019). Targeting the phosphoinositide 3-kinase/AKT pathways by small molecules and natural compounds as a therapeutic approach for breast cancer cells. Mol. Biol. Rep..

[446] Rai R., Gong Essel K., Mangiaracina Benbrook D., Garland J., Daniel Zhao Y., Chandra V. (2020). Preclinical efficacy and involvement of AKT, mTOR, and ERK kinases in the mechanism of sulforaphane against endometrial cancer. Cancers.

[447] Justin S., Rutz J., Maxeiner S., Chun F.K., Juengel E., Blaheta R.A. (2020). Chronic sulforaphane administration inhibits resistance to the mTOR-inhibitor everolimus in bladder cancer cells. Int. J. Mol. Sci..

[448] Wong W.T., Tian X.Y., Huang Y. (2011). Abstract 17733: PPARdelta activation protects endothelial function in diabetic mice via pi3k/akt/enos signaling pathway. Circulation.

[449] Liu H., Mao P., Wang J., Wang T., Xie C.H. (2015). Allicin protects PC12 cells against 6-OHDA-induced oxidative stress and mitochondrial dysfunction via regulating mitochondrial dynamics. Cell Physiol. Biochem..

[450] Mulholland D.J., Tran L.M., Li Y., Cai H., Morim A., Wang S., Plaisier S., Garraway I.P., Huang J., Graeber T.G. (2011). Cell autonomous role of PTEN in regulating castration-resistant prostate cancer growth. Cancer Cell.

[451] Farooqi A.A., Naureen H., Zahid R., Youssef L., Attar R., Xu B. (2021). Cancer chemopreventive role of fisetin: Regulation of cell signaling pathways in different cancers. Pharmacol. Res..

[452] Farhan M., Silva M., Xingan X., Zhou Z., Zheng W. (2021). Artemisinin inhibits the migration and invasion in uveal melanoma via inhibition of the PI3K/AKT/mTOR signaling pathway. Oxidative Med. Cell. Longev..

[453] Chen K., Zhu P., Chen W., Luo K., Shi X.J., Zhai W. (2021). Melatonin inhibits proliferation, migration, and invasion by inducing ROS-mediated apoptosis via suppression of the PI3K/Akt/mTOR signaling pathway in gallbladder cancer cells. Aging (Albany NY).

[454] Ahmad A., Ansari I.A. (2021). Carvacrol exhibits chemopreventive potential against cervical cancer cells via caspase-dependent apoptosis and abrogation of cell cycle progression. Anticancer Agents Med. Chem..

[455] Hou Y.Q., Yao Y., Bao Y.L., Song Z.B., Yang C., Gao X.L., Zhang W.J., Sun L.G., Yu C.L., Huang Y.X. (2016). Juglanthraquinone C induces intracellular ROS increase and apoptosis by Activating the Akt/Foxo signal pathway in HCC cells. Oxidative Med. Cell. Longev..

[456] Kang Y., He P., Wang H., Ye Y., Li X., Xie P., Wu B. (2018). Brazilin induces FOXO3A-dependent autophagic cell death by disturbing calcium homeostasis in osteosarcoma cells. Cancer Chemother. Pharmacol..

[457] Kim W.S., Kim C.H., Lee J.M., Jeon J.H., Kang B.G., Warkad M.S., Inci G., Suh H.W., Lim S.S., Kim S.C. (2021). Purple corn extract (PCE) alleviates cigarette smoke (CS)-induced DNA damage in rodent blood cells by activation of AMPK/Foxo3a/MnSOD pathway. Anim. Cells Syst. (Seoul).

[458] Ji S., Zheng Z., Liu S., Ren G., Gao J., Zhang Y., Li G. (2018). Resveratrol promotes oxidative stress to drive DLC1 mediated cellular senescence in cancer cells. Exp. Cell Res..

[459] Davis R., Singh K.P., Kurzrock R., Shankar S. (2009). Sulforaphane inhibits angiogenesis through activation of FOXO transcription factors. Oncol. Rep..

[460] Ock C.W., Kim G.D. (2021). Harmine hydrochloride mediates the induction of G2/M cell cycle arrest in breast cancer cells by regulating the MAPKs and AKT/FOXO3a signaling pathways. Molecules.

[461] Wu Z., Han X., Tan G., Zhu Q., Chen H., Xia Y., Gong J., Wang Z., Wang Y., Yan J. (2020). Dioscin inhibited glycolysis and induced cell apoptosis in colorectal cancer via promoting c-myc ubiquitination and subsequent hexokinase-2 suppression. Onco Targets Ther..

[462] Hwang S.K., Han S.Y., Jeong Y.J., Magae J., Bae Y.S., Chang Y.C. (2020). 4-O-methylascochlorin activates autophagy by activating AMPK and suppressing c-Myc in glioblastoma. J. Biochem. Mol. Toxicol..

[463] Hsu R.J., Peng K.Y., Hsu W.L., Chen Y.T., Liu D.W. (2022). Z-Ligustilide induces c-Myc-dependent apoptosis via activation of ER-stress signaling in hypoxic oral cancer cells. Front. Oncol..

[464] Gao F.H., Hu X.H., Li W., Liu H., Zhang Y.J., Guo Z.Y., Xu M.H., Wang S.T., Jiang B., Liu F. (2010). Oridonin induces apoptosis and senescence in colorectal cancer cells by increasing histone hyperacetylation and regulation of p16, p21, p27 and c-myc. BMC Cancer.

[465] Lim S.C., Hwang H., Han S.I. (2019). Ellagic acid inhibits extracellular acidity-induced invasiveness and expression of COX1, COX2, Snail, Twist 1, and c-myc in gastric carcinoma cells. Nutrients.

[466] Li H., Luo K., Yang Z., Chen M., Yang X., Wang J., Ying Y., Wu D., Wang Q. (2022). Berbamine suppresses the growth of gastric cancer cells by inactivating the BRD4/c-MYC signaling pathway. Drug Des. Devel. Ther..

[467] Liu J., Huang X., Liu D., Ji K., Tao C., Zhang R., Chen J. (2021). Demethyleneberberine induces cell cycle arrest and cellular senescence of NSCLC cells via c-Myc/HIF-1alpha pathway. Phytomedicine.

[468] Liu X., Duan C., Ji J., Zhang T., Yuan X., Zhang Y., Ma W., Yang J., Yang L., Jiang Z. (2017). Cucurbitacin B induces autophagy and apoptosis by suppressing CIP2A/PP2A/mTORC1 signaling axis in human cisplatin resistant gastric cancer cells. Oncol Rep..

[469] Seo S.U., Woo S.M., Lee H.S., Kim S.H., Min K.J., Kwon T.K. (2018). mTORC1/2 inhibitor and curcumin induce apoptosis through lysosomal membrane permeabilization-mediated autophagy. Oncogene.

[470] Guha P., Kaptan E., Gade P., Kalvakolanu D.V., Ahmed H. (2017). Tunicamycin induced endoplasmic reticulum stress promotes apoptosis of prostate cancer cells by activating mTORC1. Oncotarget.

[471] Wang Z.X., Ma J., Li X.Y., Wu Y., Shi H., Chen Y., Lu G., Shen H.M., Lu G.D., Zhou J. (2021). Quercetin induces p53-independent cancer cell death through lysosome activation by the transcription factor EB and reactive oxygen species-dependent ferroptosis. Br. J. Pharmacol..

[472] Huang L., Peng B., Nayak Y., Wang C., Si F., Liu X., Dou J., Xu H., Peng G. (2020). Baicalein and baicalin promote melanoma apoptosis and senescence via metabolic inhibition. Front. Cell Dev. Biol..

[473] Gao X., Jiang Y., Xu Q., Liu F., Pang X., Wang M., Li Q., Li Z. (2021). 4-Hydroxyderricin promotes apoptosis and cell cycle arrest through regulating PI3K/AKT/mTOR pathway in hepatocellular cells. Foods.

[474] Pham D.C., Chang Y.C., Lin S.R., Fuh Y.M., Tsai M.J., Weng C.F. (2018). FAK and S6K1 inhibitor, neferine, dually induces autophagy and apoptosis in human neuroblastoma cells. Molecules.

[475] Zhang B., Cao H., Rao G.N. (2005). 15(S)-hydroxyeicosatetraenoic acid induces angiogenesis via activation of PI3K-Akt-mTOR-S6K1 signaling. Cancer Res..

[476] Chang H., Li J., Cao Y., Liu T., Shi S., Chen W. (2018). Bufadienolides from *Venenum bufonis* inhibit mTOR-mediated cyclin D1 and retinoblastoma protein leading to arrest of cell cycle in cancer cells. Evid Based Complement. Alternat. Med..

[477] Yang N., Li C., Li H., Liu M., Cai X., Cao F., Feng Y., Li M., Wang X. (2019). Emodin induced SREBP1-dependent and SREBP1-independent apoptosis in hepatocellular carcinoma cells. Front. Pharmacol..

[478] Gu H.F., Li H.Z., Tang Y.L., Tang X.Q., Zheng X.L., Liao D.F. (2016). Nicotinate-curcumin impedes foam cell formation from THP-1 cells through restoring autophagy flux. PLoS ONE.

[479] Lee G.-H., Peng C., Jeong S.-Y., Park S.-A., Lee H.-Y., Hoang T.-H., Kim J., Chae H.-J. (2021). Ginger extract controls mTOR-SREBP1-ER stress-mitochondria dysfunction through AMPK activation in obesity model. J. Funct. Foods.

[480] Liu Y., Hua W., Li Y., Xian X., Zhao Z., Liu C., Zou J., Li J., Fang X., Zhu Y. (2020). Berberine suppresses colon cancer cell proliferation by inhibiting the SCAP/SREBP-1 signaling pathway-mediated lipogenesis. Biochem. Pharmacol..

[481] Liu Y., Huang J., Zheng X., Yang X., Ding Y., Fang T., Zhang Y., Wang S., Zhang X., Luo X. (2017). Luteolin, a natural flavonoid, inhibits methylglyoxal induced apoptosis via the mTOR/4E-BP1 signaling pathway. Sci Rep.

[482] Lan B., Wan Y.J., Pan S., Wang Y., Yang Y., Leng Q.L., Jia H., Liu Y.H., Zhang C.Z., Cao Y. (2015). Parthenolide induces autophagy via the depletion of 4E-BP1. Biochem. Biophys. Res. Commun..

[483] Hasanain M., Bhattacharjee A., Pandey P., Ashraf R., Singh N., Sharma S., Vishwakarma A., Datta D., Mitra K., Sarkar J. (2015). α-Solanine induces ROS-mediated autophagy through activation of endoplasmic reticulum stress and inhibition of Akt/mTOR pathway. Cell Death Dis..

[484] Bach D.H., Kim S.H., Hong J.Y., Park H.J., Oh D.C., Lee S.K. (2015). Salternamide A suppresses hypoxia-induced accumulation of HIF-1alpha and induces apoptosis in human colorectal cancer cells. Mar. Drugs.

[485] El-Ella D.M.A. (2022). Autophagy/apoptosis induced by geraniol through HIF-1α/BNIP3/beclin-1 signaling pathway in A549 CoCl2 treated cells. Adv. Pharm. Bull.

[486] Zhou X., Zheng Y., Sun W., Zhang Z., Liu J., Yang W., Yuan W., Yi Y., Wang J., Liu J. (2021). D-mannose alleviates osteoarthritis progression by inhibiting chondrocyte ferroptosis in a HIF-2alpha-dependent manner. Cell Prolif..

[487] Kim D.H., Sung B., Kang Y.J., Hwang S.Y., Kim M.J., Yoon J.H., Im E., Kim N.D. (2015). Sulforaphane inhibits hypoxia-induced HIF-1alpha and VEGF expression and migration of human colon cancer cells. Int. J. Oncol..

[488] Jin S., Pang R.P., Shen J.N., Huang G., Wang J., Zhou J.G. (2007). Grifolin induces apoptosis via inhibition of PI3K/AKT signalling pathway in human osteosarcoma cells. Apoptosis.

[489] Niu S.B., Yuan D.D., Jiang X.J., Che Y.S. (2014). 11′-Deoxyverticillin A (C42) promotes autophagy through K-Ras/GSK3 signaling pathway in HCT116 cells. Protein Cell.

[490] Wu Y., Chen Q., Wen B., Wu N., He B., Chen J. (2021). Berberine reduces Aβ42 deposition and tau hyperphosphorylation via ameliorating endoplasmic reticulum stress. Front. Pharmacol..

[491] Zeng W., Zhang W., Lu F., Gao L., Gao G. (2017). Resveratrol attenuates MPP(+)-induced mitochondrial dysfunction and cell apoptosis via AKT/GSK-3beta pathway in SN4741 cells. Neurosci. Lett..

[492] Feng S., Zhou Y., Huang H., Lin Y., Zeng Y., Han S., Huang K., Liu Q., Zhu W., Yuan Z. (2022). Nobiletin induces ferroptosis in human skin melanoma cells through the GSK3beta-mediated Keap1/Nrf2/HO-1 signalling pathway. Front. Genet..

[493] Sklirou A.D., Gaboriaud-Kolar N., Papassideri I., Skaltsounis A.L., Trougakos I.P. (2017). 6-Bromo-indirubin-3’-oxime (6BIO), a Glycogen synthase kinase-3beta inhibitor, activates cytoprotective cellular modules and suppresses cellular senescence-mediated biomolecular damage in human fibroblasts. Sci. Rep..

[494] Boonyarat C., Boonput P., Tongloh N., Kaewamatawong R., Chaiwiwatrakul S., Yenjai C., Waiwut P. (2022). Nordentatin inhibits neuroblastoma cell proliferation and migration through regulation of GSK-3 pathway. Curr. Issues Mol. Biol..

[495] Zhu L., Shen X.B., Yuan P.C., Shao T.L., Wang G.D., Liu X.P. (2020). Arctigenin inhibits proliferation of ER-positive breast cancer cells through cell cycle arrest mediated by GSK3-dependent cyclin D1 degradation. Life Sci..

